# The Multivesicular Bodies (MVBs)-Localized AAA ATPase LRD6-6 Inhibits Immunity and Cell Death Likely through Regulating MVBs-Mediated Vesicular Trafficking in Rice

**DOI:** 10.1371/journal.pgen.1006311

**Published:** 2016-09-12

**Authors:** Xiaobo Zhu, Junjie Yin, Sihui Liang, Ruihong Liang, Xiaogang Zhou, Zhixiong Chen, Wen Zhao, Jing Wang, Weitao Li, Min He, Can Yuan, Koji Miyamoto, Bingtian Ma, Jichun Wang, Peng Qin, Weilan Chen, Yuping Wang, Wenming Wang, Xianjun Wu, Hisakazu Yamane, Lihuang Zhu, Shigui Li, Xuewei Chen

**Affiliations:** 1 State Key Laboratory of Hybrid Rice, Key Laboratory of Major Crop Diseases & Collaborative Innovation Center for Hybrid Rice in Yangtze River Basin, Rice Research Institute, Sichuan Agricultural University at Wenjiang, Chengdu, Sichuan, China; 2 State Key Laboratory of Plant Genomics and National Center for Plant Gene Research, Institute of Genetics and Developmental Biology, Chinese Academy of Sciences, Beijing, China; 3 Department of Biosciences, Faculty of Science and Engineering, Teikyo University, Utsunomiya, Tochigi, Japan; University of California Davis, UNITED STATES

## Abstract

Previous studies have shown that multivesicular bodies (MVBs)/endosomes-mediated vesicular trafficking may play key roles in plant immunity and cell death. However, the molecular regulation is poorly understood in rice. Here we report the identification and characterization of a MVBs-localized AAA ATPase LRD6-6 in rice. Disruption of LRD6-6 leads to enhanced immunity and cell death in rice. The ATPase activity and homo-dimerization of LRD6-6 is essential for its regulation on plant immunity and cell death. An ATPase inactive mutation (LRD6-6^E315Q^) leads to dominant-negative inhibition in plants. The LRD6-6 protein co-localizes with the MVBs marker protein RabF1/ARA6 and interacts with ESCRT-III components OsSNF7 and OsVPS2. Further analysis reveals that LRD6-6 is required for MVBs-mediated vesicular trafficking and inhibits the biosynthesis of antimicrobial compounds. Collectively, our study shows that the AAA ATPase LRD6-6 inhibits plant immunity and cell death most likely through modulating MVBs-mediated vesicular trafficking in rice.

## Author Summary

Plants have evolved sophistical immunity system in fighting against pathogenic micro-organisms including bacteria, fungi and oomycetes. Upon perception of pathogens, the immune system activates rapid cell death, characterized as a form of hypersensitive response typically in and around the infection sites to restrict pathogen invasion and prevent disease development. Recent studies have suggested that MVBs-mediated vesicular trafficking might play key roles in plant immunity and cell death. However, the molecular regulation is poorly known. By using the lesion resembling disease (*lrd*) mutant, *lrd6-6*, which exhibits autoimmunity and spontaneous cell death, we characterized LRD6-6 as a MVBs-localized AAA ATPase. We found that the ATPase LRD6-6 was required for MVBs-mediated vesicular trafficking and inhibited the biosynthesis of antimicrobial compounds for immune response in rice. Both the ATPase activity and homo-dimerization of LRD6-6 were essential for its inhibition on immunity and cell death. The catalytically inactive ATPase, LRD6-6^E315Q^, played dominant-negative effect on inhibition of immunity in plants. In addition, the LRD6-6 protein co-localized with the MVBs-spread marker protein RabF1/ARA6 and also interacted with ESCRT-III components OsSNF7 and OsVPS2. In summary, our study has shown that the AAA ATPase LRD6-6 inhibits plant immunity and cell death most likely through modulating MVBs-mediated vesicular trafficking in rice.

## Introduction

Plants are exposed to a vast diversity of micro-organisms such as bacteria, fungi and oomycetes. To protect themselves from pathogenic plant–microbe interactions, plants have developed a sophisticated innate immunity system [1]. Pattern triggered immunity (PTI) and effector triggered immunity (ETI) are two major layers of an immunity system that shares many common responses to pathogen infection including protein phosphorylation, hormonal change, ion fluxes change, production of reactive oxygen species (ROS), synthesis of antimicrobial compounds, transcriptional activation of pathogenesis-related (PR) genes and cell-wall reinforcement via oxidative cross-linking of cell-wall components and deposition of lignin [2–4]. Cell death, which plays a central role in many plant processes, has been observed in both PTI and ETI [3, 5, 6]. Upon perception of pathogens, the immunity system activates rapid cell death, characterized as a form of hypersensitive response (HR) typically in and around the infection sites to restrict pathogen invasion and prevent disease development [7, 8].

The *lesion resembling disease* (*lrd*) mutants carry a cell death phenotype that mimics HR without pathogen attack and are useful tools for studying immunity and cell death [9, 10]. A large number of *lrd* mutants characterized by enhanced immunity and cell death have been identified in maize [11], *Arabidopsis* [12], rice (*Oryza sativa*) [13], barley [14] and *Brassica oleracea* [15]. In rice, more than 10 genes encoding different proteins have been cloned. These include the heat stress transcription factor SPL7 [16], E3 ubiquitin ligase SPL11 [17], zinc finger protein OsLSD1 [18], hydroperoxide lyase OsHPL3 [19], kinase OsPti1a [20], MAPKKK OsEDR1 [21], NPR1-like protein OsNPR1 [22], acyltransferase-like protein SPL18 [23], cytochrome P450 family protein SPL1 [24], fatty-acid desaturase OsSSI2 [25], clathrin-associated adaptor protein complex 1 medium subunit μ1 (AP1M1), SPL28 [26], coproporphyrinogen III oxidase RLIN1 [27], putative splicing factor 3b subunit 3 (SF3b3) protein SPL5 [28] and double-stranded RNA binding motif containing protein OsLMS [29]. Some of them have been studied in molecular regulation of immunity and cell death, including *Spl11*, which encodes an E3 ubiquitin ligase and is associated with SPIN6 and OsRac1 to negatively modulate immunity and cell death [17, 30]. However, the mechanisms of immunity and cell death deployed by *lrd* mutants remain largely unknown in rice.

Previous studies have shown that protein trafficking mediated by multivesicular bodies (MVBs) is associated with immunity in plants [31, 32]. Upon perception of ligand flagellin flg22, the *Arabidopsis* immune receptor FLAGELLIN SENSING 2 (FLS2) present at the plasma membrane is internalized under regulation of the ESCRT-I components VPS37-1. These results suggest that the protein endocytic sorting at the MVBs is critical for FLS2-mediated immunity [33]. Rice SPL28 inhibits immunity and cell death likely through regulation of post-Golgi trafficking [26]. When *Spl28* is disrupted, rice plants display enhanced immunity and exhibit cell death constitutively [26]. These studies indicate that MVBs-mediated vesicular trafficking may participate in regulation of immunity and cell death in plants.

The AAA (ATPase associated with various cellular activities) ATPase family proteins contain conserved ATPase domains spanning 200–250 residues which cover the Walker A, Walker B and the SRH (Second Region of Homology) motifs that distinguish them from classic p-loop NTPases [34–36]. In the process of MVBs biogenesis, the AAA ATPases are used to disassociate the ESCRT-III complex from the membrane by providing required energy [37, 38]. These ATPases participate in diverse cellular processes including membrane fusion, proteolysis and DNA replication, and MVBs-mediated vesicular trafficking [34]. Recent studies have determined that AAA ATPases are also involved in immunity in both mammals and plants. For example, the human AAA ATPase p97/valosin-containing protein (VCP) is an important host factor in antiviral immunity [39]. The human VPS4A functions as a tumor suppressor in hepatoma cells [40] and VPS4B is involved in drug resistance in multiple myeloma cells [41]. The tobacco AAA ATPase NtAAA1 negatively regulates defense response against the invasion of *Pseudomonas syringae* [42, 43]. In *Arabidopsis*, the AAA ATPase AtOM66 functions as a positive regulator in immunity and cell death [44]. AtSKD1, homologous to VPS4A and VPS4B, has been reported to contribute to vacuolar maintenance and MVBs-mediated vesicular trafficking [45] and likely regulates immunity in *Arabidopsis* [46]. However, little is known about the role of AAA ATPases in immunity in rice.

In this study, we report the identification and characterization of the rice *lrd6-6* mutant, which shows enhanced immunity and spontaneous cell death. Map-based cloning reveals that *Lrd6-6* encodes an AAA ATPase, and disruption of the AAA ATPase LRD6-6 leads to autoimmunity and spontaneous cell death in the *lrd6-6* mutant. The ATPase activity and homo-dimerization of LRD6-6 is essential for its inhibition of immunity and cell death in rice. A catalytically inactive mutation, LRD6-6^E315Q^, plays dominant-negative effect in plants. The LRD6-6 protein mainly spreads on MVBs and interacts with ESCRT-III components OsSNF7 and OsVPS2. Further analysis reveals that biosynthesis of antimicrobial metabolites, including lignin and phytoalexins, is highly activated and the process of the MVBs-mediated vesicular trafficking is largely dysregulated in the *lrd6-6* mutant, suggesting that the accumulation of antimicrobial metabolites resulting from the disruption of the LRD6-6 ATPase is tightly linked with the disordered processes of MVBs-mediated vesicular trafficking. Collectively, our study reveals that the AAA ATPase LRD6-6 regulates immunity and cell death likely by modulating the MVBs-mediated vesicular trafficking process.

## Results

### The *lrd6-6* mutant exhibits spontaneous cell death and enhanced basal defense

**Fig 1:**
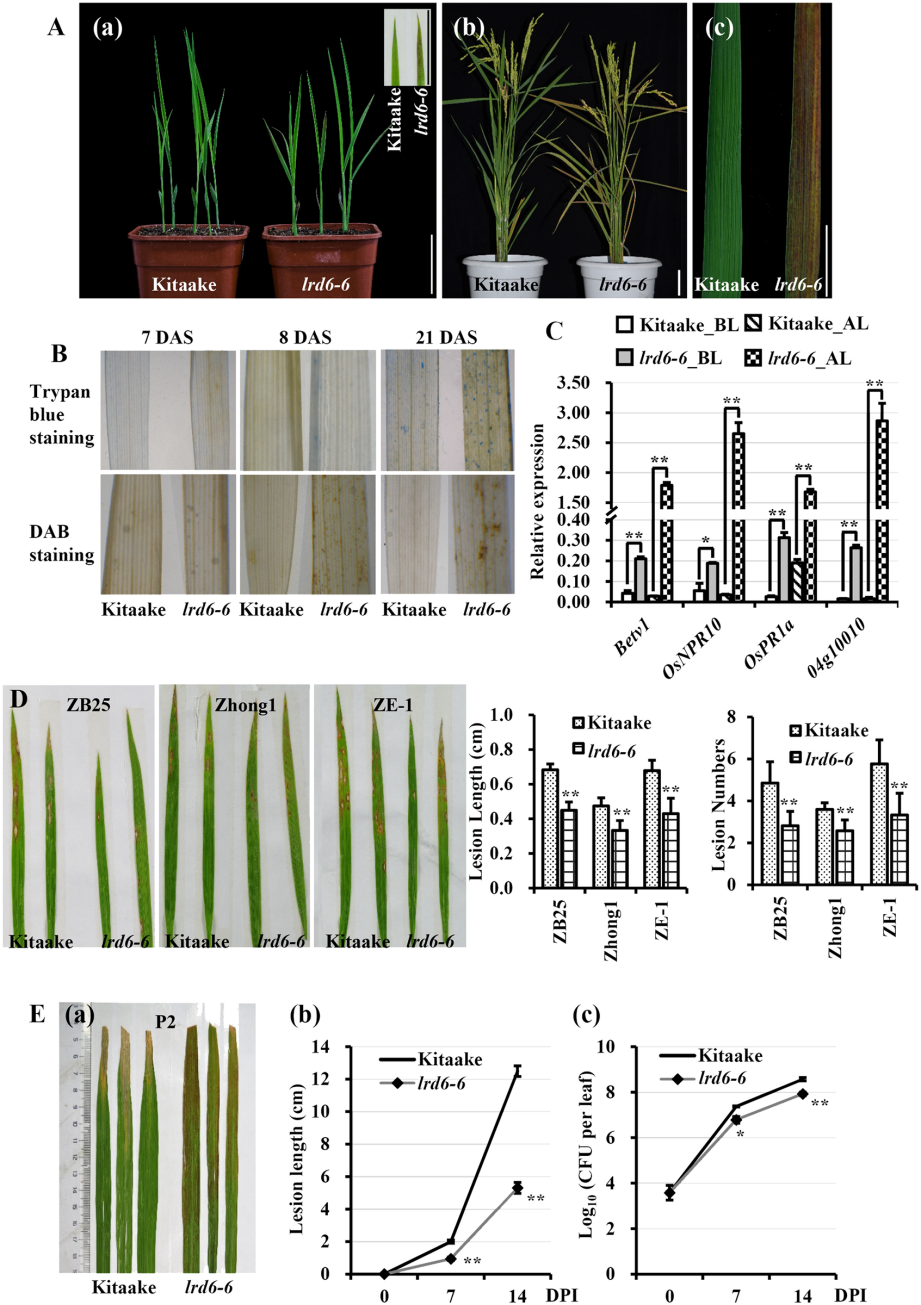
Phenotypic characterization of the *lrd6-6* mutant. (A) Photographs of the *lrd6-6* mutant and wild type Kitaake plants. Plants at two-weeks-old (a) and at mature stage (b) are shown. Representative leaves of the *lrd6-6* mutant and Kitaake at mature stage are shown in (c). Bars in (a–c) represent 3 cm. (B) Trypan blue and DAB staining analyses of the *lrd6-6* mutant and Kitaake at 7, 8 and 21 d, respectively, after plant sowing (DAS). (C) Comparison of the expression of the *pathogenesis-related* (*PR*) genes between the *lrd6-6* mutant and wild type Kitaake before (BL) and after (AL) lesion spots appeared on the *lrd6-6* mutant. The expression levels of the *PR* genes were normalized to the *Ubq5* reference gene. Error bars represent the standard deviations (SDs) for three biology repeats and the expression differences was determined by Student’s *t*-test (**, P < = 0.01). (D) Determination of the resistance of the *lrd6-6* mutant to blast disease. Photographs of representative leaves were respectively taken at 7 d post-inoculation with *M*. *oryzae* strains, ZB25, Zhong-1 and ZE-1. Statistical analyses of the disease lesion lengths and lesion numbers were respectively performed on the leaves of inoculated Kitaake and *lrd6-6* (error bar, SEM, n > 30). Asterisks denote a significant difference from the wild type as determined by Student’s *t*-test (**, P < = 0.01). (E) Determination of the resistance of the *lrd6-6* to bacterial blight disease. Photographs of representative leaves were taken at 15 d post-inoculation (DPI) with *Xoo* strain P2, which is compatible with Kitaake (a). Disease lesion lengths (b) and bacterial populations (c) of the *lrd6-6* mutant and Kitaake were measured at 0, 7 and 14 DPI (error bar, SEM; n > 10 for lesion lengths and n = 5 for bacterial populations). Asterisks denote a significant difference from the wild type as determined by Student’s *t*-test (*, P < = 0.05; **, P < = 0.01).

The *lrd6-6* mutant was generated from tissue culture of rice cv. Kitaake. Plants of *lrd6-6* exhibit reddish-brown lesion spots on the leaves about two weeks after sowing (Fig 1Aa) and the lesion spots expand through the entire plants along with development (Fig 1Ab and 1Ac). The *lrd6-6* plants also exhibit lesion spots when grown in sterile ½ Murashige and Skoog (MS) medium in Solo cups (S1 Fig), suggesting that the occurrence of lesion spots in *lrd6-6* is spontaneous in the absence of any biotic or abiotic stresses. The leaves of *lrd6-6* shaded with silver paper also show lesion spots (S2 Fig), indicating that lesion spots formation in *lrd6-6* is light independent. The lesion spots lead directly to the decrease of photosynthetic pigments in the *lrd6-6* mutant because the contents of chlorophyll a (Chla), chlorophyll b (Chlb) and carotenoid (Car) are dramatically reduced (S3 Fig) in *lrd6-6* after lesion appearance.

To determine if cell death occurred in the *lrd6-6* mutant, we stained rice leaves using trypan blue. The blue staining spots were present in 8-d-old *lrd6-6* plants even before initiation of lesion spots, and more blue staining spots accumulated along with plant development (Fig 1B, upper panel). However, no blue staining spots appeared in leaves of Kitaake (Fig 1B, upper panel). Further, DAB (3, 3’-diamiobenzidine) analysis detected extensive stains in leaves of *lrd6-6* before lesion spots appearance. On the contrary, almost no staining occurred in Kitaake along with plant development (Fig 1B, lower panel), indicating that excess hydrogen peroxide had accumulated in the *lrd6-6* mutant compared with Kitaake. We then sectioned the leaves of *lrd6-6* and Kitaake, and observed under transmission electron microscopy. The subcellular structures were severely degraded in the lesion-spotted parts of leaves but not in other leaf parts in absence of lesion spots in *lrd6-6* or the equivalent parts of Kitaake (S4 Fig). These results suggest that spontaneous cell death occurs in the *lrd6-6* mutant, which results in the formation of lesion spots.

Previous studies have shown that cell death in plants is usually mediated by enhanced immunity [12, 47, 48]. We therefore determined the expression levels of immunity related genes, such as the *PR* genes, *Betv1*, *OsNPR10*, *OsPR1a* and *04g10010* [30, 49, 50]. The expression of these *PR* genes all increased in *lrd6-6* compared with Kitaake plants, predominantly after the presence of cell death (Fig 1C). We then challenged *lrd6-6* plants with the fungal pathogen *Magnaporthe oryzae* (*M*. *oryzae*) and the bacterial pathogen *Xanthomonas oryzae* pv *oryzae* (*Xoo*), which cause blast and bacterial blight diseases, respectively. The disease lesion length and the number of lesions on leaves inoculated with *M*. *oryzae* strains (ZB25, Zhong1 and ZE-1) compatible with Kitaake, were all dramatically reduced in the *lrd6-6* mutant compared with Kitaake (Fig 1D). When inoculated with compatible *Xoo* strains (P2, P4, P5, P6 and Xoo-4), the *lrd6-6* mutant also exhibited enhanced resistance, showing much shorter disease lesion length than Kitaake plants (Figs 1E and S5). Collectively, our results indicate that the rice *lrd6-6* mutant possesses enhanced immunity, which may be mediated by the cell death in the *lrd6-6* mutant.

### *Lrd6-6* encodes a putative AAA ATPase

**Fig 2:**
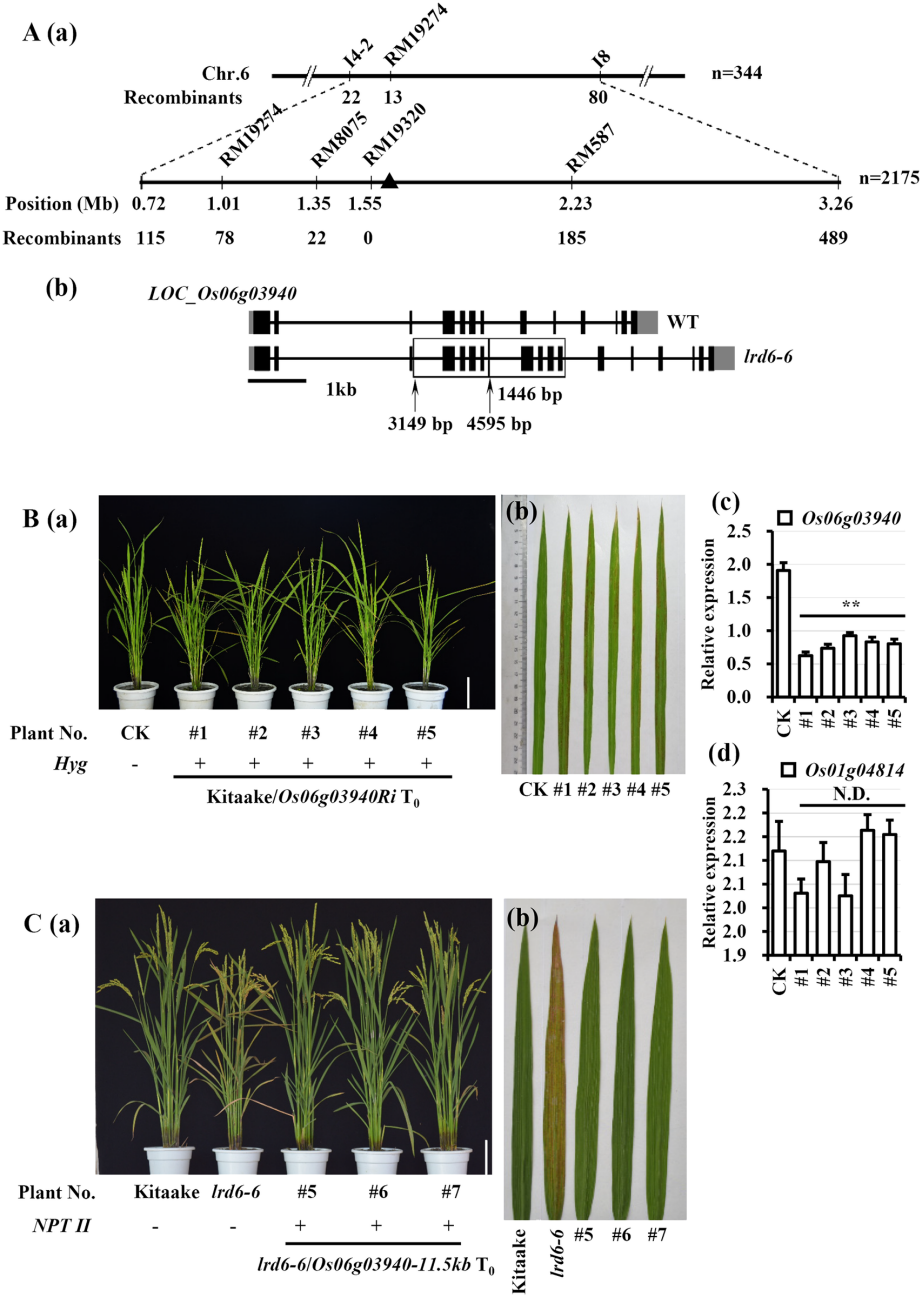
Positional cloning of *Lrd6-6*. (A) Fine mapping of the *Lrd6-6* locus. The *Lrd6-6* locus was delimited to a 93 kb interval between SSR markers RM8075 and RM587 on chromosome 6 (a). The molecular markers and the number of recombinants are shown. A structure variation (SV) was identified from the target gene *Os06g03940* in the *lrd6-6* mutant. The 1446 bp tandem repeat was found in the gene *Os06g03940* which resulted in an additional insertion of 534 bp in the coding DNA of *Os06g03940* in *lrd6-6* (b). The SV mutation was detected by a whole genome resequencing approach and was confirmed by PCR-based sequencing. (B) Plants of RNAi-mediated silencing of *Lrd6-6* exhibited spontaneous cell death phenotypes. The photographs of five representative transgenic T_0_ lines with cell death (#1–#5) and the control (CK) Kitaake are shown (a). Representative leaves from the plants indicated in (a) are shown in (b). Transcriptional expression levels of *Os06g03940* and *LOC_Os01g04814* which shares the highest identity with *Os06g03940* in cDNA sequence in rice plants were respectively determined (c) and (d). PCR-based genotyping with the primer pair specific for the *hygromycin* (*Hyg*) gene was performed to determine whether the plants contained (represented by ‘+’) or lacked (represented by ‘-’) the transgene *Os06g03940Ri*. The relative expressions of the genes were normalized to the *Ubq5* reference gene. The error bars represent the SDs of three biology repeats and the expression differences was determined by Student’s *t*-test (*, P < = 0.05; **, P < = 0.01; N.D., No significantly difference). (C) An 11.5 kb wild type genomic segment of *Lrd6-6* completely rescued the cell death phenotype of the *lrd6-6* mutant. Photograph of three independent representative T_0_ plants carrying the genomic DNA fragment of *Lrd6-6* (*Os06g03940-11*.*5kb*) in the *lrd6-6* mutant genetic background (a)–Kitaake and the *lrd6-6* mutant are also included in the photograph. PCR-based genotyping of the *Neomycin phosphotransferase II* (*NPT II*) gene indicated that the plants contained (‘+’) or lacked (‘-’) the transgenic genome DNA fragment *Os06g03940-11*.*5kb*. Representative leaves from the plants indicated in (a) are shown in (b). Bars = 10 cm.

Since the *lrd* phenotype caused by cell death in *lrd6-6* did not co-segregate with the *hygromycin* (*Hyg*) gene (S6 Fig), we presumed that the spontaneous cell death in the *lrd6-6* mutant might have resulted from tissue-culture induced somatic mutation during transformation. We thus developed three F_2_ populations and performed genetic analysis on the *lrd6-6* locus. Genetic analyses showed that the *lrd6-6* phenotype was controlled by a single recessive nuclear locus (S1 Table). Next, map-based cloning of the *Lrd6-6* gene was performed using 344 F_2_ individuals with the spontaneous cell death phenotype from the cross between the *lrd6-6* mutant and rice 02428. The *Lrd6-6* locus was first mapped in the interval with a physical distance of 2.54 Mb between the InDel markers I4-2 and I8 on chromosome 6 (Fig 2Aa). By using more markers to analyze 2175 F_2_ individuals with the cell death phenotype, the *Lrd6-6* locus was then delimited on the genomic region within 93 kb between RM8075 and RM587 (Fig 2Aa). Next, we sequenced the genomic DNA sample bulked with 30 BC_2_F_3_ individuals with cell death phenotypes using a whole-genome resequencing approach. The genomic DNA of Kitaake was also sequenced as a control. When comparing the sequences of the 93 kb between the bulked DNA sample and the Kitaake control DNA, we found that a 1446 bp DNA fragment from gene *LOC_Os06g03940* (RGAP ID from http://rice.plantbiology.msu.edu, abbreviated to *Os06g03940* hereafter) that spans four exons was tandemly repeated in *Os06g03940* in the bulked DNA sample (Fig 2Ab). Sequencing of cDNA revealed that this 1446 bp tandem repeat resulted in an insertion of 534 bp in the protein-coding sequence of *Os06g03940* that might disrupt the gene function in the *lrd6-6* mutant (S7 Fig). These results indicate that the spontaneous cell death phenotype in the *lrd6-6* mutant was likely resulted from the insertion of 1446 bp tandem repeat in the *Os06g03940* gene, and thus *Os06g03940* encoding an AAA ATPase is likely the target gene of *Lrd6-6*.

We carried out a knockdown experiment to confirm that the *lrd6-6* mutation was caused by *Os06g03940*. We amplified a unique segment (Seg I, 445 bp) covering nucleotides 557 to 1001 of the *Os06g03940* open reading frame (ORF) (S8 Fig) and used this segment to create an RNA interference (RNAi) construct, pANDA–*Os06g03940Ri*. This segment shows only approximately 20.12% identity with the closest homologous gene *Os01g04814* (S8 Fig). This construct was then introduced into Kitaake through *Agrobacterium*-mediated transformation. We found that all 33 transgenic plants with suppressed *Os06g03940* expression displayed spontaneous cell death similar to the *lrd6-6* mutant (Figs 2B and S9A). In these plants, the expression of *Os01g04814*, which shared the highest identity with *Os06g03940* in cDNA sequence, was not suppressed (Fig 2Bd), suggesting that specific silencing of *Os06g03940* resulted in the spontaneous cell death phenotype.

To further verify the result obtained by RNAi analysis, we then cloned an 11.5 kb genomic DNA fragment harboring the native promoter and full coding region of the gene *Os06g03940* from rice Nipponbare and placed it in the binary vector pCactN-XG to create the construct pCactN-XG–*Os06g03940-11*.*5kb*. The DNA fragment *Os06g03940-11*.*5kb* was then introduced into the *lrd6-6* mutant through a similar *Agrobacterium*-mediated transformation approach. All 12 transgenic lines carrying the transgene *Os06g03940-11*.*5kb* no longer exhibited the spontaneous cell death phenotype (Figs 2C and S9B). This reveals that the *Os06g03940-11*.*5kb* transgene restores the spontaneous cell death phenotype in the *lrd6-6* mutant to that of the wild type Kitaake. Taken together, these results clearly demonstrate that *Os06g03940* is the target gene of *lrd6-6*, in which the mutation with the inserted 1446 bp tandem repeat was responsible for the spontaneous cell death phenotype.

Recently, Fekih et al. reported that a G–>A base substitution resulted in a premature translation termination in the *Lmr* gene (RAP ID *Os06g0130000*; http://rapdb.dna.affrc.go.jp), which is the same gene *Os06g03940* from RGAP according to gene ID conversion through ID Converter (http://rapdb.dna.affrc.go.jp/tools/converter), and also led to the spontaneous cell death phenotype in rice [51]. Similar to *lrd6-6*, the *lmr* mutant displayed elevated *PR* gene expression and enhanced disease resistance compared with wild type rice Hitomebore [51]. Fekih and his colleagues also used the RNAi approach targeting Seg II of the gene, which spans a region different from Seg I used in our study (S8 Fig), and a complementary test by overexpressing the full-length cDNA of *Lmr* to confirm their results [51]. Together, these results confirm that disruption of *Os06g03940* leads to the *lrd6-6* phenotype.

### *Lrd6-6* is expressed in diverse tissues in rice and the protein resides mainly on MVBs

To determine the expression pattern of *Lrd6-6*, we respectively sampled the root, stem, leaf and panicle at the two-, four-, six-leaf and mature stages of Kitaake and determined the transcript levels of *Lrd6-6* in these tissues. The quantitative reverse transcription-PCR (qRT-PCR) analysis showed that *Lrd6-6* was expressed in all these tissues with predominance in leaves (S10 Fig).

To investigate the subcellular localization of LRD6-6, we generated construct p35S-*Lrd6-6*–*GFP* expressing the LRD6-6–GFP fusion protein and performed a transient expression assay using *Nicotiana benthamiana* through an agroinfiltration approach. The GFP signal was punctate in the cytoplasm in the leaf of *N*. *benthamiana* expressing LRD6-6–GFP fusion protein whereas, as expected, the GFP fluorescence was distributed in the cytoplasm and nucleus in the leaf expressing GFP alone (S11A Fig). The distribution of the LRD6-6–GFP fusion protein was reminiscent of the localization of the *Arabidopsis* LRD6-6 homologous protein AAA ATPase SKD1, which was previously shown to be located in MVBs [38]. By using bombardment-mediated transformation, we also observed a punctate pattern of GFP signals in onion epidermal cells expressing the LRD6-6–GFP fusion protein (S11B Fig). The *Lrd6-6*–*GFP* transgene was able to restore the *lrd6-6* plants to the wild type level (S12 Fig) suggesting that the LRD6-6–GFP fusion protein functions similarly as LRD6-6. The MVBs-localized pattern of the LRD6-6–GFP protein highly suggests that LRD6-6 localizes in MVBs.

**Fig 3:**
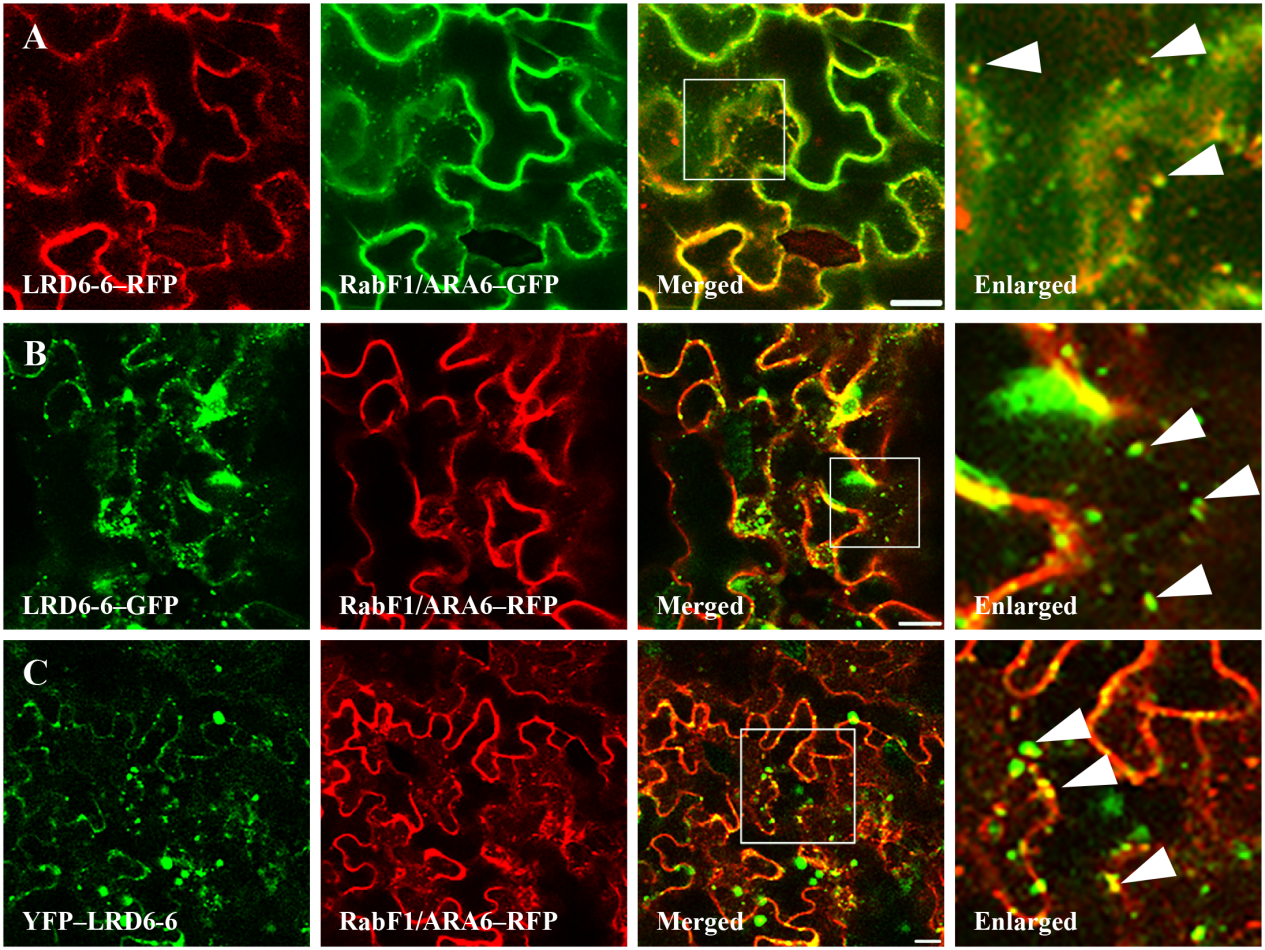
Subcellular localization of the protein LRD6-6. Determination the subcellular localization of protein LRD6-6 fused with fluorescence proteins in *N*. *benthamiana*. The pairs, LRD6-6–RFP and RabF1/ARA6–GFP (A), LRD6-6–GFP and RabF1/ARA6–RFP (B), and YFP–LRD6-6 and RabF1/ARA6–RFP (C) were respectively co-expressed into *N*. *benthamiana* cells. White squares in the merged images are shown as detail pictures (enlarged in the right panels). Arrowheads in the enlarged pictures point to some of the punctate MVBs of which the RabF1/ARA6 and LRD6-6 co-localized. Fluorescence was determined 36 h post transformation. Bars = 20 μm.

The Rab GTPase RabF1/ARA6 protein has been shown to locate on the peripheral membrane of the MVBs and has been widely used as the specific marker protein for plant MVBs-localization analysis [52, 53]. We transiently expressed the fused proteins, RabF1/ARA6–GFP and RabF1/ARA6–RFP respectively in *N*. *benthamiana* cells through the same agroinfiltration approach and found that both RabF1/ARA6–GFP and RabF1/ARA6–RFP are present in punctate patterns in the cells as expected (S13 Fig). When treated with chemical wortmannin which is able to change MVBs into ring-like structures through inhibiting phosphatidylinositol 3-kinase (PI3) activity [54, 55], the punctate GFP or RFP signals in the *N*. *benthamiana* cells expressing RabF1/ARA6–GFP or RabF1/ARA6–RFP were respectively converted into ring-like structures (S13 Fig). These results suggested that RabF1/ARA6–GFP and RabF1/ARA6–RFP were MVBs-localized and could be used as control for MVBs-localization analysis on LRD6-6. Then, we co-transformed LRD6-6–RFP (RFP fused on the C-terminus of LRD6-6) with RabF1/ARA6–GFP, LRD6-6–GFP (GFP fused on the C-terminus of LRD6-6) or YFP–LRD6-6 (YFP fused on the N-terminus of LRD6-6), respectively, with RabF1/ARA6–RFP. Consistently with prior results, the punctate fluorescence signals of the fusion proteins, LRD6-6–GFP, LRD6-6–RFP and YFP–LRD6-6, all co-located with fluorescence signals derived from RabF1/ARA6–GFP or RabF1/ARA6–RFP protein but not the signals of chlorophyll (Figs 3 and S14). Together, our results clearly show that the LRD6-6 protein resides mainly on MVBs which is different from the results of Fekih et al. showing that LRD6-6–GFP was localized in chloroplast [51].

### LRD6-6 possesses ATPase activity

**Fig 4:**
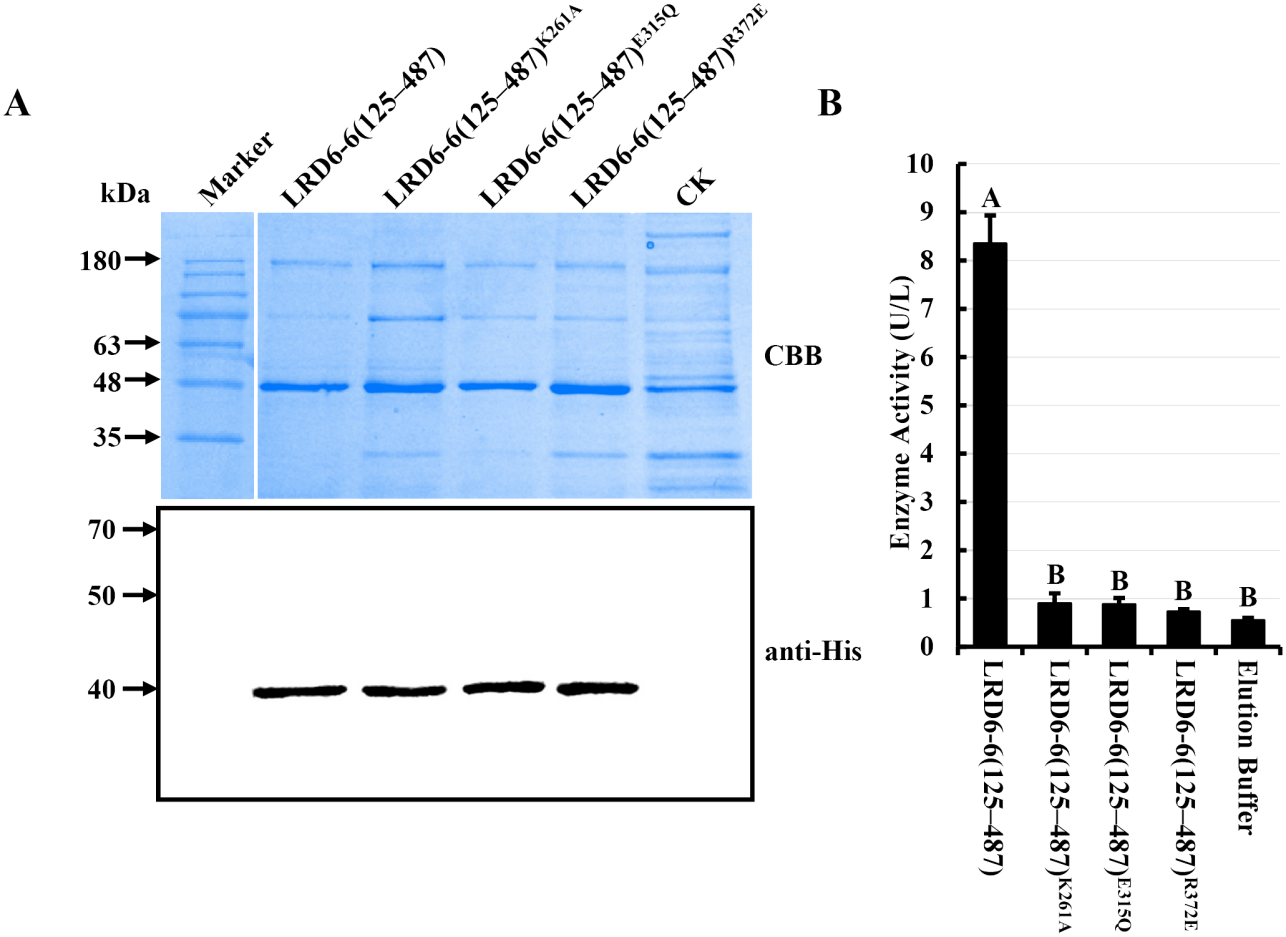
*In vitro* ATPase activity determination of LRD6-6. (A) Detection of the N-terminal truncated recombinant protein His–LRD6-6(125–487), and its variants, His–LRD6-6(125–487)^K261A^, His–LRD6-6(125–487)^E315Q^ and His–LRD6-6(125–487)^R372E^, purified from *E*. *coli* by coomassie brilliant blue staining (upper panel) and western blot with anti-His (lower panel). (B) *In vitro* ATPase assay on recombinant proteins His–LRD6-6(125–487), His–LRD6-6(125–487)^K261A^, His–LRD6-6(125–487)^E315Q^ and His–LRD6-6(125–487)^R372E^. ATPase activities were measured using a malachite green-based colorimetric approach. The ATPase activities with mean values ± SEM of four replications were shown. Statistical significance comparison was conducted with ANOVA (P < = 0.01), where different capital letters above columns indicate significant differences, whereas the same letter indicates no significant differences.

To characterize the LRD6-6 protein, multiple sequence alignment was performed on LRD6-6 and homologous proteins reported in different species. The LRD6-6 is clustered in the same clade with proteins AtSKD1 from *Arabidopsis* [38], ZmSKD1 from *Zea mays* [56], SKD1 from human [57], and VPS4p from yeast [58] (S15A Fig). The results showed that the LRD6-6 protein also contained the conserved domains (Walker A, Walker B and SRH), typical of previously characterized AAA ATPases [59] (S15B Fig), suggesting that LRD6-6 was an AAA ATPase. Previous reports suggest that the residue lysine (K) at 261^st^ in Walker A motif of LRD6-6 is likely essential for nucleotide binding [37], glutamic acid (E) at 315^th^ in Walker B is likely responsible for ATP hydrolysis [37] and arginine (R) at 372^nd^ in SRH is likely vital for both ATP hydrolysis and oligomerization [59] (S15B Fig). To determine the ATPase activity of LRD6-6, the N-terminally truncated LRD6-6 LRD6-6(125–487) (AAs: 125–487, covering the ATPase domain) and its variants carrying point mutations, LRD6-6(125–487)^K261A^, LRD6-6(125–487)^E315Q^ and LRD6-6(125–487)^R372E^, were respectively fused with a His-tag and expressed in *Escherichia coli* (Fig 4A). The purified proteins were then used for *in vitro* ATPase activity assay. The results showed that LRD6-6(125–487) was able to hydrolyze ATP but its variants LRD6-6(125–487)^K261A^, LRD6-6(125–487)^E315Q^ or LRD6-6(125–487)^R372E^ were not (Fig 4B). These results show that LRD6-6 is an active ATPase and that residues K261, E315 and R372 are essential for its ATPase activity.

### LRD6-6 can homo-dimerize in yeast and *in planta*

**Fig 5:**
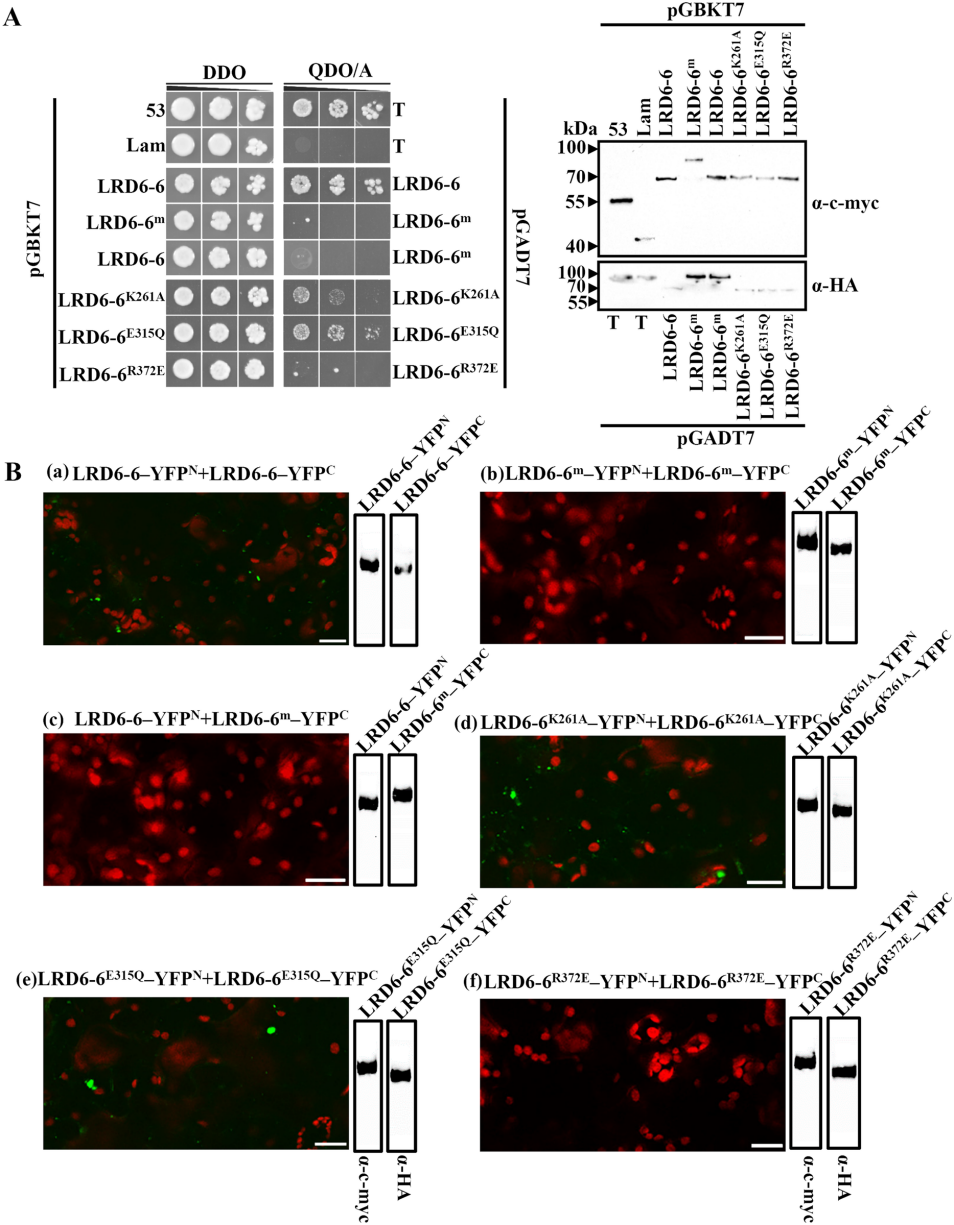
Dimerization of LRD6-6 in yeast and *in planta*. (A) Dimerization test of the LRD6-6 in yeast. The full-length CDSs for LRD6-6, the mutant *lrd6-6* (LRD6-6^m^) and the variants, LRD6-6^K261A^, LRD6-6^E315Q^ and LRD6-6^R372E^ were respectively inserted into the pGPBKT7 (BD) and pGADT7 (AD), and were subjected to Y2H test through co-transformation by pairs (left panel). The pairs, pGBKT7-53/pGADT7-T and pGBKT7-Lam/pGADT7-T, representing positive and negative interactions, respectively, were also included in this test. The positive transformants were diluted in 10-fold serial dilution and spotted on double dropout medium (SD/–Leu/–Trp; DDO) and quadruple dropout medium supplemented with Aureobasidin A (SD/–Ade/–His/–Leu/–Trp/AbA; QDO/A) plates. The growth of co-transformed yeast cells in the medium QDO/A indicates protein–protein interaction in yeast. Western blot analysis with anti-c-myc or anti-HA antibodies was performed to ensure the expression of BD- and AD-fused proteins, respectively, for each co-transformation event (right panel). (B) BiFC analyses on the dimerization of LRD6-6 in *N*. *benthamiana*. The full-length CDSs for LRD6-6, the mutant *lrd6-6* (LRD6-6^m^) and the variants, LRD6-6^K261A^, LRD6-6^E315Q^ and LRD6-6^R372E^ were respectively inserted into the split yellow fluorescence protein (YFP) vectors, split YFP^N^ and split YFP^C^, and were co-transformed into *N*. *benthamiana* cell by pair. The punctate YFP signals represent interaction between the co-expressed proteins, while the red signals represent the auto-fluorescence of chlorophyll. The YFP signals didn’t overlap with the auto-fluorescence, bars = 20 μm. The expression of proteins was detected by anti-c-myc or anti-HA respectively.

Previous reports have shown that the AAA ATPase VPS4/SKD1, which is homologous to LRD6-6, can form dimers to function in disassembly of the ESCRT-III complex in regulation of MVBs biogenesis [37, 58, 60]. To test if LRD6-6 possesses the ability of dimerization, we cloned the full-length coding sequences (CDSs) of *Lrd6-6*, *Lrd6-6*^K261A^, *Lrd6-6*^E315Q^ and *Lrd6-6*^R372E^ respectively into both pGADT7 and pGBKT7 vectors and used for yeast two hybrid (Y2H) analysis. The CDS of *lrd6-6* (*Lrd6-6*^m^) was also cloned into these vectors and included in this analysis. The results showed that the protein LRD6-6 interacted with itself (Fig 5A). Variants LRD6-6^K261A^ and LRD6-6^E315Q^ also interacted with themselves whereas variants LRD6-6^R372E^ and LRD6-6^m^ did not (Fig 5A). We then fused these proteins with the split YFP N-half and split YFP C-half, respectively, and performed bimolecular fluorescence complementation (BiFC) assay in *N*. *benthamiana*. The results showed that the cells of *N*. *benthamiana* co-transformed with LRD6-6–YFP^N^ and LRD6-6–YFP^C^, LRD6-6^K261A^–YFP^N^ and LRD6-6^K261A^–YFP^C^, or LRD6-6^E315Q^–YFP^N^ and LRD6-6^E315Q^–YFP^C^ produced yellow fluorescence signals whereas the cells co-transformed with LRD6-6^m^–YFP^N^ and LRD6-6^m^–YFP^C^, LRD6-6–YFP^N^ and LRD6-6^m^–YFP^C^, or LRD6-6^R372E^–YFP^N^ and LRD6-6^R372E^–YFP^C^, exhibited no fluorescence signals (Fig 5B). These results suggest that LRD6-6 is capable of homo-dimerization and–of the three residues, K261, E315 and R372 –only R372 was required for this dimerization. As residues K261 and E315 are essential for ATPase activity but not required for homo-dimerization, these results also indicate that the ATPase activity is not required for homo-dimerization of LRD6-6. However, residue R372 is required for both homo-dimerization and ATPase activity of LRD6-6, suggesting that homo-dimerization may be required for the ATPase activity of LRD6-6.

### ATPase activity is required for full function of LRD6-6 in rice

**Fig 6:**
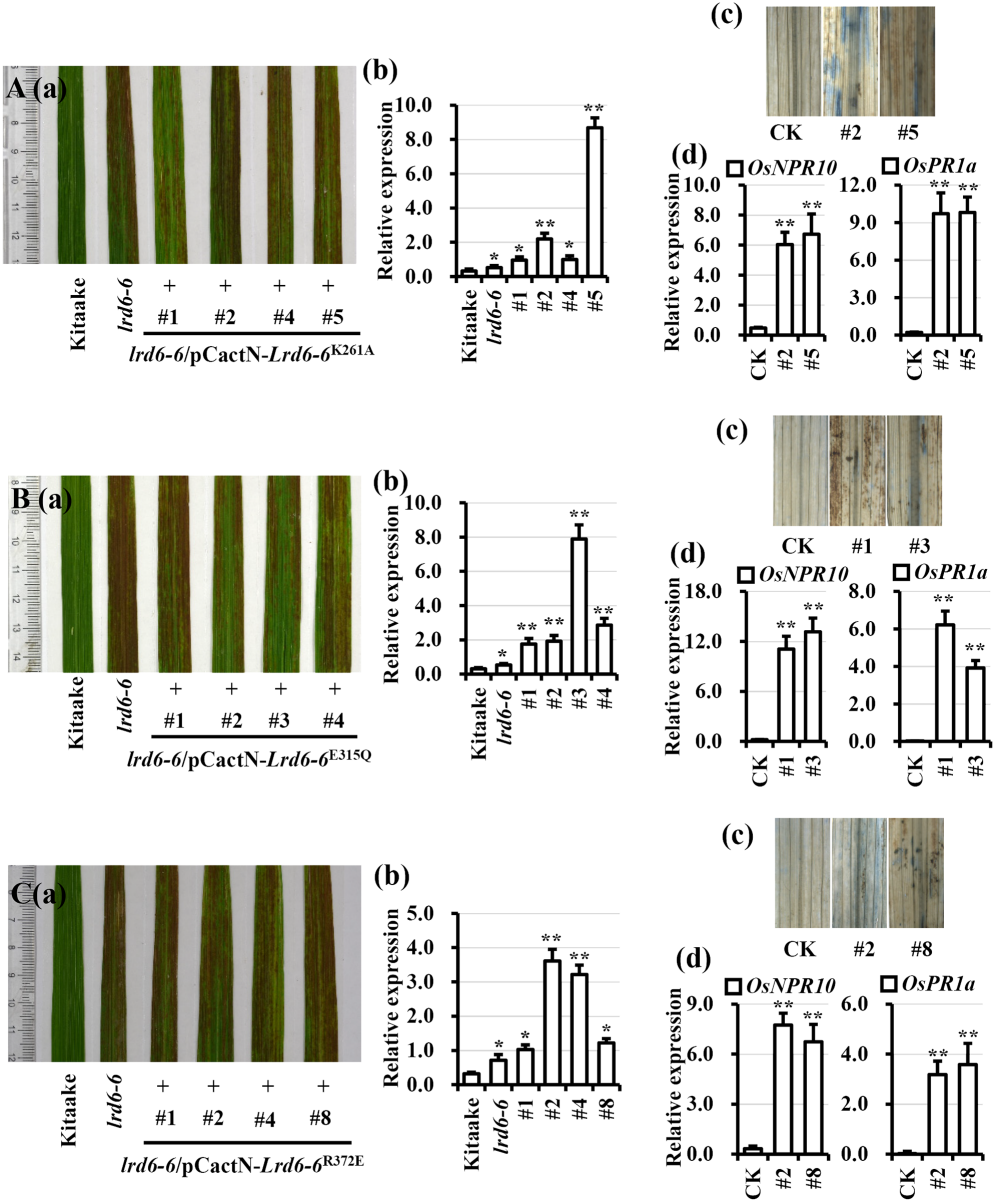
Mutations K261A, E315Q and R372E abolish the full function of LRD6-6 in rice. Expression of *Lrd6-6*^K261A^ (A), *Lrd6-6*^E315Q^ (B) or *Lrd6-6*^R372E^ (C) in the *lrd6-6* mutant genetic background was not able to rescue the autoimmunity and cell death phenotype of *lrd6-6*. For each transformation, leaves (a) from four independent lines with gene expression verified (b) are shown. Of them, two lines were subjected to cell death (c) and *PR* genes expression analyses (d), respectively. The relative expressions of the genes were normalized to the *Ubq5* reference gene. The error bars represent the SDs of three biology repeats and the expression differences was determined by Student’s *t*-test (*, P < = 0.05; **, P < = 0.01).

To determine if the ATPase activity of LRD6-6 is required for its biological function, we created constructs, which carry *Lrd6-6*^K261A^, *Lrd6-6*^E315Q^ or *Lrd6-6*^R372E^ with the ATPase activity knocked out or compromised, and introduced them individually into the *lrd6-6* mutant. Transformation of *Lrd6-6* into *lrd6-6* was also performed as a positive control. The transgenic plants expressing the wild type LRD6-6 were able to restore the *lrd6-6* phenotype to wild type (S16 Fig); however, none of LRD6-6^K261A^, LRD6-6^R372E^ and LRD6-6^E315Q^ restored the mutant (Fig 6). The *lrd6-6* plants expressing the LRD6-6 with catalytically inactive or compromised ATPase activity retained enhanced immunity and spontaneous cell death as the *lrd6-6* mutant (Fig 6). Thus, the ATPase activity is essential for LRD6-6 to inhibit immunity and cell death in rice.

### Expression of the catalytically inactive mutant *Lrd6-6*^E315Q^ leads to autoimmunity and spontaneous cell death in rice

**Fig 7:**
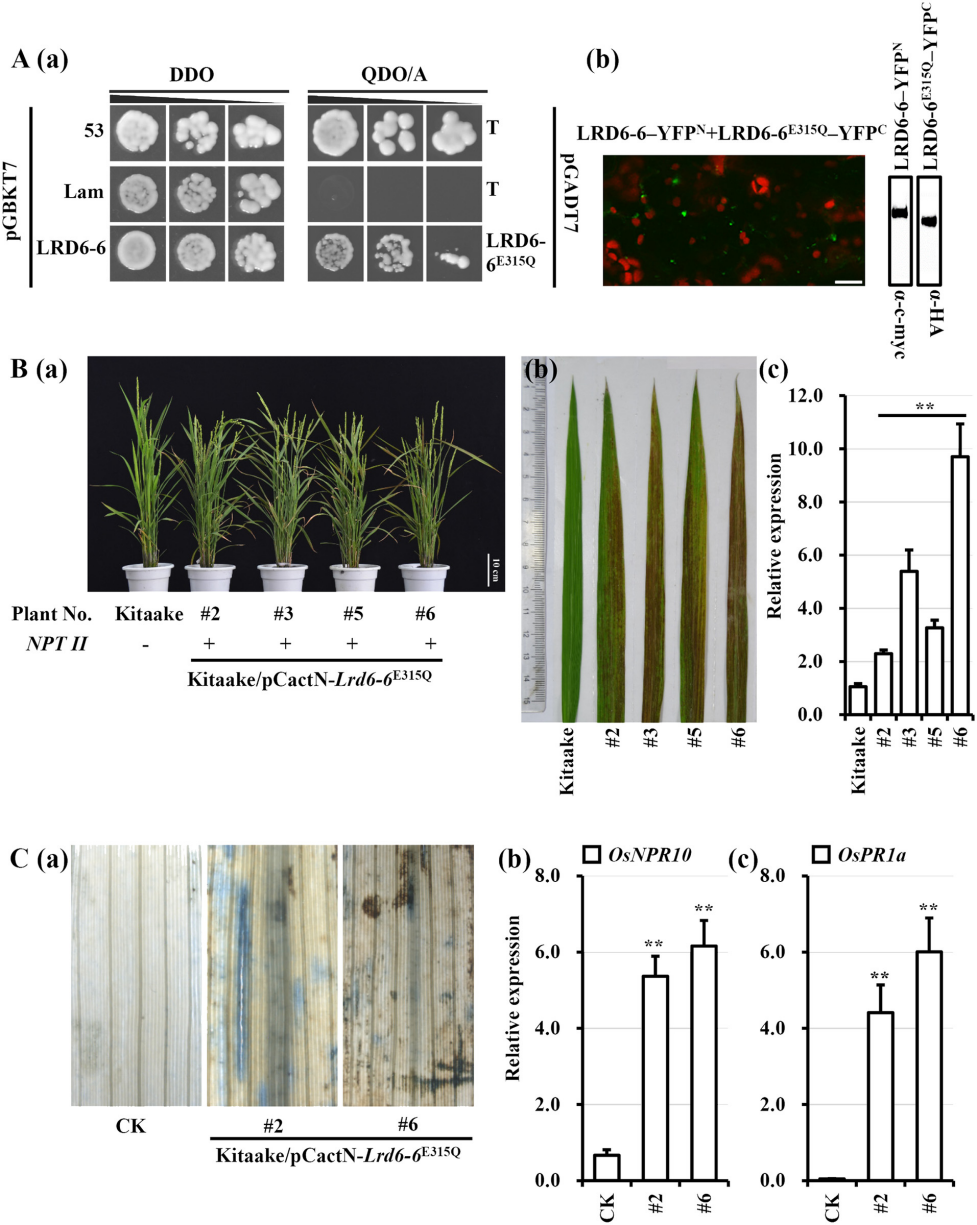
The mutation LRD6-6^E315Q^ plays dominant-negative effect in rice. (A) LRD6-6^E315Q^ interacts with LRD6-6 in yeast and in *N*. *benthamiana*, bars = 20 μm. (B) Expression of *Lrd6-6*^E315Q^ resulted in enhanced immune response and spontaneous cell death in rice Kitaake. Whole individual plants (a), representative leaves (b) and expression levels of the transgenes (c) from four independent transgenic lines are representatively shown. (C) Two transgenic lines, #2 and #6, were used for cell death (a) and *PR* gene expression analyses (b). The relative expressions of the genes were normalized to the *Ubq5* reference gene. The error bars represent the SDs of three biology repeats and the expression differences was determined by Student’s *t*-test (**, P < = 0.01).

The glutamate (E) of hhhhDE sequence (h represents a hydrophobic amino acid) in the Walker B motif is crucial for ATP hydrolysis while the lysine (K) residue in the Walker A consensus sequence GXXXXGK[T/S] (X is any amino acid) of the conserved AAA ATPase family is crucial for ATP binding [36]. Although these two residues are capable of rendering the wild type protein dominant-negative [36], mutation of E in the Walker B motif has been more widely used to create ‘substrate traps’ in yeast [37], mammals [61] and *Arabidopsis* [38]. Because LRD6-6^E315Q^ could dimerize with LRD6-6 as shown in yeast and in *N*. *benthamiana* (Fig 7A), we presumed that LRD6-6^E315Q^ would also play a dominant-negative role for LRD6-6 in rice. To test this hypothesis, we expressed *Lrd6-6*^E315Q^ in wild type Kitaake. The result showed that the transgenic plants expressing LRD6-6^E315Q^ displayed spontaneous cell death similar to the *lrd6-6* mutant plants, while transgenic plants expressing the wild type LRD6-6 did not (Figs 7B and S17). Detection of dead cells using trypan blue and measurement on the expression of *PR* genes indicated that the transgenic plants expressing LRD6-6^E315Q^ also possessed enhanced immunity and presented spontaneous cell death similarly as the *lrd6-6* mutant (Fig 7C). These results show that the LRD6-6^E315Q^ is able to compromise LRD6-6 function likely by forming a functionally inactive homo-dimer, thus acting dominant-negatively. These results also suggest that mutant gene *Lrd6-6*^E315Q^ may be utilized as a gene trap to suppress the inhibitory regulation of the ATPase LRD6-6 in immunity and cell death to enhance plant disease resistance.

### Genes associated with MVBs-mediated vesicular trafficking are dysregulated in the *lrd6-6* mutant

To identify downstream components of the immunity and cell death resulting from disruption of the LRD6-6 ATPase in *lrd6-6*, we performed a genome-wide transcript comparative analysis on *lrd6-6* and Kitaake using an RNA-seq approach. A total of 1223 differentially expressed genes (DEGs) were obtained (S18 Fig). Of these, 980 genes were up-regulated whereas 243 were down-regulated in the *lrd6-6* mutant compared to the wild type Kitaake [P < = 0.05, Log_2_FC (*lrd6-6*/Kitaake) > 1] (S18 Fig).

Gene Ontology (GO) analysis showed that these DEGs could be classified into different cellular components (S19 Fig and S2 Table). Of them, MVBs-mediated vesicular trafficking associated components were the most enriched, including the GO terms membrane, membrane coat, clathrin coat of coated pit, endoplasmic reticulum membrane and clathrin coat of trans-Golgi network vesicle (S19 Fig and S2 Table). We then randomly selected some of these genes, performed qRT-PCR analysis on them and verified their differential expression between *lrd6-6* and Kitaake (S20 Fig). We also investigated the expression of these genes in *lrd6-6* plants transformed with the *Os06g03940-11*.*5kb* transgene and plants expressing *Lrd6-6*^E315Q^ by qRT-PCR analysis. The results showed that expression of *Os06g03940-11*.*5kb* in *lrd6-6* restored the expressions of these genes to the similar levels in the wild type Kitaake plants while expression of *Lrd6-6*^E315Q^ in Kitaake retained dysregulation of these genes similarly as *lrd6-6* (S21 Fig). These results suggested that the dysregulation of these genes in *lrd6-6* is indeed the result of loss of function of the *Os06g03940* (*Lrd6-6*) gene. Since a crosstalk exists between the secretory pathway and the early endocytic route in the early MVBs/trans-Golgi network [62], we therefore tested whether some genes associated with both secretory and endocytic trafficking were influenced by dysfunction of the AAA ATPase LRD6-6. Indeed, the expression of gene *Os01g74180*, encoding the β-subunit of adaptor protein complex 3 (AP-3), whose homologs have been shown to be important regulators of both endocytic and secretory pathways in yeast, mammals and *Arabidopsis* [63–65], was down-regulated in *lrd6-6* plants and the Kitaake plants carrying *Lrd6-6*^E315Q^ (S20 and S21 Figs, S2 Table). The clathrin heavy chain gene (chcA) was reported to be essential for secretion of lysosomal enzymes in *Dictyostelium discoideum* [66]. In *Arabidopsis*, the *chc2* single mutant and dominant-negative CHC1 (HUB) transgenic lines were defective in bulk endocytosis and in internalization of prominent plasma membrane proteins [67]. We also found the *Os12g01390* gene, coding for the clathrin heavy chain was up-regulated in *lrd6-6* based on the RNA-seq data (S2 Table). These results indicate that MVBs-mediated vesicular trafficking is associated with the ATPase LRD6-6 and this trafficking process is dysregulated in *lrd6-6* and the Kitaake plants expressing *Lrd6-6*^E315Q^.

**Fig 8:**
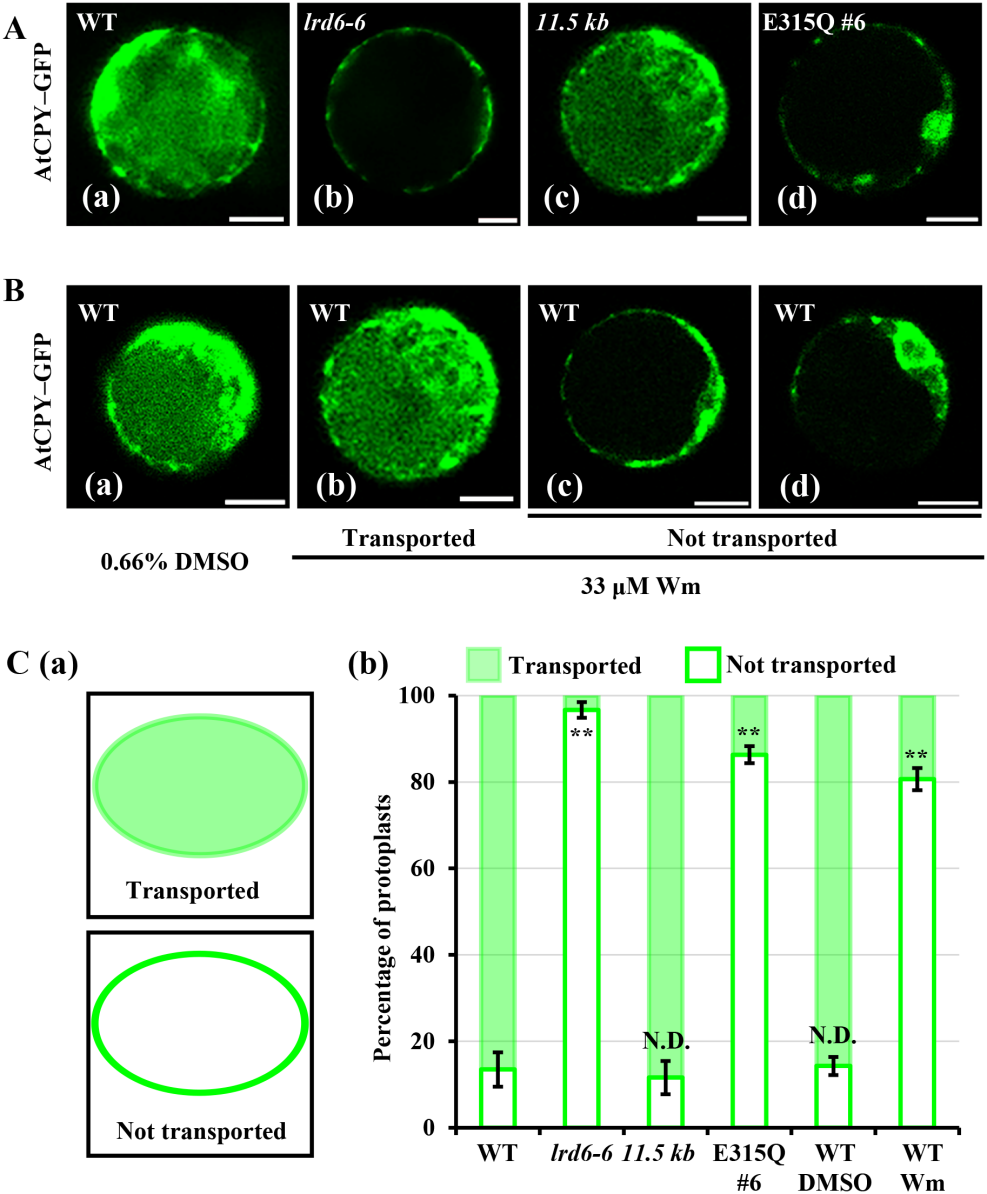
Determination on the transport of the Cargo AtCPY–GFP in Kitaake, the *lrd6-6* mutant, the *lrd6-6* plants expressing *Os06g03940-11*.*5kb* and the Kitaake plants expressing *Lrd6-6*^E315Q^. (A) The cargo protein AtCPY–GFP was transiently expressed in the protoplast cells as indicated respectively through PEG-mediated transformation. Fluorescence was determined 16 h post transformation. (B) Wortmannin treatment inhibited the transport of the cargo AtCPY–GFP to vacuole. The control (a) and the two types of AtCPY–GFP distribution in the treated protoplasts (b-d) were shown. Wortmannin was added into the incubation buffer 4 h after PEG-mediated transformation and fluorescence was determined 16 h post transformation. Bars = 10 μm. (C) The statistical results of (A) and (B) were shown. Two types of AtCPY–GFP distribution in the protoplasts were shown in (a), and statistic were performed and compared by Student’s *t*-test (**, P < = 0.01; N.D., No significantly difference), error bars were the SDs of three biology repeats, n > 80. Green color indicates GFP signal of the cargo AtCPY–GFP.

Previous studies have shown that the *Arabidopsis* soluble vacuolar Carboxypeptidase Y (AtCPY) is transported from endoplasmic reticulum (ER) to the vacuole through the early secretory pathway mediated by MVBs [68]. Dysregulation of MVBs-mediated vesicular trafficking inhibits the AtCPY–GFP transport in *Arabidopsis* [45]. To detect whether the MVBs-mediated vesicular trafficking process was dysregulated in *lrd6-6*, we transiently expressed AtCPY fused to the N-terminal GFP (AtCPY–GFP) in protoplasts prepared from Kitaake and *lrd6-6*, respectively. The GFP fluorescence signal of AtCPY–GFP in Kitaake protoplasts could be visualized in the vacuole (Fig 8Aa and 8C). By contrast, the fluorescence was prominently presented in cytoplasm in the *lrd6-6* protoplasts (Fig 8Ab and 8C), showing that the trafficking of AtCPY–GFP from ER to the vacuole was obviously inhibited in *lrd6-6*. The transport of AtCPY–GFP in the *lrd6-6* plants expressing *Os06g03940-11*.*5kb* transgene was restored to the normal level of the wild type Kitaake plants whereas inhibited in the Kitaake plants expressing *Lrd6-6*^E315Q^ (Fig 8Ac, 8Ad and 8C). These results clearly showed that the MVBs-mediated vesicular trafficking was modulated by the AAA ATPase LRD6-6. To determine whether dysfunction of MVBs could inhibit the transport of AtCPY–GFP in rice, we treated the Kitaake protoplasts transiently expressing AtCPY–GFP with wortmannin. We found that the transport of AtCPY–GFP was inhibited in most protoplasts (about 80%) treated with wortmannin (Fig 8B and 8C). This result clearly shows that transport of rice-expressed AtCPY–GFP from ER to vacuoles is also mediated by MVBs and this transport is completely inhibited in the *lrd6-6* mutant.

Taken together, our results suggest that the MVBs-mediated vesicular trafficking is altered in the *lrd6-6* mutant and the autoimmunity and spontaneous cell death of *lrd6-6* likely results from the dysregulated MVBs-mediated vesicular trafficking.

To determine whether loss-of-function of LRD6-6 affects MVBs in general, we transiently expressed RabF1/ARA6–GFP in the protoplast cells prepared from Kitaake and *lrd6-6* respectively. The result showed that localization of RabF1/ARA6–GFP in *lrd6-6* was not changed compared to that observed in Kitaake (S22 Fig). This result suggested that LRD6-6 does not affect the machinery of MVBs generally.

### Antimicrobial metabolites accumulate in the *lrd6-6* mutant

Among the DEGs identified with RNA-seq, many were associated with immunity and cell death according to GO biological function analysis. These included *PR* genes, chitinase, WRKY transcription factors, MPKs and oxidation-related genes (S3 Table). We randomly selected some of these genes and verified their differential expression between the *lrd6-6* mutant and wild type Kitaake (S23 Fig).

To explore the downstream events involved in the autoimmunity and spontaneous cell death in the *lrd6-6* mutant, we performed pathway analysis on the DEGs–many pathways were likely involved in the immunity and cell death (S4 Table). Of them, three pathways, phenylpropanoid biosynthesis, diterpenoid biosynthesis and phenylalanine, tyrosine and tryptophan biosynthesis, which contribute to innate immunity and cell death according to previous reports [69–71] were highly activated in the *lrd6-6* mutant compared with the wild type Kitaake (S24 Fig and S4 Table).

**Fig 9:**
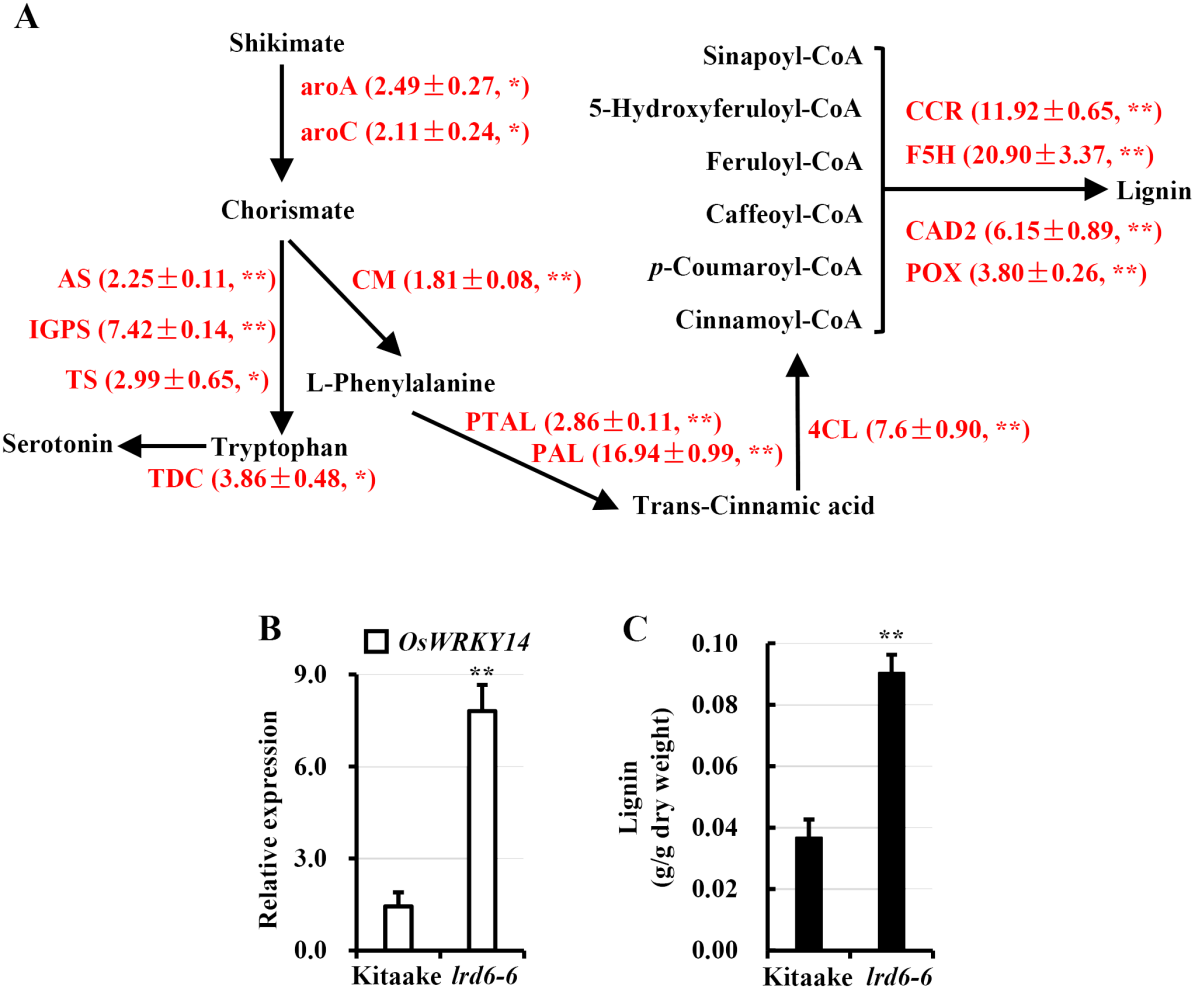
Synthesis of serotonin and lignin were greatly activated in *lrd6-6* mutant plants. (A) The shikimate-initiated synthesis pathway of serotonin and lignin. The key enzyme genes with increased expression in the *lrd6-6* mutant detected by RNA sequencing are shown in red. Data in the brackets are the gene expression fold-changed determined by qRT-PCR with gene specific primers (Fold-changed ± SD), asterisks denote statistical gene expression increases in *lrd6-6* mutant compared to Kitaake by Student’s *t*-test (*, P < = 0.05; **, P < = 0.01). (B) Comparison of the expression of *OsWRKY14*, which regulates synthesis of serotonin directly, between Kitaake and *lrd6-6*. (C) Content determination of lignin in Kitaake and *lrd6-6*. The bars represent the means of data from a representative experiment, and the error bars indicate the SEM (n = 4). Asterisks denote a significant difference between the *lrd6-6* mutant and the wild type Kitaake (Student’s *t*-test; **, P < 0.01).

**Fig 10:**
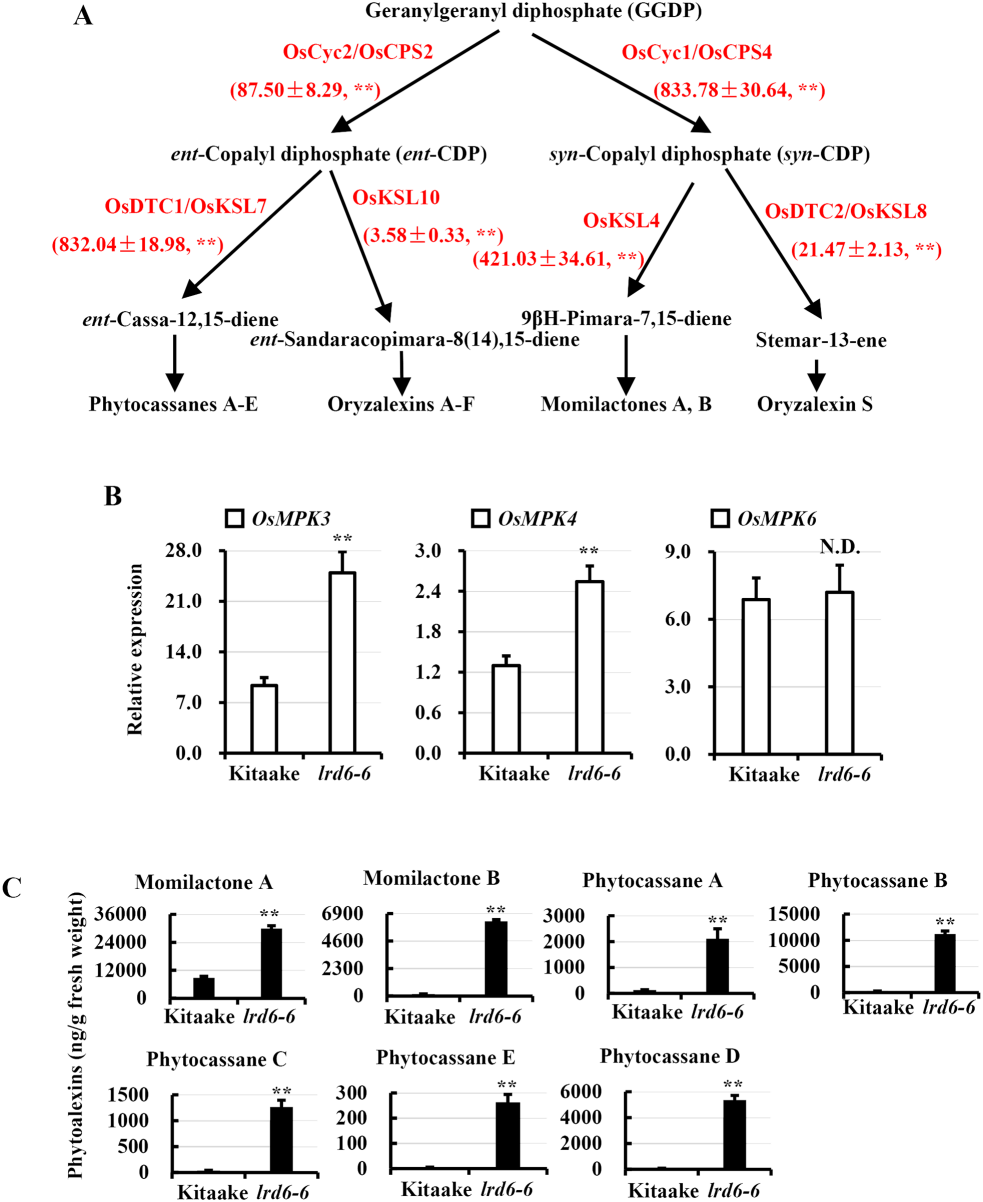
Diterpenoid phytoalexin biosynthesis is up-regulated in the *lrd6-6* mutant. (A) The geranylgeranyl diphosphate (GGDP) initiated synthesis pathway of diterpenoid phytoalexins. The key enzyme genes with increased expression detected by RNA-seq are shown in red. Data in the brackets are the gene expression fold-changed determined by qRT-PCR with gene specific primers (Fold-changed ± SD), asterisks denote statistical gene expression increases in *lrd6-6* mutant compared to Kitaake by Student’s *t*-test (**, P < = 0.01). (B) Comparison of the expressions of *OsMPK3*, *OsMPK4* and *OsMPK6*, which regulate synthesis of diterpenoid phytoalexins, between the *lrd6-6* mutant and Kitaake. The expression levels of the genes were normalized to the *Ubq5* reference gene. Error bars represent the SDs for three biology repeats and the expression differences was determined by Student’s *t*-test (**, P < = 0.01; N.D., No significantly difference). (C) Content determination of the phytoalexins as indicated in Kitaake and *lrd6-6*. The data was obtained from four biological replicates. The error bars indicate the SEMs. Asterisks denote a significant difference between the *lrd6-6* mutant and Kitaake (Student’s *t*-test; **, P < 0.01).

Gene pathway analysis also showed that the antimicrobial metabolites, including serotonin, lignin and phytoalexins might accumulate in the *lrd6-6* mutant (Figs 9A and 10A). We thus performed qRT-PCR to analyze the expression of the key enzyme genes involved in biosynthesis of these antimicrobial metabolites. Indeed, we found that expression of the genes *aroA* and *aroC*, required for biosynthesis of chorismate from shikimate [72, 73], obviously increased in the *lrd6-6* mutant (Fig 9A). Because chorismate is required for biosynthesis of both serotonin and lignin [74, 75], the increased expression of *aroA* and *aroC* would lead to accumulation of the antimicrobial metabolites, serotonin and lignin. We then found that expression of the downstream genes essential for respective biosynthesis of serotonin and lignin initiated from chorismate (for biosynthesis of serotonin: *As*, *Igps*, *Ts* and *Tdc*; for biosynthesis of lignin: *Pal*, *Ptal*, *4Cl*, *Ccr*, *F5h*, *Cad* and *Pox*) increased in the *lrd6-6* mutant (Fig 9A), supporting our notion that the antimicrobial metabolites, serotonin and lignin, would accumulate in the *lrd6-6* mutant. Then we measured the expression of the genes required for the biosynthesis of phytoalexins, including phytocassanes A–E (genes: *OsCyc2*/*OsCps2* and *OsDtc1*/*OsKsl7*), oryzalexins A–F (genes: *OsCyc2*/*OsCps2* and *OsDtc1*/*OsKsl10*), oryzalexin S (genes: *OsCyc1*/*OsCps4* and *OsDtc2*/*OsKsl8*) and momilactones A and B (genes: *OsCyc1*/*OsCps4* and *OsDtc1*/*OsKsl4*) [76] between the *lrd6-6* mutant and Kitaake (Fig 10A). These genes expressed higher in the *lrd6-6* mutant compared with Kitaake (Fig 10A). This result suggested that biosynthesis of these phytoalexins might be highly activated in the *lrd6-6* mutant. Previous reports have shown that the WRKY transcription factor OsWRKY14 is required for serotonin biosynthesis through regulating the expression of genes *TS* (*tryptophan synthase*) and *TDC* (*tryptophan decarboxylase*) [74, 77] and that the MPK genes *OsMPK3*, *OsMPK6* and *OsMPK4* regulate the biosynthesis of lignin and phytoalexins [75, 78]. When determining the expression of these genes, with the exception of *OsMPK6*, we found that the expression of all these genes was highly increased in the *lrd6-6* mutant compared with Kitaake (Figs 9B and 10B), further supporting our notion that antimicrobial metabolites accumulated in the *lrd6-6* mutant.

To confirm that these antimicrobial metabolites accumulate in *lrd6-6*, we selectively measured and compared the contents of lignin and phytoalexins between *lrd6-6* and Kitaake. The result showed that the amount of total lignin and phytoalexins, momilactones A and B, and phytocassanes A–E in *lrd6-6* were indeed largely increased compared with Kitaake (Figs 9C and 10C). Consistently, the contents of phytoalexins in the *lrd6-6* plants expressing the transgene *Os06g03940-11*.*5kb* was restored to the levels of the wild type Kitaake while highly accumulated in the transgenic Kitaake plants expressing *Lrd6-6*^E315Q^ which exhibited cell death (S25 Fig). Previous study has shown that salicylic acid (SA) biosynthesis is involved in shikimate-phenylpropanoid pathway [79], which is also involved in lignin biosynthesis activated in *lrd6-6*. Elevated levels of SA could stimulate SA-mediated defense response leading to secretion of antimicrobial compounds and increased expression of *PR* genes in plant [80]. To determine whether SA was accumulated in *lrd6-6*, we sampled the leaf of the *lrd6-6* mutant, the *lrd6-6* plant expressing the transgene *Os06g03940-11*.*5kb*, the Kitaake plant expressing *Lrd6-6*^E315Q^ and the wild type Kitaake and subjected them to total SA content determination. The result showed that there was no significant difference of SA contents among the samples (S26 Fig). Taken together, these results suggested that the enhanced immunity and cell death in *lrd6-6* results from accumulation of phytoalexins. These results also indicate that the accumulation of the antimicrobial metabolites instead of SA in the *lrd6-6* mutant likely directly inhibit the infection of pathogens such as *M*. *oryzae* and *Xoo*.

### LRD6-6 interacts with ESCRT-III components OsSNF7 and OsVPS2

**Fig 11:**
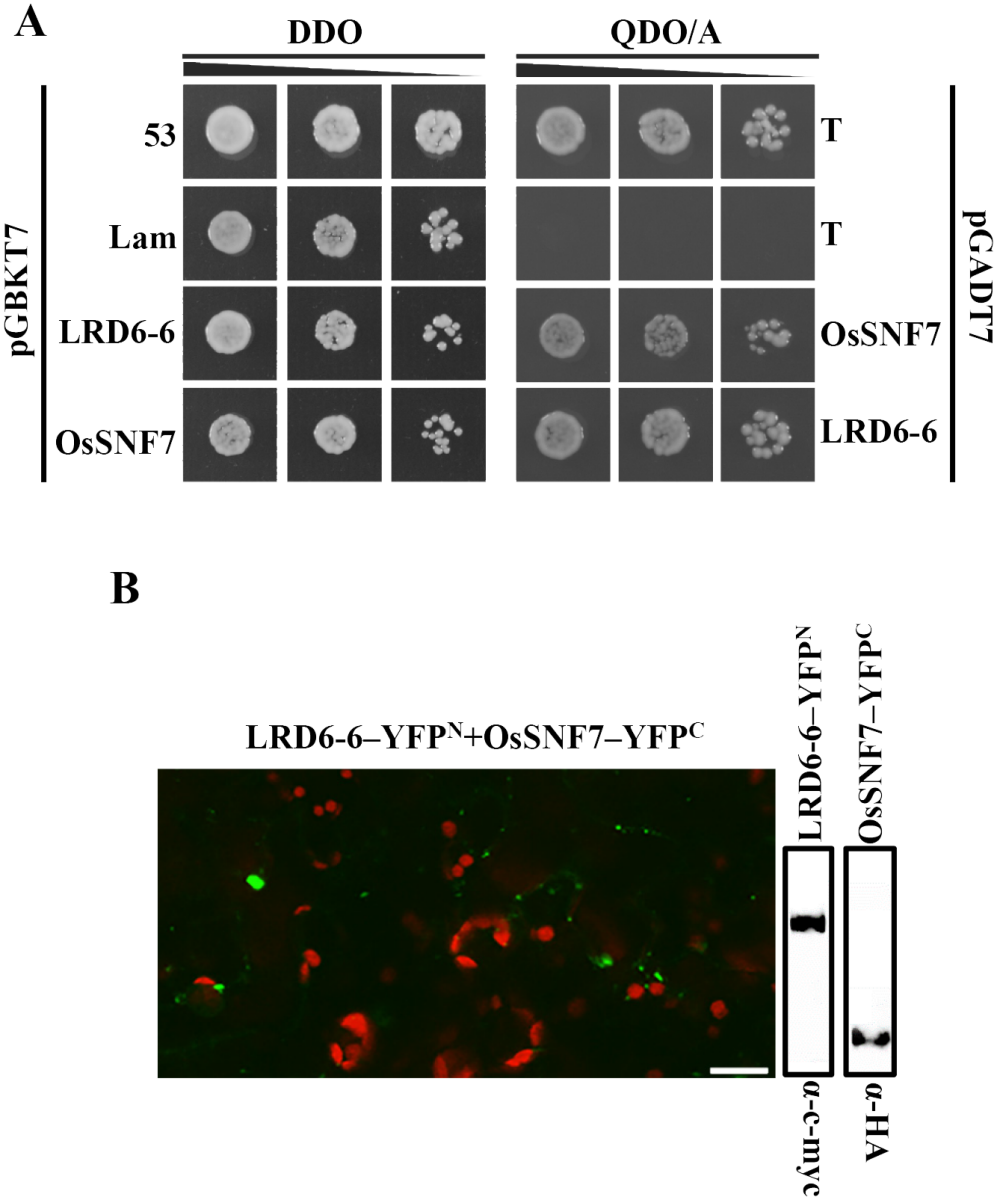
The ESCRT-III component OsSNF7 interacts with LRD6-6. (A) OsSNF7 interacts with LRD6-6 in yeast. (B) Determination of the interaction between OsSNF7 and LRD6-6 in *N*. *benthamiana*, bars = 20 μm.

To further dissect the molecular mechanism of LRD6-6 in regulation of immunity and cell death, we performed an Y2H screen using LRD6-6 as bait. The full amino acid coding sequence of *Lrd6-6* was cloned in-frame with the GAL4 DNA binding domain of the bait vector pGBKT7. The Y2H screen was conducted using a cDNA library derived from rice Nipponbare. We identified a SNF7 domain containing protein (Os06g40620) and named this protein OsSNF7 because it showed high identity with *Arabidopsis* SNF7 in amino acid sequence. To confirm its interaction with LRD6-6, we amplified the full-length CDS of *OsSnf7* and cloned it into the prey vector to obtain pGADT7-OsSNF7. The Y2H test clearly showed that full-length protein of OsSNF7 interacted with LRD6-6 (Fig 11A). We then fused OsSNF7 with GFP, LRD6-6 with RFP respectively and co-expressed them into *N*. *benthamiana* and observed their localizations. The result showed most of the green fluorescence produced by OsSNF7–GFP overlapped with the red fluorescence signals of LRD6-6–RFP (S27 Fig). This result suggests that the OsSNF7 protein also resides in MVBs and co-localizes with LRD6-6. The interaction between OsSNF7 and LRD6-6 was further confirmed by BiFC analysis in *N*. *benthamiana* through co-expressing LRD6-6–YFP^N^/OsSNF7–YFP^C^ (Fig 11B).

To test if OsSNF7 functions in immunity and cell death in plants, we knocked out (KO) *OsSnf7* in rice Kitaake using the CRISPR/CAS9 approach [81]. Three independent *OsSnf7*-KO rice lines with premature stop mutation at different residues were obtained (S28 Fig). Unexpectedly, these *OsSnf7*-KO plants, which were homozygous for their respective premature stop mutation, displayed no obvious cell death or growth defects compared with Kitaake. The expression of *PR* genes in these lines remained at the same level as wild type Kitaake (S29 Fig). These results suggested that the knock-out of *OsSnf7* did not affect the immune response or cell death in plants. We then analyzed the homologs of OsSNF7 in rice and found four proteins encoded by genes *Os12g02830*, *Os11g03060*, *Os09g09480* and *Os07g30830* that also shared high identities with OsSNF7 (S30A Fig). We then cloned three of these homologs and tested their interactions with LRD6-6. The result showed that at least the protein encoded by *Os12g02830* could interact with LRD6-6 (S30B Fig), suggesting that *Os12g02830* likely functions redundantly with OsSNF7. Thus, to elucidate the role of OsSNF7 and its homologs in immunity and cell death in rice, we may need to develop double or multiple knock-out mutants of these genes in future studies.

Another core component of the ESCRT-III complex, VPS2, is also MVBs-localized and interacts with the AAA ATPase AtSKD1 in *Arabidopsis* [82, 83]. Three VPS2 homologs, encoded by *Os03g43860*, *Os07g13270* and *Os11g47710*, respectively, were predicted in rice, based on alignment analysis of protein sequences [84]. To determine if these rice VPS2s interact with LRD6-6, we cloned full length CDSs of these genes into the vector pGADT7 to obtain pGADT7-*Os03g43860*, pGADT7-*Os07g13270*, and pGADT7-*Os11g47710*, respectively, and used them for Y2H tests. The result showed that the VPS2 homologous protein (we named it OsVPS2) encoded by *Os03g43860* interacted with LRD6-6 (S31A Fig). BiFC assay showed that the *N*. *benthamiana* cells co-expressing OsVPS2 and LRD6-6 displayed punctate fluorescence signals with a similar pattern as LRD6-6 (S31B Fig) which further confirms the interaction between LRD6-6 and OsVPS2. These results also confirm that LRD6-6 localizes in MVBs.

## Discussion

### The AAA ATPase LRD6-6 inhibits immunity and cell death in rice

Rice *lrd* mutants usually exhibit immunity-mediated cell death [48]. The *lrd6-6* mutant possessed highly activated biosynthesis of antimicrobial metabolites with accumulated lignin and phytoalexins, which have been proven to fight against pathogens in rice and other plants [71, 80, 85, 86] (Figs 9, 10, S24 and S25). Consistently, like most *lrd* mutants, *lrd6-6* displayed accumulation of hydrogen peroxide and spontaneous cell death (Figs 1B and S4) which are usually associated with the immune response. Indeed, the immunity was enhanced in the *lrd6-6* mutant as the expression of the genes associated with immunity, including the *PR*, chitinase, WRKY transcription factors, MPKs and oxidation-related genes, were remarkably upregulated compared with wild type Kitaake (Figs 1C and S23, S3 Table). The enhanced immunity and cell death in the *lrd6-6* mutant thus led to increased resistance to the pathogens *M*. *oryzae* and *Xoo* (Figs 1D, 1E and S5). Further analyses identified a novel AAA ATPase LRD6-6, whose disruption resulted in the *lrd* phenotype in the *lrd6-6* mutant (Figs 2 and S9). Thus, the ATPase LRD6-6 negatively regulates immunity and cell death in rice. A very recent study reported cloning of the *Lmr* gene, the same gene as *Lrd6-6*, which harbors a G to A base substitution resulting in a premature translation termination in the *lmr* mutant [51]. The *lmr* mutant also showed cell death and displayed enhanced disease resistance to pathogens like the *lrd* mutant. However, this previous study did not characterize this protein biochemically or elucidate the underlying regulatory mechanism [51].

LRD6-6 contained Walker A, Walker B and SRH motifs, which have been characterized in typical AAA ATPases [34–36] (S15 Fig). High identities in amino acid sequences shared by LRD6-6 with the previously characterized AAA ATPases, SKD1 from human [57], Vps4p from yeast [58], mcSKD1 from ice plant [87] and At-KD1 from *Arabidopsis* [38] (S15 Fig), and the ATPase activity possessed by the recombinant LRD6-6 protein purified from *E*. *coli* further showed that LRD6-6 is an active AAA ATPase (Fig 4). Unlike the wild type LRD6-6, none of its ATPase catalytically inactive or impaired variants—LRD6-6^K261A^, LRD6-6^E315Q^ or LRD6-6^R372E^—could inhibit the immunity-mediated cell death in the *Lrd6-6-*disrupted *lrd6-6* plants (Fig 6), indicating that the ATPase activity was required for LRD6-6 to function. Interestingly, LRD6-6 was able to homo-dimerize in both yeast and plants (Fig 5). The ATPase catalytically inactive variant LRD6-6 ^E315Q^ was able to exert a dominant-negative effect through dimerization (Fig 7). This E (Glu) residue is widely conserved among AAA ATPases of the SKD1 subfamily, including human SKD1, yeast Vps4p, ice plant mcSKD1 and *Arabidopsis* AtSKD1, all of which are highly homologous to LRD6-6 (S15 Fig). The equivalent EQ mutants, with the conserved residue E (Glu) replaced by Q (Gln), SKD1^E235Q^ [57], Vps4p^E233Q^ [58], mcSKD1^E231Q^ [88] and AtSKD1^E232Q^ [38], are also able to abolish their respective ATPase activity and exert their regulatory roles dominant-negatively. Thus, these AAA ATPases share conserved biochemical characteristics and may function similarly biologically.

Previous studies have isolated about 14 genes responsible for *lrd* phenotypes and exhibit immunity-mediated cell death. Of them, only SPL28 is associated with protein trafficking. However, the Golgi apparatus-localization suggests that SPL28 may specifically be involved in the post-Golgi trafficking process [26]. Thus none of these proteins are associated with ATPase, MVBs-mediated vesicular trafficking as is LRD6-6. Together, these suggest that the molecular regulation of immunity and cell death in these *lrd* mutants are very complicated and the regulatory machinery in the *lrd6-6* mutant differs from those in other *lrd* mutants characterized previously.

### The LRD6-6 protein targets MVBs, and the MVBs-mediated vesicular trafficking is associated with rice immunity and cell death

Many AAA ATPases belong to the same subfamily as LRD6-6, including the mammal SKD1 [57], yeast Vps4p [58], ice plant mcSKD1 [87] and *Arabidopsis* AtSKD1 [38], which spread mainly on MVBs and are required for MVBs biogenesis, and its mediated vesicular trafficking [60]. In the regulation of MVBs biogenesis, the AAA ATPase of this subfamily is recruited to the MVBs membrane by ESCRT-III subunits. Then, the AAA ATPase provides the energy to disassociate the ESCRT-III complex from the membrane, and this has been characterized as the last essential step of MVBs biogenesis [60]. When the AAA ATPase is mutated or its function is rendered dominant-negative by interaction with its catalytically inactive EQ mutant, both the MVBs biogenesis and its mediated vesicular trafficking are largely blocked [60]. This can even be lethal in *Arabidopsis* [38, 45], and suggests the conserved roles of these AAA ATPases in diverse species [36]. A recent study reported that LMR, encoded by the same gene *Lrd6-6*, localizes in chloroplasts for its function [51]. However, our study revealed that the LRD6-6 protein spreads on MVBs (Fig 3), which is in agreement with the MVBs-localization of the AAA ATPases from this family [38, 57, 58, 88]. Firstly, the protein LRD6-6 fused with GFP and RFP on its C-terminus and with YFP on its N-terminus all displayed a punctate distribution and co-localized with the MVBs-localized marker protein RabF1/ARA6 but did not overlap with the chlorophyll auto-fluorescence (Figs 3 and S14). Based on our results, the subcellular localization pattern of LRD6-6 is similar to its *Arabidopsis* homolog AtSKD1, previously shown to be MVBs-localized [38]. Secondary, LRD6-6 co-localized and interacted with ESCRT-III components OsSNF7 and OsVPS2 (Figs 11, S30 and S31), which are respectively homologous to the MVBs-localized AtSNF7 and AtVPS2 from *Arabidopsis* [82, 84]. Taken all together, we thus conclude that LRD6-6 mainly localizes on MVBs.

The MVBs-localization suggests that the LRD6-6 AAA ATPase is associated with MVBs-mediated vesicular trafficking in rice. Indeed, although this trafficking machinery is not generally affected (S22 Fig), it is dysregulated in the *lrd6-6* mutant as revealed by whole transcriptome expression analyses and the inhibited trafficking of the soluble vacuolar cargo AtCPY from ER to vacuoles (Figs 8, S20 and S21, S2 Table). Both the genes that likely encoding components of secretory and endocytic trafficking are co-regulated. For example, the AP-3β-coding gene *Os01g74180* was down-regulated; the clathrin heavy chain coding gene *Os12g01390* was up-regulated in both *lrd6-6* and Kitaake plants expressing *Lrd6-6*^E315Q^ (S20 and S21 Figs, S2 Table). It is thus likely that both these two trafficking pathways are dysregulated in plants with loss of function of the AAA ATPase. Thus, our study demonstrates the essential roles of the AAA ATPase LRD6-6 in MVBs-mediated vesicular trafficking similar to SKD1 [57] and its homologous proteins AtSKD1 from *Arabidopsis* [38, 45], VPS4p from yeast [58]. Our study also supports the notion that the AAA ATPases of the SKD1 subfamily are conserved in function in diverse species [60]. Importantly, our study reveals that the AAA ATPase LRD6-6 inhibits the immune response, suggesting that the LRD6-6-mediated modulation of immunity and cell death is associated with MVBs-mediated vesicular trafficking in rice, which is not reported previously for SKD1 or any other homologous proteins. Although the dysregulated MVBs-mediated vesicular trafficking may cause many biological defects, such as impeded cell proliferation, adhesion and drug resistance in humans [41], there are few reports concerning their regulation of immunity and cell death. Previous studies have implied that cell death may occur in plants with AtSKD1 knocked out or a catalytically inactive variant dominant-negative mutant AtSKD1^E232Q^ in *Arabidopsis* [38, 45]. However, no direct evidence was provided from their studies because AtSKD1 knock-out or expression of AtSKD1^E232Q^ is lethal to plants [38, 45]. Interestingly, the rice plants with disrupted LRD6-6 or dominant-negatively regulated by the catalytically inactive variant LRD6-6^E315Q^ still grow well, with the only visible defect of spontaneous cell death phenotype (Figs 1A and 7B). Thus, our discovery that the AAA ATPase LRD6-6 is essential for MVBs-mediated vesicular trafficking provides a valuable way to study its regulation on immunity and cell death. Of great importance, if introducing the catalytically inactive variant LRD6-6^E315Q^ with dominant-negative effect engineered under the pathogen-inducible promoter into rice plant, we may obtain the rice with enhanced disease resistance without affecting rice yield.

MVBs-mediated vesicular trafficking has attracted much attention because of its role observed in immunity recently [31, 32]. The animal pattern recognition receptor TLR4 (toll-like receptor 4) mediates perception of bacterial-derived lipopolysaccharides, and undergoes internalization upon activation with its cognate ligand through MVBs-mediated vesicular trafficking [89]. In *Arabidopsis*, a transmembrane leucine-rich repeat receptor kinase FLS2 that recognizes bacterial flagellin, similarly exhibits ligand-stimulated endocytosis [46, 89]. The trans-Golgi network/early endosomes component KEG is reported to play a role in plant immunity by regulation of intracellular trafficking processes, and the secretion of apoplastic defense proteins [90]. VPS35B in *Arabidopsis* is part of the retromer complex, which functions in endosomal protein sorting and vesicular trafficking, contributing to TIR-NB-LRR and CC-NB-LRR protein-mediated autoimmunity and HR cell death [91]. However, it remains unclear how these immune proteins are transported through MVBs-mediated vesicular trafficking. In rice, trafficking is essential for the OsCEBiP/OsCERK1-OsRacGRF1-OsRac1 module to regulate immunity [92, 93], while little is known about the regulation of MVBs-mediated vesicular trafficking on immunity. Our study uncovered that the inhibitory regulation of AAA ATPase LRD6-6 on MVBs-mediated vesicular trafficking may be associated with plant immunity and cell death. This discovery indicates that certain immune response-associated protein(s), including OsCEBiP in PTI, NBS-LRR proteins in ETI, and important regulators downstream, may not be sorted or transported properly due to dysregulated MVBs-mediated vesicular trafficking. The disordered sorting or failure in the transport of these immune response-associated proteins may then activate the immune response without pathogen infection and results in spontaneous cell death in rice. Alternatively, it is also likely that the LRD6-6 mediated-MVBs trafficking potentially is guarded by certain NBS-LRR proteins and constitutes a downstream component of the ETI pathway. The dysfunction of this process may bypass the activation of NBS-LRR and trigger the NBS-LRR-mediated ETI response, resulting in spontaneous cell death and enhanced disease resistance similarly as reviewed previously [94]. In addition, the MVBs pathway is positively regulated by pathogen responsive MPK3/6 through phosphorylation of LIP5, an ESCRT component [46]. As MPK3/6 is part of PTI and ETI responses, these results seem to support the notion that regulation of MVBs trafficking is part of the ETI/PTI pathways. It is unclear how and where serotonin, lignin, and phytoalexins accumulate in the *lrd6-6* mutant. It is possible that the blockage of transport of these antimicrobial compounds to vacuoles prevents their turnover in vacuoles and results in buildups in other compartments of the cell. Our results suggest MVBs-mediated trafficking may be essential for accurate delivery of these antimicrobial compounds.

**Fig 12:**
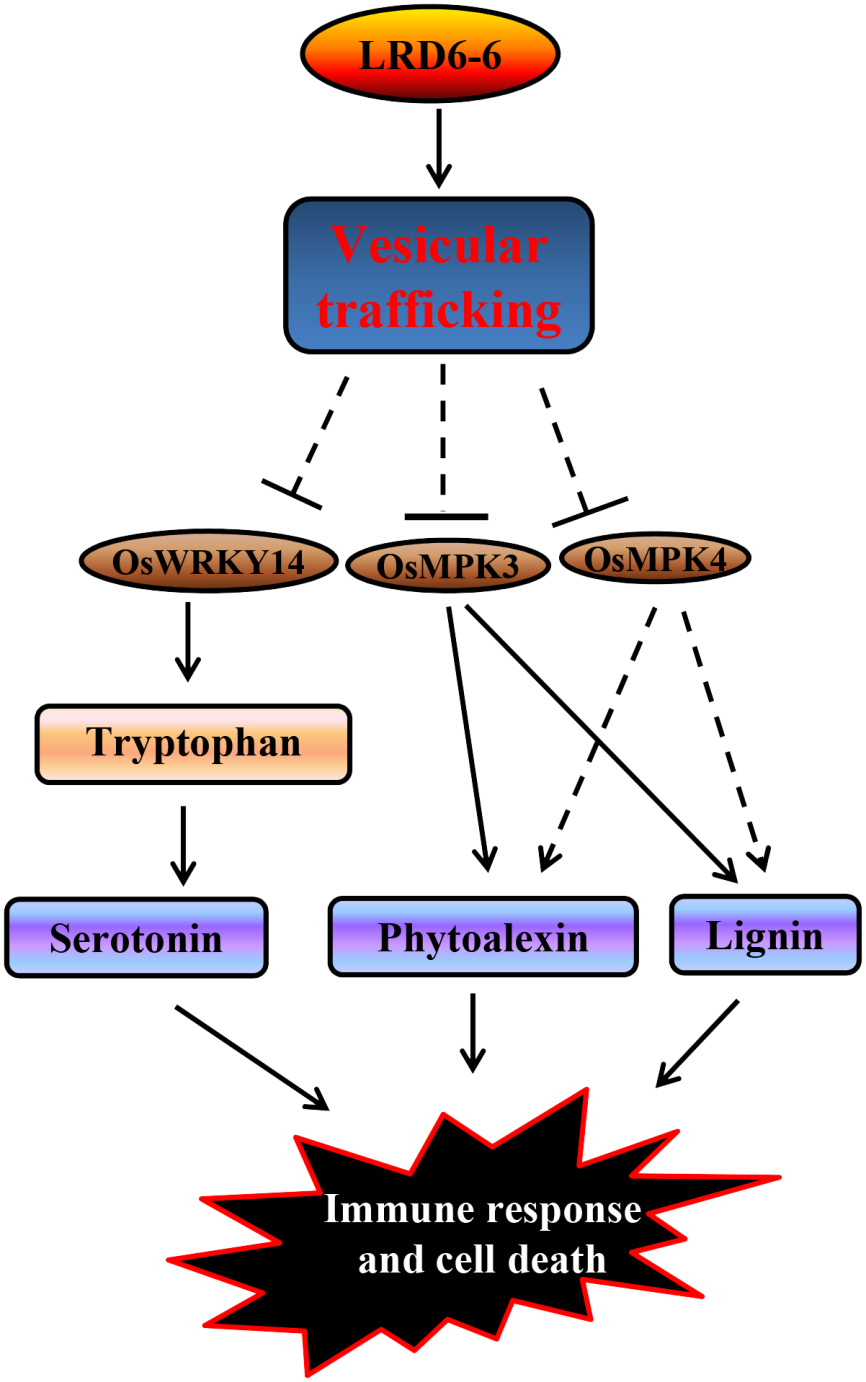
Predicted mode of *Lrd6-6* in regulation of innate immunity and cell death in rice. LRD6-6 regulates the MVBs-mediated vesicular trafficking thus inhibits the expression of genes involved in immunity such as *OsWRKYK14*, *OsMPK3* and *OsMPK4*, and then represses the accumulation of antimicrobial metabolites and prevents activation of *PR* genes to avoid activation of immunity response and cell death in rice.

Previous studies have reported that other AAA ATPases of different subfamilies to LRD6-6 are also associated with immunity in mammals and plants. The human AAA ATPase p97/VCP regulates antiviral immunity through binding directly to multi-ubiquitin chains and unfolding ubiquitin-fusion degradation substrates, such as the larger substrate adenovirus particle [39]. Overexpression of the mitochondrial outer membrane-localized AAA ATPase AtOM66 can constitutively induce salicylic acid-related defense response and cell death in *Arabidopsis* [44]. The tobacco AAA ATPase NtAAA1 inhibits innate immunity by regulating ethylene- and salicylic acid-mediated defense response through interaction with a small GTPase, NtARF [42, 43]. However, our present study reveals that dysregulation of MVBs-mediated vesicular trafficking by disruption of the AAA ATPase LRD6-6 results in accumulation of antimicrobial metabolites which then leads to activation of immunity and cell death in rice (Fig 12). This differs from the molecular regulation of immunity mediated by those AAA ATPases reported previously and defines a novel regulatory machinery of immunity in plant. Thus, our discovery provides novel insights into immunity regulated by the AAA ATPase LRD6-6 likely through MVBs-mediated vesicular trafficking in rice and possibly other species.

## Materials and Methods

### Plant materials and growth conditions

The *lrd6-6* mutant was obtained from the tissue cultured rice, Kitaake. The lesion spot appeared about 15 days after sowing. Husked seeds of Kitaake and the *lrd6-6* mutant were sterilized in 30% bleach for 30 min followed by rinsing three times with sterile ddH_2_O. To investigate whether the lesion spot phenotype occurs under sterile conditions, the sterilized seeds were germinated on ½ Murashige and Skoog (MS) medium in SoLo cup and incubated in growth chamber until presence of lesion spots on leaves. For other phenotypic characterizations and map-based cloning, the plants were grown in the fields at Sichuan Agricultural University in Wenjiang, Chengdu, or Lingshui, Hainan, China.

### Shading treatment

The leaves of both the *lrd6-6* mutant and Kitaake plants were shaded by bandaging with silver paper before initiation of lesion spot in *lrd6-6* until clear presence of lesion spots on the part of leaf without bandage.

### Chlorophyll content measurement

The leaves from the *lrd6-6* mutant and Kitaake plants were collected at three days before and after lesions appearance, respectively. Samples with 200 mg leaf tissue of each were soaked in 30 ml 80% acetone for 48 h in dark until the disks became colorless. Chlorophyll concentration was measured with four experimental repeats following the method as described previously [95].

### Histochemical analysis

Trypan blue staining assay was performed on fresh leaves following the method as described previously [96]. In brief, samples were submerged in lactic acid-phenol-trypan blue solution (2.5 mg/ml trypan blue, 25% (w/v) lactic acid, 23% water-saturated phenol and 25% glycerol in H_2_O) and were boiled in water for 2 min, then de-stained with solution containing 30% (w/v) chloral hydrate for 3 days with multiple exchanges of the solution. After distained completely, the samples were then equilibrated with 50% glycerol for five hours followed by photo-picture taken. Detection of H_2_O_2_ accumulation was carried out using DAB staining method as described previously [97]. Briefly, leaf samples were immersed in 1 mg/ml DAB containing10 mM MES (pH 6.5) for 12 h in the dark at 30°C. Then the leaf samples were transferred to solution containing 90% ethanol and 10% glycerol at 90°C until chlorophyll was completely removed. Cleared leaves were examined and photographed using an Olympus anatomical lens.

### Electron microscopy

The samples were collected from the leaves of the *lrd6-6* mutant and Kitaake plants before and after appearance of lesion spot in *lrd6-6*. The samples were then prefixed with a mixture solution of 3% glutaraldehyde. Subsequently, post-fixed in 1% osmium tetroxide, dehydrated in series acetone, infiltrated in Epox 812 for 4 hours, and embedded [98]. The sections were stained with methylene blue and ultrathin sections were cut with diamond knife, stained with uranyl acetate and lead citrate. Sections were examined under a transmission electron microscope (TEM; HITACHI, H-600IV, Japan).

### RNA isolation and qRT-PCR

The mRNA samples were extracted using TRIzol (Invitrogen, Carlsbad, CA, USA) following the procedures as described by the manufacturer. The mRNA was treated with DNase I according to the manufacturer’s instructions (Invitrogen, Carlsbad, CA, USA) and was subject to reverse transcription to synthesize first-strand cDNA. Oligo (dT) was used as primer and SuperScript II (Invitrogen, Carlsbad, CA, USA) was used as reverse transcription enzyme.

The qRT-PCR was conducted using a Bio-Rad CFX96 Real-Time System coupled to a C1000 Thermal Cycler (Bio-Rad, Hercules, CA, USA). The reference gene *Ubiquitin 5* (*Ubq5*) [99] was used as control for qRT-PCR experiments. The sequences of the primers were listed in S6 Table.

### Determination on the disease resistance of the *lrd6-6* mutant to rice blast and bacterial blight diseases

Ten-days-old seedlings of Kitaake and the *lrd6-6* mutant were used for inoculations with *Magnaporthe oryzae* (*M*. *oryzae*). The *M*. *oryzae* strains, ZB25, Zhong1 and ZE-1, which are compatible with Kitaake, were used for inoculation. Spore concentration was adjusted to 2×10^5^ spores/ml with a hemacytometer before spraying [100]. The disease lesion length and the sum of lesions were acquired at day seven after inoculation.

The *Xoo* strains, P2, P4, P5, P6 and Xoo-4, which are compatible with Kitaake, were used for inoculation. *Xoo* bacterial suspensions with 0.5 of OD_600_ were used to inoculate by using the scissors-dip method as described previously [101]. Disease lesion lengths were determined at day 15 post inoculation. The lesion lengths and bacterial populations were determined at day 0, 7, 14 post inoculation with the strain P2.

Statistical analyses were performed using SPSS version 19.0 (Released 2010. IBM SPSS Statistics for Windows, Version 19.0. Armonk, NY: IBM Corp).

### Genetic analysis and map-based cloning of the locus *Lrd6-6*

Three F_1_s and three F_2_ populations derived from the crosses of Jodan × *lrd6-6*, *lrd6-6* × Jodan and 02428 × *lrd6-6* were respectively used for the genetic analysis. The F_2_ population derived from the cross of 02428 × *lrd6-6* was used for mapping of the gene, *lrd6-6*. Bulk segregation analysis (BSA) was first used to rapidly locate the locus of *lrd6-6* on a chromosome. For the BSA analysis, equal amount of leaf blades from six F_2_ plants with the lesion spot phenotypes or six F_2_ plants with the wild type phenotypes were collected for DNA extraction to construct the mutant and the wild type DNA pools, respectively. The physical linkage map was then constructed using additional markers nearby the locus of *lrd6-6*.

The SSR primers were synthesized according to the information from Gramene database (http://www.gramene.org/microsat). InDel markers were designed based on the alignment analyses on the reference *japonica* rice Nipponbare (http://www.rgp.dna.affrc.go.jp) and *indica* rice 93–11 (http://www.rise.genomics.org.cn) genome sequences at the target location. Primers were designed using Primer 5 software. The specificity of each primer in the rice genome was confirmed by BLAST and PCR analyses.

For analysis of the PCR product, amplified products by the primer pairs of the markers, I4-2, RM19274, RM19320, I8, were separated by 3.5% agarose gel electrophoresis in 1×TAE buffer, and visualized and photographed under UV light. The amplified products by RM8075 and RM587 were separated by 6% denaturing polyacrylamide gel electrophoresis and visualized by silver staining according to the method described previously [102].

For whole genomic resequencing, *lrd6-6* was backcrossed with Kitaake for twice and self-crossed to produce BC_2_F_2_ progenies homozygous for the locus of *lrd6-6*. Equivalent total DNA of 30 BC_2_F_2_ plants homologous for *lrd6-6* locus were pooled and sequenced at Beijing Genomics Institute (BGI, Beijing, China). For genome sequence comparison, the Kitaake genome DNA was also sequenced.

### Plasmid constructs

For RNA interference (RNAi) construct, a unique cDNA fragment of *Os06g03940* from 557 bp to 1002 bp was amplified and put into the pANDA vector [103] to create RNAi construct, pANDA-*Os06g03940Ri*, using LR recombination enzyme (Invitrogen, Carlsbad, CA, USA).

For genetic complement construct, the genomic DNA fragment of 11.5 kb containing the native promoter (1960 bp upstream of ATG), entire coding region of *LOC_Os06g03940* (*Os06g03940*) and a fragment with 2295 bp downstream of TAG, was cloned into the binary vector pCactN-XG following the steps: I, Four DNA fragments covering the 11.5 kb genome sequence from Nipponbare genome were amplified with KOD DNA polymerase (Toyobo, Osaka, Japan) using the primers, lrd6-6cp-P1, lrd6-6cp-P2, lrd6-6cp-P3, lrd6-6cp-P4, listed in S5 Table. These fragments were then cloned into the vector p*EASY* Blunt Simple (TransGen Biotech, Beijing, China) to create pSimple-CP1, CP2, CP3, CP4 respectively followed by sequencing verification (Sangon Biotech, Shanghai, China). II, the DNA fragment of CP4 was released from pSimple-CP4 under digestion with the enzymes, *Nde*I and *Sal*I, and put into pSimple-CP3 pre-digested by the same enzymes to generate pSimple-CP3-CP4. III, the DNA fragment of CP1 was cut from pSimle-CP1 with *Bam*HI and *Xho*I, and put into pSimple-CP2 predigested to create pSimple-CP1-CP2. IV, the DNA fragment of CP1-CP2 was digested from pSimle-CP1-CP2 with the enzymes, *Bam*HI and *Sma*I, and put into TSK108 vector predigested to get TSK108-CP1-CP2. V, the DNA fragment of CP3-CP4 was released from pSimle-CP3-CP4 by cutting with *Sma*I and *Sal*I and cloned into TSK108-CP1-CP2 to generate an intermediate vector TSK108-CP1-CP2-CP3-CP4 (TSK108-*Os06g03940*-*11*.*5kb*). VI, the DNA fragment of *Os06g03940*-*11*.*5kb* was then cut from TSK108-*Os06g03940*-*11*.*5kb* with *Bam*HI and *Sal*I and cloned into the predigested pCactN-XG to get the destination construct pCacTN-XG-*Os06g03940*-*11*.*5*kb. The construct pCacTN-XG-*Os06g03940*-*11*.*5kb* was verified by sequencing before used for transformation (Sangon Biotech, Shanghai, China). For other constructs, the intended segments were cloned into the vector p*EASY* Blunt Simple and then sub-cloned into the destination vectors. All the constructs information and the primers used were listed in S5 Table.

### Rice genetic transformation

The *Agrobacterium*-mediated transformation was used for rice genetic transformation according to the method described previously [104]. The transgenic plants obtained were selected with Hygromycin or G418 during regeneration. The positive transgenic plants were then verified by PCR-based genotyping using the primer pairs, specific for *hygromycin* gene or *Neomycin phosphotransferase II* gene (*NPT II*) selected with G418, respectively.

### Subcellular localization

For subcellular localization in *Nicotiana benthamiana* (*N*. *benthamiana*) and onion epidermal cells, the plasmid DNA of p*35S*:*Lrd6-6*–*GFP* was introduced into *N*. *benthamiana* leaf by agroinfiltration [105] and onion epidermal cells using a bombardment-mediated gene transformation [106], respectively. The *N*. *benthamiana* leaf and onion epidermal cells were transformed with p*35S*:*GFP* as controls. Fluorescence was examined under a confocal microscopy (NiKon A1 i90, LSCM, Japan) 36 h post transformation of *N*. *benthamiana* and 16 h post transformation of onion epidermal cells, respectively.

For subcellular localization in rice protoplasts, the plasmid, pBI221–AtCPY, was transformed into protoplasts prepared from Kitaake and *lrd6-6* mutant seedlings following the method as described previously [107]. For a control, the protoplasts were transformed with pBI221. Fluorescence was examined under a confocal microscopy (NiKon A1 i90, LSCM, Japan) 16 h after transformation.

### Wortmannin treatment

The *N*. *benthamiana* leaf expressing the RabF1/ARA6–GFP or RabF1/ARA6–RFP fusion protein was immersed into solution containing 33 μM wortmannin (Selleckchem) for 40 min and then examined under a confocal microscopy similarly as described above.

For treatment of the rice protoplasts expression AtCPY–GFP, wortmannin (33 μM) was added into the incubation buffer 4 hours after transformation and then incubated at 28°C until examination.

### Protein extraction and immuno-blot analysis

The *N*.*benthamiana* leaves expressing the interested proteins were respectively harvested 48 hours post transformation. Protein extraction and immunoblotting analysis with corresponding antibodies as indicated were performed according to the method described previously [105].

For determination of the protein express in yeast, the total protein extract was prepared and analyzed with anti-Myc and anti-HA antibodies, separately, following the Yeast Protocols Handbook from Clonetech (Otsu, Shiga, Japan).

### Site directed mutagenesis of *Lrd6-6*

To obtain the mutants, K261A, E315Q and R372E of LRD6-6, the full-length coding sequence of *Lrd6-6* was cloned into the vector p*EASY* Blunt Simple. Mutagenesis was then performed with the specific primers (Listed in S5 Table) using QuikChange site-directed mutagenesis kit following the manual (Stratagene, La Jolla, California, USA) and the intermediate cloning and final constructs harboring the desired mutation sites were verified by sequencing.

### Protein expression, purification and ATPase assay

The truncated LRD6-6 (AAs: 125–487, covering the ATPase domain) and its mutation versions were cloned into the pET28a vector (Novagen), and expressed in the *E*.*coli* strain BL21. Bacteria contain the plasmids were grown in Luria-Bertani (LB) medium containing 100 μg/ml and kanamycin at 37°C to OD600 = 0.6, induced by addition of isopropyl β-D-1-thiogalactopyranoside (IPTG) to final concentration of 1 mM and incubated at 28°C for 6 hours respectively. Cells were pelleted by centrifugation, re-suspended in lysis buffer (20 mM Tris-HCl PH 7.4, 0.1 M NaCl, 10 mM imidazol) and sonicated. After the cell debris removed by centrifugation (12000 g, 10 min, 4°C), the supernatant was loaded onto a Ni-NTA-agarose column (GE Healthcare, Buckinghamshire, United Kingdom), washed with washing buffer (20 mM Tris-HCl PH 7.4, 0.1 M NaCl, 20 mM imidazol) and eluted with elution buffer (20 mM Tris-HCl PH 7.4, 0.1 M NaCl, 200 mM imidazol).

ATPase activity of LRD6-6(125–487), LRD6-6(125–487)^K261A^, LRD6-6(125–487)^E315Q^ and LRD6-6(125–487)^R372E^ were measured by the malachite green-based colorimetric method using the ATPase/GTPase activity assay kit (Sigma-Aldrich, St. Louis, MO, USA). The elution buffer was used as negative control. One unit is termed as the amount of enzyme that catalyzes the production of 1 μM of free phosphate per minute under the assay conditions.

### Yeast two-hybrid assay and library screen

Yeast two-hybrid assay was performed using the GAL4-based Matchmaker Gold Yeast Two-Hybrid System (Cat. No. 630489, Clontech, Otsu, Shiga, Japan), in which the bait protein is expressed as a fusion to the Gal4 DNA-binding domain in pGBKT7 vector and the prey protein is expressed as fusion to the Gal4 activation domain in pGADT7 vector [108, 109]. Full-length amino acids of the proteins tested were cloned in frame with the Gal4 DNA-binding or activation domain using genes specific primers to obtain the constructs (Listed in S5 Table). After sequence verified, the constructs were co-transformed by pair into the yeast stain Y2HGold via polyethylene glycol (PEG)/LiAc-based method provided by the Yeastmaker Yeast Transformation System 2 (Cat. No. 630439, Clontech). The transformed Y2HGold cells were plated on DDO media (double dropout medium: SD/–Leu/–Trp) and the cell clones were diluted for 3 gradients on the QDO/A media (quadruple dropout medium: SD/–Ade/–His/–Leu/–Trp supplemented with Aureobasidin A) for interaction test.

After confirmation that no autoactivation and toxicity exist in the Y2HGold cells co-transformed with pGBKT7-LRD6-6 and the null pGADT7 vector, yeast two hybrid screening was performed by co-transformation with the pGBKT7-LRD6-6 plasmid DNAs (10 μg) and cDNA library DNA (fused into the pGADT7 vector) (10 μg) were co-transformed into the Y2HGold cells following the procedure in the manual of Yeastmaker Yeast Transformation System 2 (Cat. No. 630439, Clontech). Plasmids of the positive clones grew on the selective QDO/A media were rescued and were respectively co-transformed with pGBKT7-LRD6-6 or the empty bait vector pGBKT7 into Y2HGold cells to validate the interactions. After verification of the interactions, the plasmid DNAs of clones were subject to sequencing.

### Bimolecular fluorescence complementation (BiFC) in *Nicotiana benthamiana*

The full-length coding sequences of *Lrd6-6*, *lrd6-6*, *Lrd6-6*^*K261A*^, *Lrd6-6*^*E315Q*^, *Lrd6-6*^*R372E*^ and *OsSnf7* without stop codon were amplified using specific primers (Listed in S5 Table) and cloned into the psPYNE and/ or psPYCE vectors through *Xba*I and *BamH*I to generate their respective YFP^N^ and/ or YFP^C^ fusion protein expressing constructs [110]. Equivolume suspensions of different *Agrobacterium* strains carrying different constructs were mixed prior to infiltration and co-infiltrated into *N*.*benthamiana* leaves following the method described previously [105]. Fluorescence was examined under a confocal microscopy (NiKon A1 i90, LSCM, Japan) 36–48 hours post transformation.

### Genome-wide transcript analysis on the *lrd6-6* mutant and Kitaake

When lesion spot initiated on leaf of the *lrd6-6* mutant, the leaf tissues from *lrd6-6* and the equivalent part of Kitaake as indicated (S18 Fig) were sampled for RNA extraction. RNA-seq was performed by CapitalBio Corporation at Beijing, China. Cuffcompare [111] was used to compare the assembled transfrags of each library to the reference annotation, and build up a non-redundant transcripts data set among the libraries. Then Cuffdiff [111] was used to identify DEGs. Transcripts data set were first compared with Kyoto Encyclopedia of Genes and Genomes database (KEGG, release 5.8) [112] using BLASTX [113] at E values < = 1e-10. A Perl script was used to retrieve KO information from blast result and then established pathway associations between transcripts and database. InterPro domains [114] were annotated by InterProScan (release 4.8) [115] and functional assignments were mapped by using GO (Gene Ontology) analysis [116]. Expression verification of the selected DEGs was conducted by using q-RT-PCR analysis. The primer pairs used were listed in S5 Table.

### Determination of the lignin, total SA and phytoalexins content in rice

The lignin content and total SA content were determined according to the acid detergent lignin method and the ultrahigh performance liquid chromatography–triple quadrupole mass spectrometry (UPLC-MS/MS) method as described previously [117, 118].

For determination of phytoalexins, tissue samples (ca. 10 mg fresh weight for each) were harvested from the *lrd6-6* mutant and Kitaake. The samples were soaked in 1 ml extraction solvent (MeOH/H_2_O, 80:20 [v/v]) and incubated at room temperature overnight. Then, 5 μL of the extract was subjected to phytoalexin measurement by LC-ESI-MS/MS. An Agilent 1200 separation module (Agilent Technologies, Palo Alto, CA, USA) equipped with a CAPCELL CORE C_18_ column (50 mm long, 2.1 mm in diameter; Shiseido, Tokyo, Japan) was used for HPLC analysis. The mobile phase consisted of 0.05% AcOH in H_2_O (solvent A), and 0.05% AcOH in MeCN (solvent B). Elution was conducted using a linear gradient from 40% to 60% solvent B over 10 min at a flow rate of 0.2 mL/min, and the eluate monitored by Agilent 6460 Triple Quadrupole mass spectrometer (Agilent). The ionization mode was electrospray. All phytoalexins were analyzed in positive ion mode. Electrospray conditions were as follows: capillary voltage, 3500 V; drying gas flow, 5 L/min nitrogen; drying gas temperature, 300°C; nebulizer pressure, 45 psi; sheath gas temperature, 350°C; and sheath gas flow, 11 L/min. The multiple reaction monitoring (MRM) mode was used in ESI-MS/MS. Phytoalexins were detected with MRM transitions of *m*/*z* 315.2/271.1 for momilactone A; *m*/*z* 331.2/269.1 for momilactone B; *m*/*z* 317.2/299.1 for phytocassanes A, D and E; *m*/*z* 335.2/317.2 for phytocassane B; *m*/*z* 319.2/301.2 for phytocassane C. Collision energy was 25 V for momilactones A and B; 15 V for phytocassanes A, D and E; 21 V for phytocassane B; 17 V for phytocassane C. Dwell time and fragmentor voltage were 200 ms and 135 V, respectively, for all phytoalexins.

### Accession numbers

Genes reported in this article can be found in the GenBank/RGAP databases under the following accession numbers: LRD6-6 (LOC_Os06g03940); RabF1/ARA6 (AT3G54840); OsSKD1 (LOC_Os01g04814); AtSKD1 (AT2G27600); ZmSKD1 (XP_008655625.1); VPS4A (NP_037377.1); VPS4B/SKD1 (NP_004860.2); VPS4p (NP_015499.1); FtsH (AAA97508.1); NSF-1 (NP_524877.1); AtCPY(At3g10410); OsSNF7 (LOC_Os06g40620); OsVPS2 (LOC_Os03g43860); CHMP4b (NP_789782.1); SHRUB (NP_610462.3); CHMP4a (NP_054888.2); scVPS32 (NP_013125.1); SNF7.1 (AT4G29160) and SNF7.2 (AT2G19830).

## Supporting Information

S1 Fig Lesion spot phenotype of the *lrd6-6* mutant under sterile conditions. Seeds of both the wild type Kitaake and the *lrd6-6* mutant were treated with 30% NaClO for 30 min followed by washing four times with autoclaved ddH_2_O. The treated seeds were then germinated in sterile ½ MS medium for two weeks before the photograph was taken. The parts of leaf showing lesion spots in the *lrd6-6* mutant and its equivalent part of the leaf from Kitaake are highlighted in the red squares.
(TIF)

S2 Fig Lesion spot phenotype of the *lrd6-6* mutant is not dependent on light. The leaves of both the *lrd6-6* mutant and the wild type Kitaake were shaded with silver paper before initiation of lesion spots in *lrd6-6* until lesion spots were clearly present on the part of the leaf without shading treatment. Photographs were respectively taken on the representative leaves of Kitaake and *lrd6-6* under native conditions (CK) and shading treatment (Shading). The result was stable both in rice field and greenhouse. Bar = 1 cm.
(TIF)

S3 Fig Comparative determination of the pigment contents between the wild type and the *lrd6-6* mutant. The pigment contents were measured in leaves from both the wild type (Kitaake) and the *lrd6-6* mutant 3 d before and 3 d after the appearance of lesion spots (represented by BL and AL, respectively). Chla: chlorophyll a, Chlb: chlorophyll b, Car: carotenoid. Error bars represent the SEM of four replicates. Asterisks denote a significant difference between the *lrd6-6* mutant and the wild type as determined by Student’s *t*-test (**, P < 0.01).
(TIF)

S4 Fig Ultrastructure of cells from Kitaake and *lrd6-6*. (A) and (B), The cell structures of the leaf part in the absence of lesion spots of Kitaake and *lrd6-6*. (C) and (D), The cell structures of the leaf part in the presence of lesion spots of *lrd6-6* and Kitaake. Magnified 6000 folds. The chloroplast (C) and nucleus (N) are marked in red.
(TIF)

S5 Fig The *lrd6-6* mutant exhibits resistance against diverse *Xoo* strains. Photographs of representative leaves and disease lesion lengths were taken at 15 d post-inoculation with four *Xoo* strains compatible with Kitaake (P4, P5, P6 and xoo-4) as indicated. Statistical analysis of the disease lesion lengths was performed on the leaves of inoculated Kitaake and *lrd6-6* (error bar, SEM, n > 8). Asterisks denote a significant difference between the *lrd6-6* mutant and the wild type as determined by Student’s *t*-test (**, P < 0.01).
(TIF)

S6 Fig Determination of whether the *lrd6-6* spontaneous cell death phenotype co-segregates with the *Hygromycin* (*Hyg*) gene. Forty individual F_2_ plants with cell death phenotypes derived from self-crossed plants heterozygous (*Lrd6-6 lrd6-6*) for *lrd6-6* locus were used for genotyping with the primer pair specific for *Hyg*. +: Plant containing *Hyg* used as positive control, WT: Kitaake lacking *Hyg* used as negative control.
(TIF)

S7 Fig Comparative analysis of the full-length cDNAs of *Os06g03940* between Kitaake and *lrd6-6*. (A) Full-length cDNAs of *Os06g03940* were amplified from Kitaake (WT) and *lrd6-6* and were separated by agarose gel. (B) Alignment on the cDNA sequences of *Os06g03940* between Kitaake and *lrd6-6*. The insertion with a repeat of 534 bp in the cDNA of *Os06g03940* in *lrd6-6* is indicated in red. (C) Alignment on the amino acid sequences encoded by *Os06g03940* between Kitaake and *lrd6-6*. The insertion with 178 AAs resulted from the 534 bp repeat of the *Os06g03940* cDNA in *lrd6-6* is indicated in red.
(TIF)

S8 Fig The sequence used for generating RNAi construct of *Os06g03940*. (A) The full-length cDNA of the gene *Os06g03940*. The coding sequence was marked in red and the UTR regions were in black. The sequence used for generating RNAi construct in this study, named as segment I (Seg I), was boxed with yellow background while the sequence used in Faklh’s paper, named as Seg II, was boxed. Features were respectively indicated below sequence. (B) The cDNA sequence identity analysis between the genes, *Os06g03940* and its closest homolog *Os01g04814*. BLAST alignment was performed by DNAMAN 6.0, the blasting hits in *Os01g04814* and the identities were respectively marked.
(TIF)

S9 Fig Co-segregation analysis of the transgenes, *Os06g03940Ri* and *Os06g03940-11*.*5kb*, respectively in the transgenic plants. (A) Photograph of representative leaves from 12 individual T_1_ plants derived from transgenic line #9. PCR-based genotyping with the primer pair specific for the *Hyg* gene was performed to determine whether the plants contained (represented by ‘+’) or lacked (represented by ‘-’) the transgene *Os06g03940Ri*. (B) Photograph of representative leaves from 12 T_1_ plants derived from one transgenic plant (line #3) carrying the transgenic fragment *Os06g03940-11*.*5kb* in *lrd6-6* genetic background. PCR-based genotyping with the primer pair specific for the *Neomycin phosphotransferase II* (*NPT II*) gene was performed to determine whether the plants contained (represented by ‘+’) or lacked (represented by ‘-’) the transgene *06g03940-11*.*5kb*.
(TIF)

S10 Fig Transcriptional expression pattern of *Lrd6-6*. Total RNA was extracted from root, stem, leaf and panicle from Kitaake rice at the two-, four- and six-leaf and mature stages. The qRT-PCR was performed to determine *Lrd6-6* expression. The expression level of *Lrd6-6* was normalized to the *Ubq5* reference gene. Error bars represent the SDs of three biology repeats.
(TIF)

S11 Fig Subcellular localization of LRD6-6–GFP. (A) Punctate pattern of LRD6-6–GFP fusion protein in *N*. *benthamiana* (a) and the detection of the expressed fusion proteins by anti-GFP (b). The constructs, *p35S*:*LRD6-6-GFP* expressing the fusion protein LRD6-6–GFP and *p35S*:*GFP* expressing GFP alone, were respectively transformed into *N*. *benthamiana* cells. Fluorescence was determined 36 h post transformation. (B) Punctate pattern of LRD6-6–GFP fusion protein in onion epidermal cells. The constructs, *p35S*:*LRD6-6–GFP* and *p35S*:*GFP*, were respectively transformed into onion epidermal cells. Fluorescence was determined 16 h post transformation.
(TIF)

S12 Fig Complementary test on the LRD6-6–GFP fusion protein in the *lrd6-6* mutant. (A) The phenotype of the *lrd6-6* mutant plants expressing the *Lrd6-6*–*GFP* transgene. Five independent transgenic *lrd6-6* lines were found to restore to the wild type phenotype. Photograph of three T_0_ lines were shown. The wild type Kitaake and the *lrd6-6* mutant were also included in the photograph. PCR-based genotyping of the *NPT II* gene was used to indicate whether the plant contained (‘+’) or lacked (‘-’) the transgenic *Lrd6-6-GFP*. Bars = 10 cm. (B) Representative leaves from the plants indicated in A. (C) Transcriptional expression level of *Lrd6-6*–*GFP* in the transgenic plants determined by qRT-PCR using the *GFP* specific primers. (D) The expression levels of the *PR* genes, *OsNPR10* and *OsPR1a*, in the plants shown in (A). The relative expression of the genes was normalized to the *Ubq5* reference gene. The error bars represent the SDs of three biological repeats and the expression differences between *lrd6-6* was determined by Student’s *t*-test (**, P < = 0.01).
(TIF)

S13 Fig Subcellular observation of the MVBs marker RabF1/ARA6 fluorescence fusion proteins in *N*. *benthamiana*. The punctate fluorescence distribution of MVBs-localized marker proteins, RabF1/ARA6-GFP (A) and RabF1/ARA6-RFP (B) turned into ring-like structures as indicated after treated by 33 μM wortmannin (Wm) for 40 min. 0.66% DMSO was used as control for wortmannin treatment. Bars = 10 μm.
(TIF)

S14 Fig Subcellular determination of the LRD6-6 fluorescence fusion proteins in *N*. *benthamiana*. The punctate fluorescence distribution of LRD6-6–GFP, LRD6-6–RFP and YFP–LRD6-6 does not co-locate with the auto-fluorescence of chlorophyll in *N*. *benthamiana*. Bars = 10 μm.
(TIF)

S15 Fig Sequence analysis of the LRD6-6 protein. (A) Phylogenetic analysis of LRD6-6 with the AAA ATPases, AtSKD1, ZmSKD1, VPS4A, VPS4B/SKD1, VPS4p, FtsH, NSF-1 and the AtSKD1 homolog OsSKD1 using Mega5.1. Bootstrap values are indicated beside each branch. (B) Structure analysis of the LRD6-6 protein. The characteristics of typical AAA ATPase (upper panel) and sequence alignment analysis on LRD6-6 with some known AAA ATPases (lower panel) are respectively shown. The key elements of Walker A and B motifs, the Pore, Sensors 1 and 2, and the second region of homology (SRH) are respectively marked in the figure. The conserved residues, K261A, E315Q and R372E in LRD6-6 are respectively indicated.
(TIF)

S16 Fig Expression of *Lrd6-6* is able to inhibit the immunity and cell death of the *lrd6-6* mutant. (A) Expression of *Lrd6-6* inhibits the spontaneous cell death of the *lrd6-6* mutant. PCR-based genotyping with the primer pair specific for the *Neomycin phosphotransferase II* (*NPT II*) gene was performed to determine whether the plants contained (represented by ‘+’) or lacked (represented by ‘-’) the transgene. (B) Expression of *Lrd6-6* compromises the expression of *PR* genes in the *lrd6-6* mutant. The expression level of *PR* genes, *OsNPR10* and *OsPR1a*, in the plants was determined by qRT-PCR. The expression was normalized to the *Ubp5* reference gene. The error bars represent the SDs of three biology repeats and the expression differences was determined by Student’s *t*-test (*, P < = 0.05; **, P < = 0.01; N.D., No significantly difference).
(TIF)

S17 Fig Expression of *Lrd6-6* does not cause cell death in Kitaake. PCR-based genotyping with the primer pair specific for the *Neomycin phosphotransferase II* (*NPT II*) gene was performed to determine whether the plants contained (represented by ‘+’) or lacked (represented by ‘-’) the transgene. The expression level of *Lrd6-6* in plants was determined by qRT-PCR. The expression was normalized to the *Ubp5* reference gene. The error bars represent the SDs of three biology repeats and the expression differences was determined by Student’s *t*-test (*, P < = 0.05; **, P < = 0.01).
(TIF)

S18 Fig Screening of differential expressed genes (DEGs) between Kitaake and *lrd6-6*. Genome-wide transcript analysis was performed on the *lrd6-6* mutant and Kitaake. The leaf part (marked in the squares) from Kitaake and the *lrd6-6* mutant were sampled when spontaneous cell death started to appear on the leaf of *lrd6-6*. A total of 1223 DEGs were obtained. Of them, 980 DEGs were up-regulated and 243 were down-regulated in *lrd6-6* [P < = 0.05, Log_2_FC (*lrd6-6*/Kitaake) > 1].
(TIF)

S19 Fig Cellular component classification of the DEGs through GO analysis. The GO terms associated with MVBs-mediated vesicular trafficking are highlighted in yellow background.
(TIF)

S20 Fig Expression verification of the DEGs associated with MVBs-mediated vesicular trafficking. (A) Analyses on three DEGs coding for MVBs-mediated vesicular trafficking pathway components. (B) Analyses on five DEGs coding the transmembrane transporters which were predicted to be transported by MVBs-mediated vesicular trafficking. Expression analyses were performed by using qRT-PCR. RNA samples were prepared from leaf samples of the *lrd6-6* mutant and Kitaake collected as used for RNA-seq analysis. The expression was normalized to the *Ubp5* reference gene. The error bars represent the SDs of three biology repeats and the expression differences was determined by Student’s *t*-test (*, P < = 0.05; **, P < = 0.01).
(TIF)

S21 Fig The expression analyses on the DEGs associated with MVBs-mediated vesicular trafficking in the *lrd6-6* plants carrying transgene *Os06g03940-11*.*5kb* and the Kitaake plants expressing *Lrd6-6*^E315Q^. (A) Analyses on three DEGs coding for MVBs-mediated vesicular trafficking pathway components. (B) Analyses on five DEGs coding the transmembrane transporters which were predicted to be transported by MVBs-mediated vesicular trafficking. Expression analyses were performed by using qRT-PCR. The expression was normalized to the *Ubp5* reference gene. The error bars represent the SDs of three biology repeats and the expression differences was determined by Student’s *t*-test (*, P < = 0.05; **, P < = 0.01).
(TIF)

S22 Fig Localization comparison on RabF1/ARA6–GFP expressed in Kitaake and *lrd6-6*. The MVBs marker protein RabF1/ARA6–GFP was transiently expressed in the protoplast cells prepared from the wild type Kitaake and the *lrd6-6* mutant, respectively, through PEG-mediated transformation. Fluorescence was determined 16 h post transformation. Arrowheads in the left panels point to some of the punctate MVBs marked by the RabF1/ARA6–GFP protein. Bars = 10 μm.
(TIF)

S23 Fig Expression verification of the DEGs involved in downstream of immune response in rice. (A) Expression comparison of the DEGs encoding the *PR* genes, *Os12g36850*, *Os12g36860* and *Os01g47070*, and the chitinase genes, *Os01g49320*, *Os10g28080* and *Os05g04690* between *lrd6-6* and Kitaake. (B) Expression comparison of the DEGs associated with ROS metabolism between the *lrd6-6* mutant and Kitaake. Expression analyses were performed by using qRT-PCR. RNA samples were prepared from leaf samples of *lrd6-6* and Kitaake collected as used for RNA-seq analysis. The expression was normalized to the *Ubp5* reference gene. The error bars represent the SDs of three biology repeats and the expression differences was determined by Student’s *t*-test (*, P < = 0.05; **, P < = 0.01).
(TIF)

S24 Fig Maps of three main pathways up-regulated in the *lrd6-6* mutant. Pathway analysis was performed on the DEGs obtained from RNA-seq data by using the KEGG database. Three main pathways, phenylpropanoid biosynthesis (A), diterpenoid biosynthesis (B) and phenylalanine, tyrosine and tryptophan biosynthesis (C), were clearly up-regulated in the *lrd6-6* mutant compared with wild type Kitaake. The enzyme genes boxed with different gradients of colors represent various levels of expression change as indicated.
(TIF)

S25 Fig Determination of phytoalexins Contents. Leaf samples of the wild type Kitaake, the *lrd6-6* mutant, the *lrd6-6* plants expressing the transgene *Os06g03940-11*.*5kb* (*lrd6-6*/*11*.*5kb*) and Kitaake plants expressing *Lrd6-6*^E315Q^ (Kitaake/E315Q #6) were respectively collected and subjected to phytoalexins determination. The data was obtained from four biological replicates. The error bars indicate the SDs. The black ** indicates significant increase between wild type Kitaake while the red ** represents the significant decrease when compared with the *lrd6-6* mutant (Student’s *t*-test; **, P < 0.01).
(TIF)

S26 Fig Total SA content determination in Kitaake, the *lrd6-6* mutant, the *lrd6-6* plants expressing the transgene *Os06g03940-11*.*5kb* and the Kitaake plants expressing *Lrd6-6*^E315Q^. Leaf samples of those materials were collected respectively and subjected to total SA determination. The error bars represent the SDs of three biology repeats and the data was compared by Student’s *t*-test to detect whether statistical differences exited. N.D. means no significantly difference.
(TIF)

S27 Fig Subcellular localization of the OsSNF7 protein. The proteins, OsSNF7–GFP and LRD6-6–RFP, were co-expressed in *N*. *benthamiana* through *Agrobacterium*-mediated transformation. Fluorescence was determined 36 h post transformation.
(TIF)

S28 Fig CRISPR/Cas9-mediated knock-out of *OsSnf7* in rice. (A) Schematic of the *OsSnf7Cas9* construct. Key elements and the sgRNA sequence for specifically targeting *OsSnf7* are respectively indicated. (B) DNA sequencing chromatograms of three *OsSnf7* knock-out (*OsSnf7*-KO) lines. The mutations, ‘G’ insertion in line *OsSnf7*-KO-#1, ‘A’ insertion in line *OsSnf7*-KO-#2 and ‘GGTG’ deletion in line *OsSnf7*-KO-#3, which lead to frameshift and premature termination in the CDS of *OsSnf7* are respectively indicated. (C) The OsSNF7 protein sequence of the wild type and the three *OsSnf7*-KO lines predicted.
(TIF)

S29 Fig Expression analysis of the *PR* genes, *OsNPR10* and *OsPR1a*, in the wild type Kitaake, the *lrd6-6* mutant and the *OsSnf7*-KO lines. Leaf samples of those lines were collected respectively and subjected to *PR* genes expression determination by using qRT-PCR. The expression was normalized to the *Ubp5* reference gene. The error bars represent the SDs of three biology repeats and the expression differences was determined by Student’s *t*-test (**, P < = 0.01; N.D., No significantly difference).
(TIF)

S30 Fig Phylogenetic analysis of OsSNF7 with the rice SNF7 homologs and previously reported SNF7 proteins in other species. (A) Full amino acid sequences of OsSNF7, CHMP4b, SHRUB, CHMP4a, scVPS32, SNF7.1, SNF7.2 and the rice SNF7 homologs were subjected to alignment and phylogenic analyses using Mega5.1. Bootstrap values are indicated beside each branch. (B) Interaction verification of the LRD6-6 protein with rice OsSNF7 homologs. Among the four homologs with high identity to OsSNF7, three genes, *Os12g02830*, *Os09g09480* and *Os07g30830*, were successfully cloned and subjected to interaction test in yeast.
(TIF)

S31 Fig Determination whether LRD6-6 interacts with the VPS2 homologs from rice. (A) Interaction test in yeast. Three rice genes predicted to code for *Arabidopsis* VPS2 homologs, *OsVps2* (*Os03g43860)*, *Os07g13270* and *Os11g47710* were cloned and were subjected yeast two hybrid assay. (B) Determination the interaction between LRD6-6 and OsVPS2 by using BiFC approach. Bar = 20 μm.
(TIF)

S1 Table Genetic analyses of the *Lrd6-6* locus. (XLSX)

S2 Table DEGs that are likely to be associated with the LRD6-6 function according to the GO cellular component classification of the RNA-seq data. (XLSX)

S3 Table DEGs which are likely to be associated with the *lrd6-6* phenotype according to the GO molecular function analysis of the RNA-seq data. (XLSX)

S4 Table Pathway annotation of the DEGs between *lrd6-6* mutant and Kitaake. (XLSX)

S5 Table Plasmid constructs and the primers used for the constructs in this study. (XLSX)

S6 Table Primers list for this study. (XLSX)
