# Supplementary figures and images for: The Multivesicular Bodies (MVBs)-Localized AAA ATPase LRD6-6 Inhibits Immunity and Cell Death Likely through Regulating MVBs-Mediated Vesicular Trafficking in Rice
Source: PLoS Genet. 2016 Sep 12;12(9):e1006311. doi: 10.1371/journal.pgen.1006311 (PMC5019419; doi:10.1371/journal.pgen.1006311)

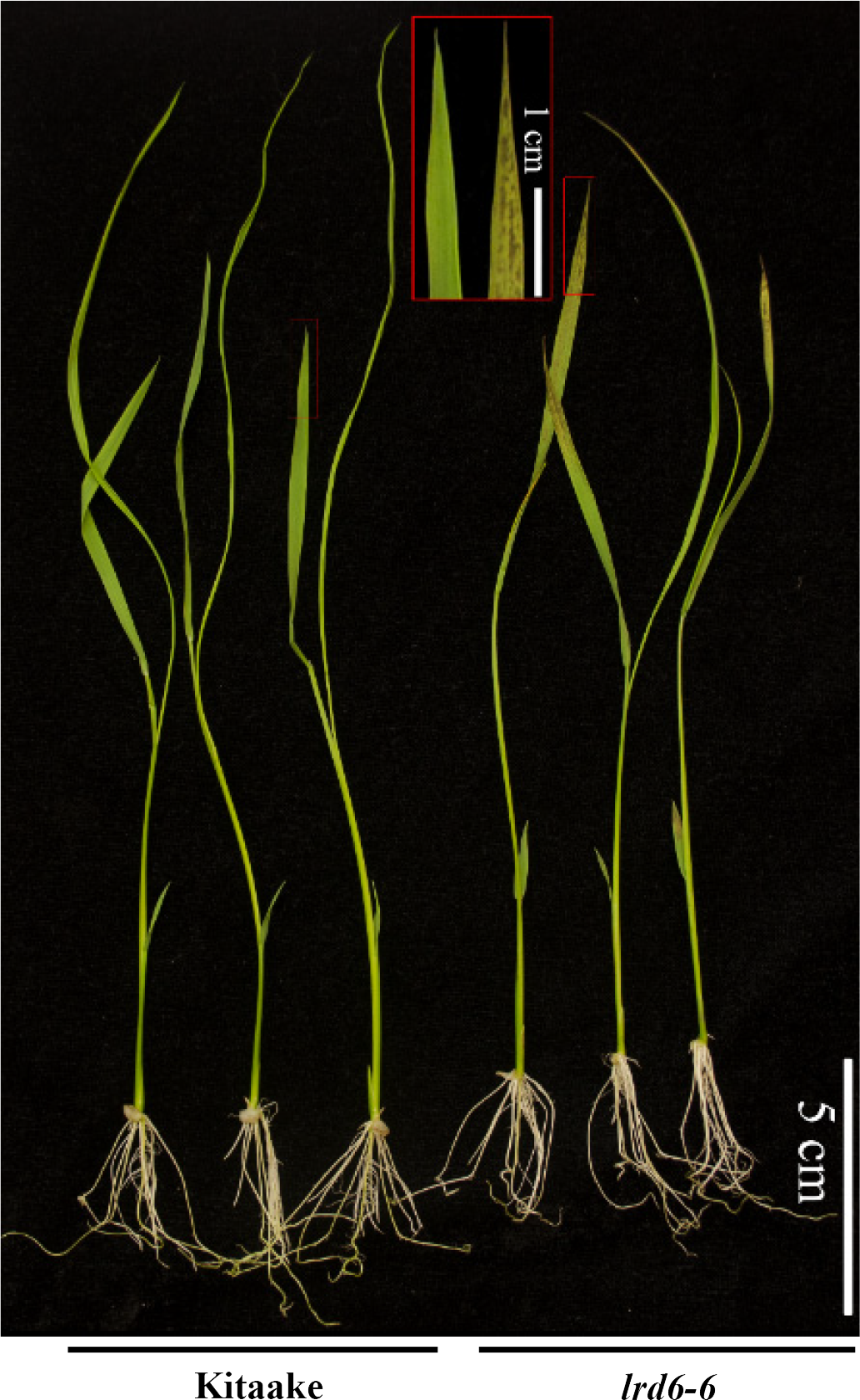

Supplement: S1 Fig — Seeds of both the wild type Kitaake and the lrd6-6 mutant were treated with 30% NaClO for 30 min followed by washing four times with autoclaved ddH2O. The treated seeds were then germinated in sterile ½ MS medium for two weeks before the photograph was taken. The parts of leaf showing lesion spots in the lrd6-6 mutant and its equivalent part of the leaf from Kitaake are highlighted in the red squares. (TIF) [file pgen.1006311.s001.tif]

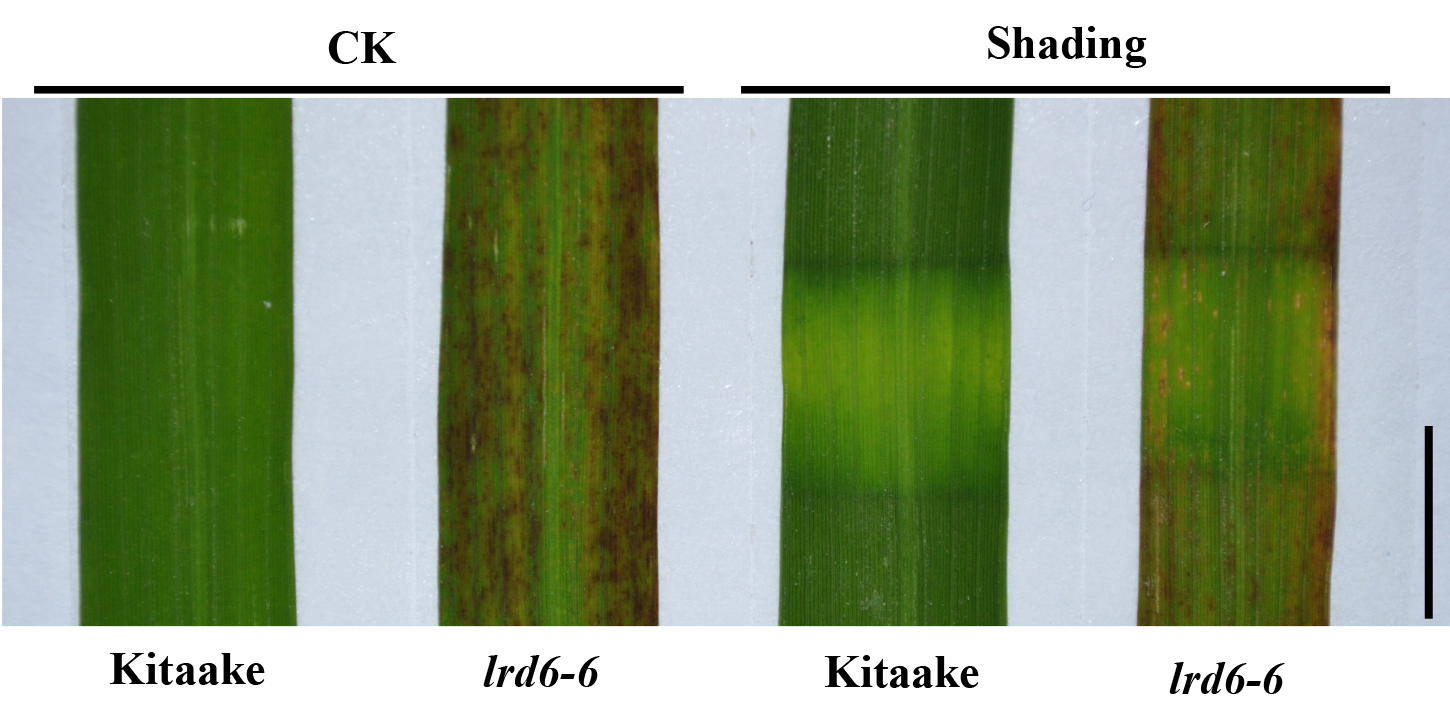

Supplement: S2 Fig — The leaves of both the lrd6-6 mutant and the wild type Kitaake were shaded with silver paper before initiation of lesion spots in lrd6-6 until lesion spots were clearly present on the part of the leaf without shading treatment. Photographs were respectively taken on the representative leaves of Kitaake and lrd6-6 under native conditions (CK) and shading treatment (Shading). The result was stable both in rice field and greenhouse. Bar = 1 cm. (TIF) [file pgen.1006311.s002.tif]

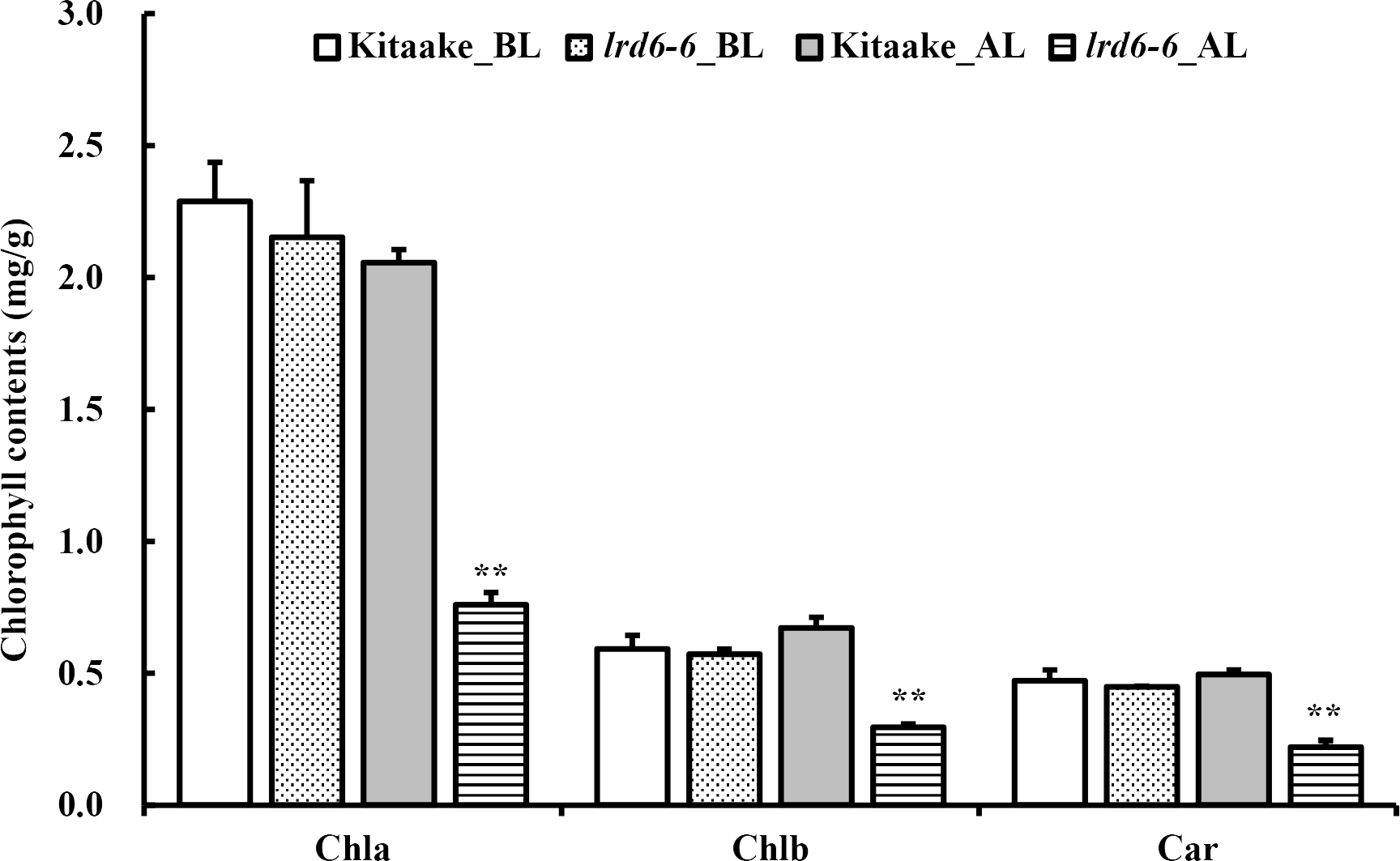

Supplement: S3 Fig — The pigment contents were measured in leaves from both the wild type (Kitaake) and the lrd6-6 mutant 3 d before and 3 d after the appearance of lesion spots (represented by BL and AL, respectively). Chla: chlorophyll a, Chlb: chlorophyll b, Car: carotenoid. Error bars represent the SEM of four replicates. Asterisks denote a significant difference between the lrd6-6 mutant and the wild type as determined by Student’s t-test (**, P < 0.01). (TIF) [file pgen.1006311.s003.tif]

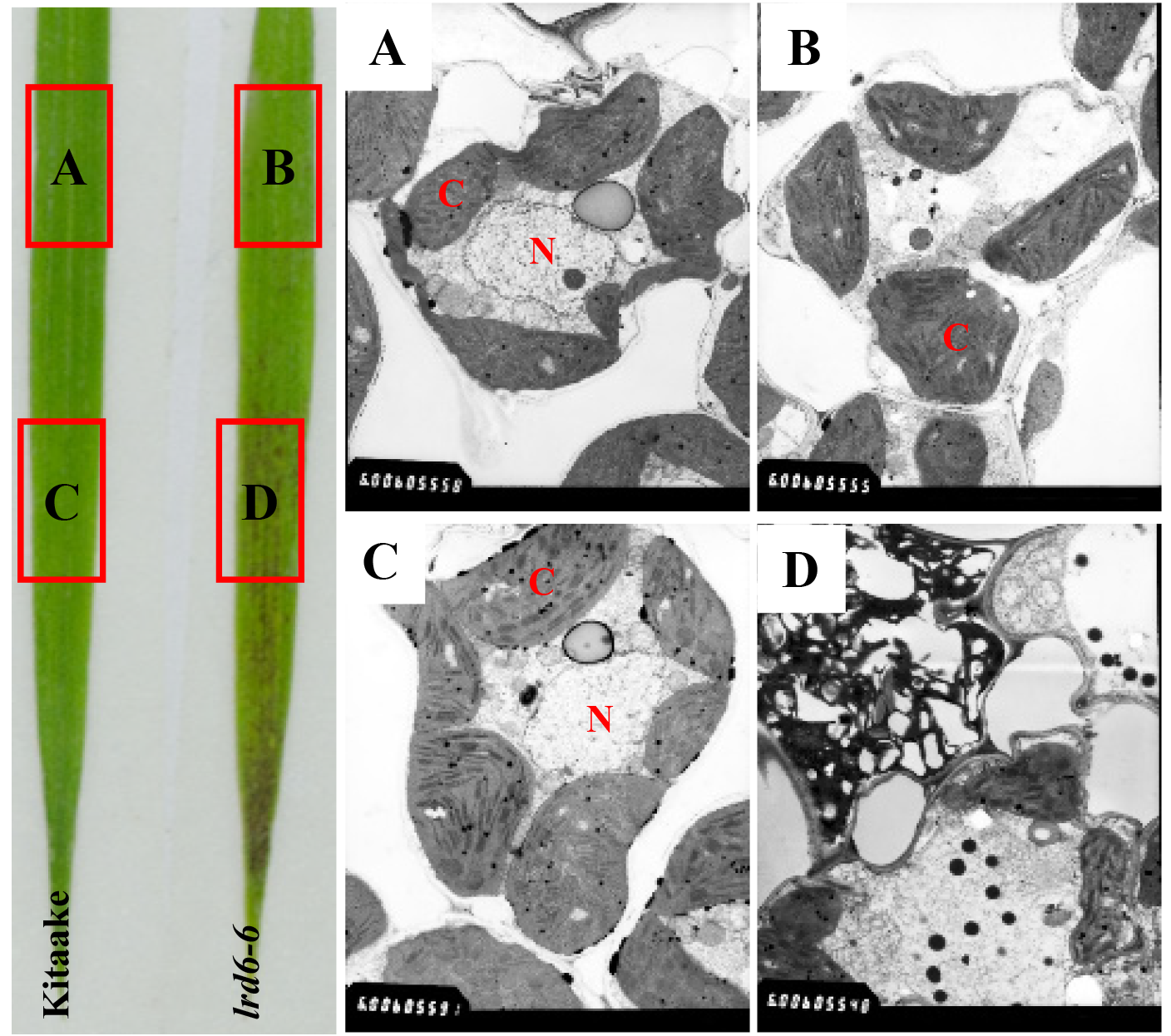

Supplement: S4 Fig — (A) and (B), The cell structures of the leaf part in the absence of lesion spots of Kitaake and lrd6-6. (C) and (D), The cell structures of the leaf part in the presence of lesion spots of lrd6-6 and Kitaake. Magnified 6000 folds. The chloroplast (C) and nucleus (N) are marked in red. (TIF) [file pgen.1006311.s004.tif]

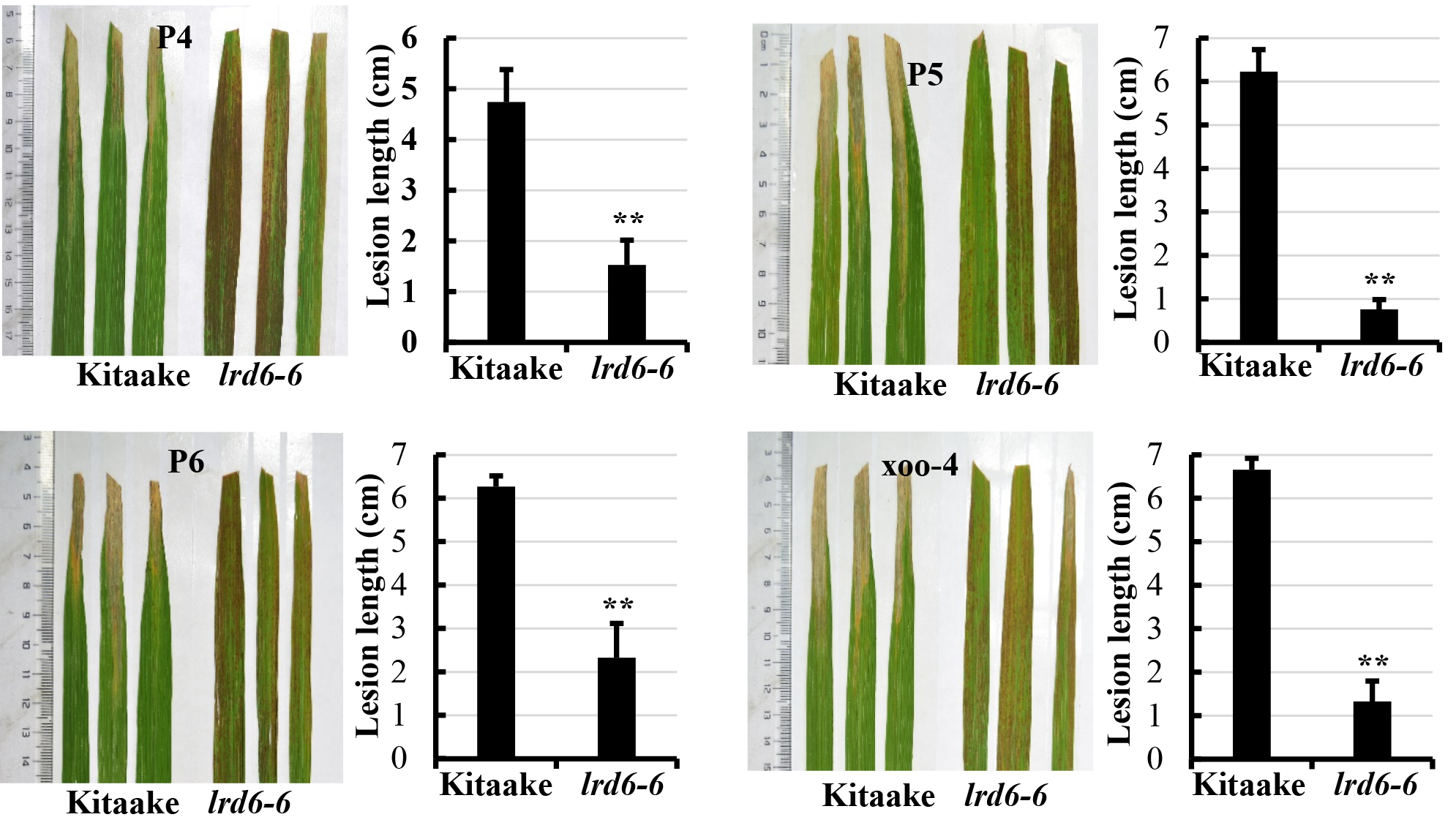

Supplement: S5 Fig — Photographs of representative leaves and disease lesion lengths were taken at 15 d post-inoculation with four Xoo strains compatible with Kitaake (P4, P5, P6 and xoo-4) as indicated. Statistical analysis of the disease lesion lengths was performed on the leaves of inoculated Kitaake and lrd6-6 (error bar, SEM, n > 8). Asterisks denote a significant difference between the lrd6-6 mutant and the wild type as determined by Student’s t-test (**, P < 0.01). (TIF) [file pgen.1006311.s005.tif]

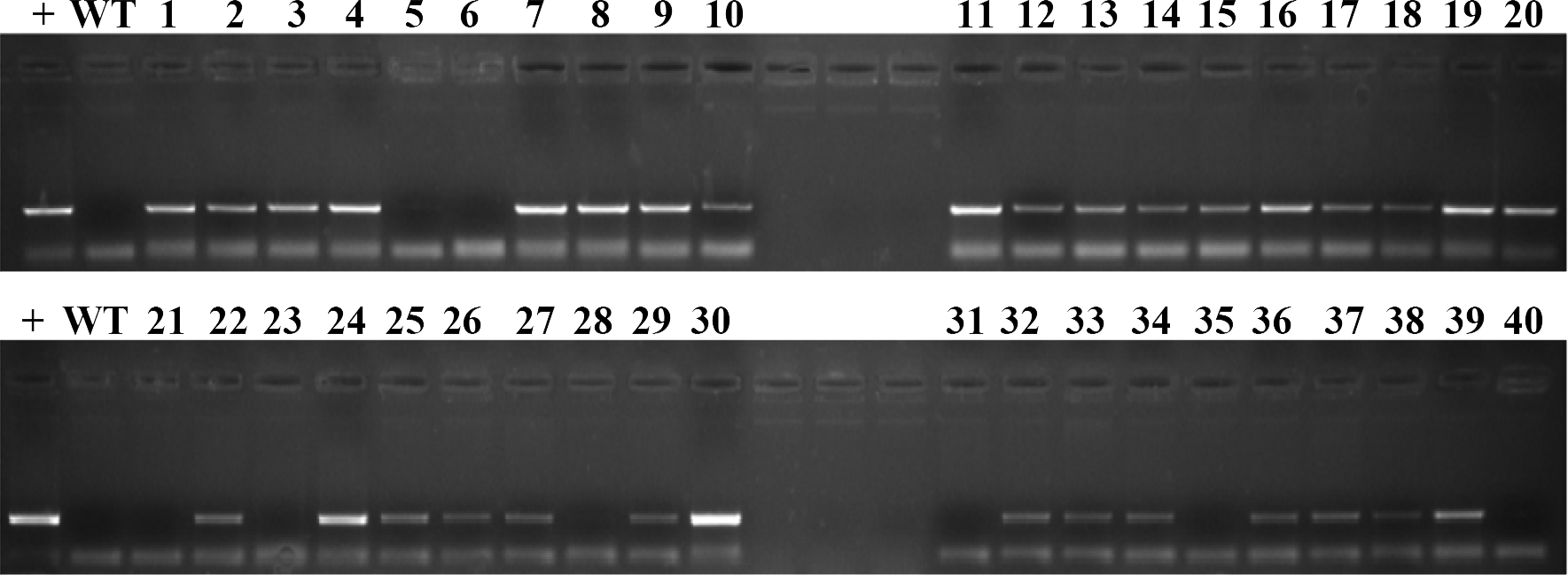

Supplement: S6 Fig — Forty individual F2 plants with cell death phenotypes derived from self-crossed plants heterozygous (Lrd6-6 lrd6-6) for lrd6-6 locus were used for genotyping with the primer pair specific for Hyg. +: Plant containing Hyg used as positive control, WT: Kitaake lacking Hyg used as negative control. (TIF) [file pgen.1006311.s006.tif]

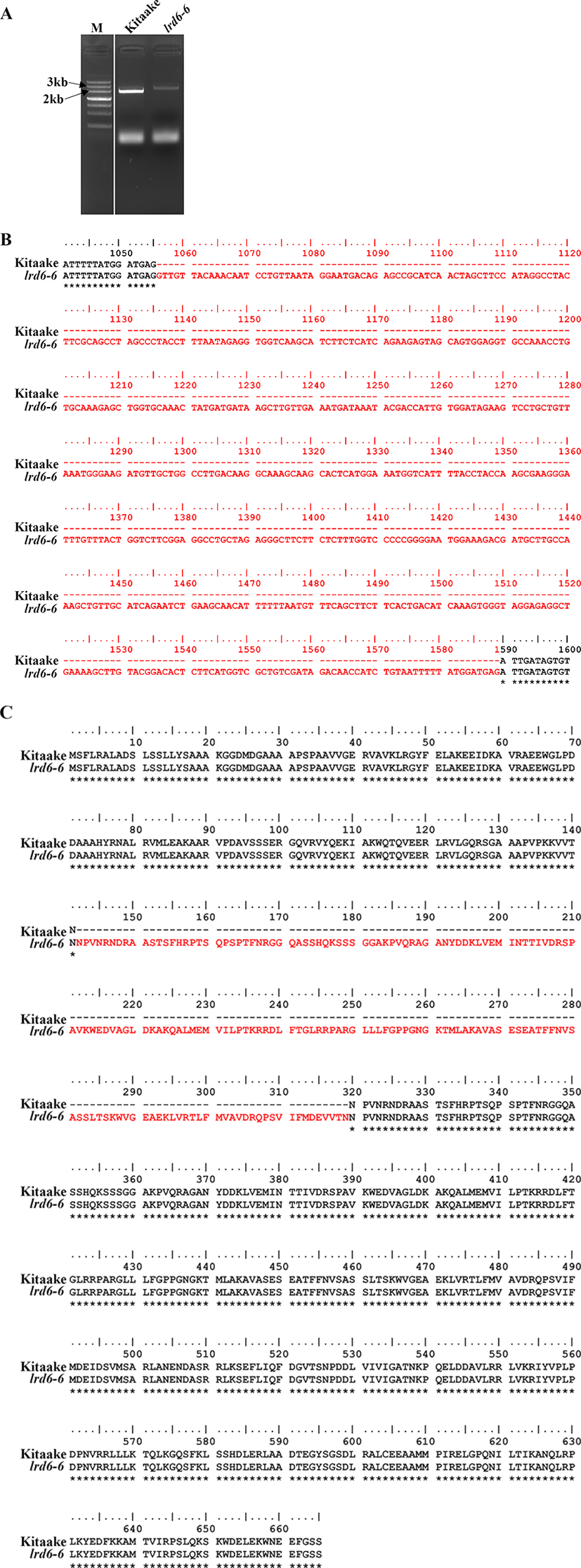

Supplement: S7 Fig — (A) Full-length cDNAs of Os06g03940 were amplified from Kitaake (WT) and lrd6-6 and were separated by agarose gel. (B) Alignment on the cDNA sequences of Os06g03940 between Kitaake and lrd6-6. The insertion with a repeat of 534 bp in the cDNA of Os06g03940 in lrd6-6 is indicated in red. (C) Alignment on the amino acid sequences encoded by Os06g03940 between Kitaake and lrd6-6. The insertion with 178 AAs resulted from the 534 bp repeat of the Os06g03940 cDNA in lrd6-6 is indicated in red. (TIF) [file pgen.1006311.s007.tif]

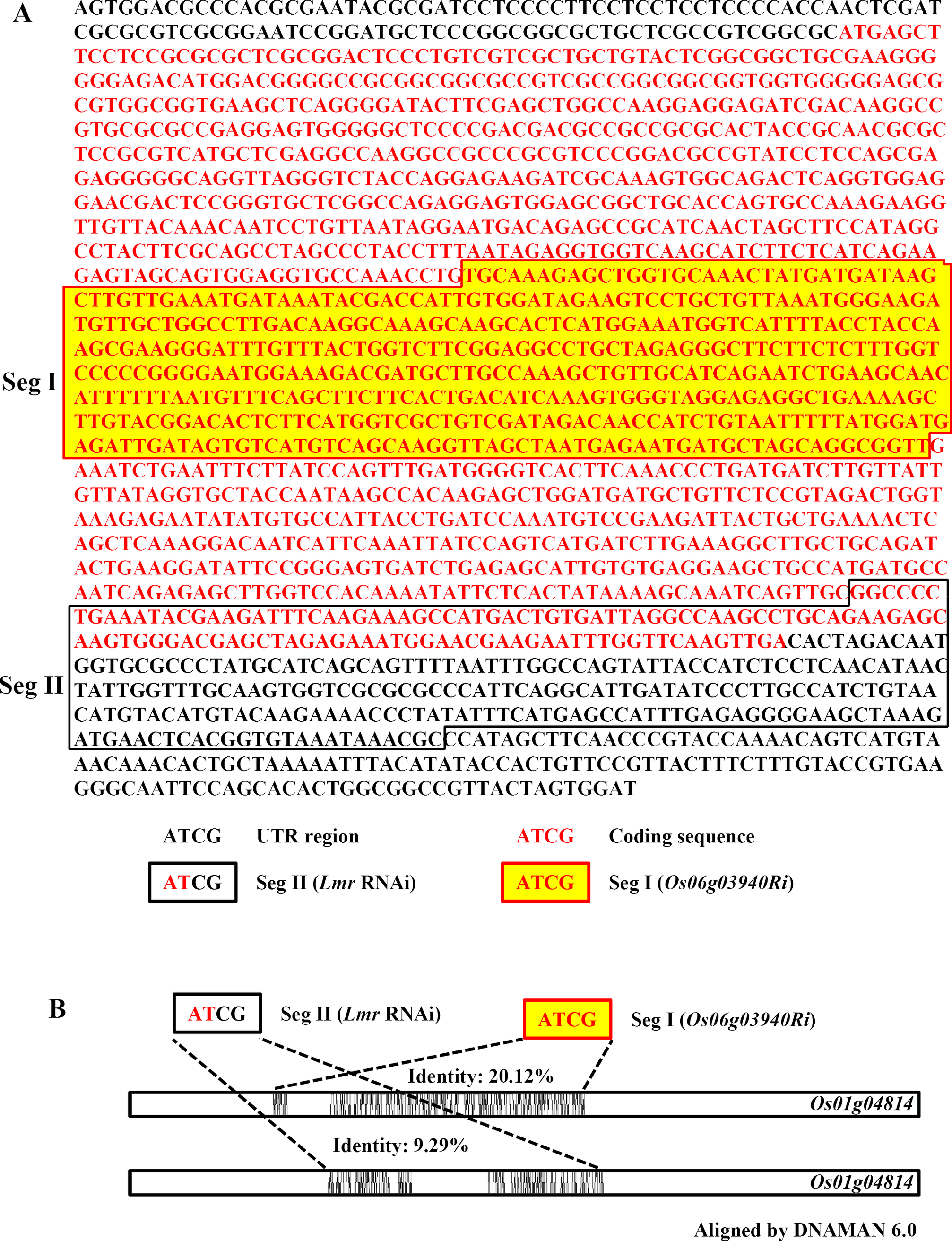

Supplement: S8 Fig — (A) The full-length cDNA of the gene Os06g03940. The coding sequence was marked in red and the UTR regions were in black. The sequence used for generating RNAi construct in this study, named as segment I (Seg I), was boxed with yellow background while the sequence used in Faklh’s paper, named as Seg II, was boxed. Features were respectively indicated below sequence. (B) The cDNA sequence identity analysis between the genes, Os06g03940 and its closest homolog Os01g04814. BLAST alignment was performed by DNAMAN 6.0, the blasting hits in Os01g04814 and the identities were respectively marked. (TIF) [file pgen.1006311.s008.tif]

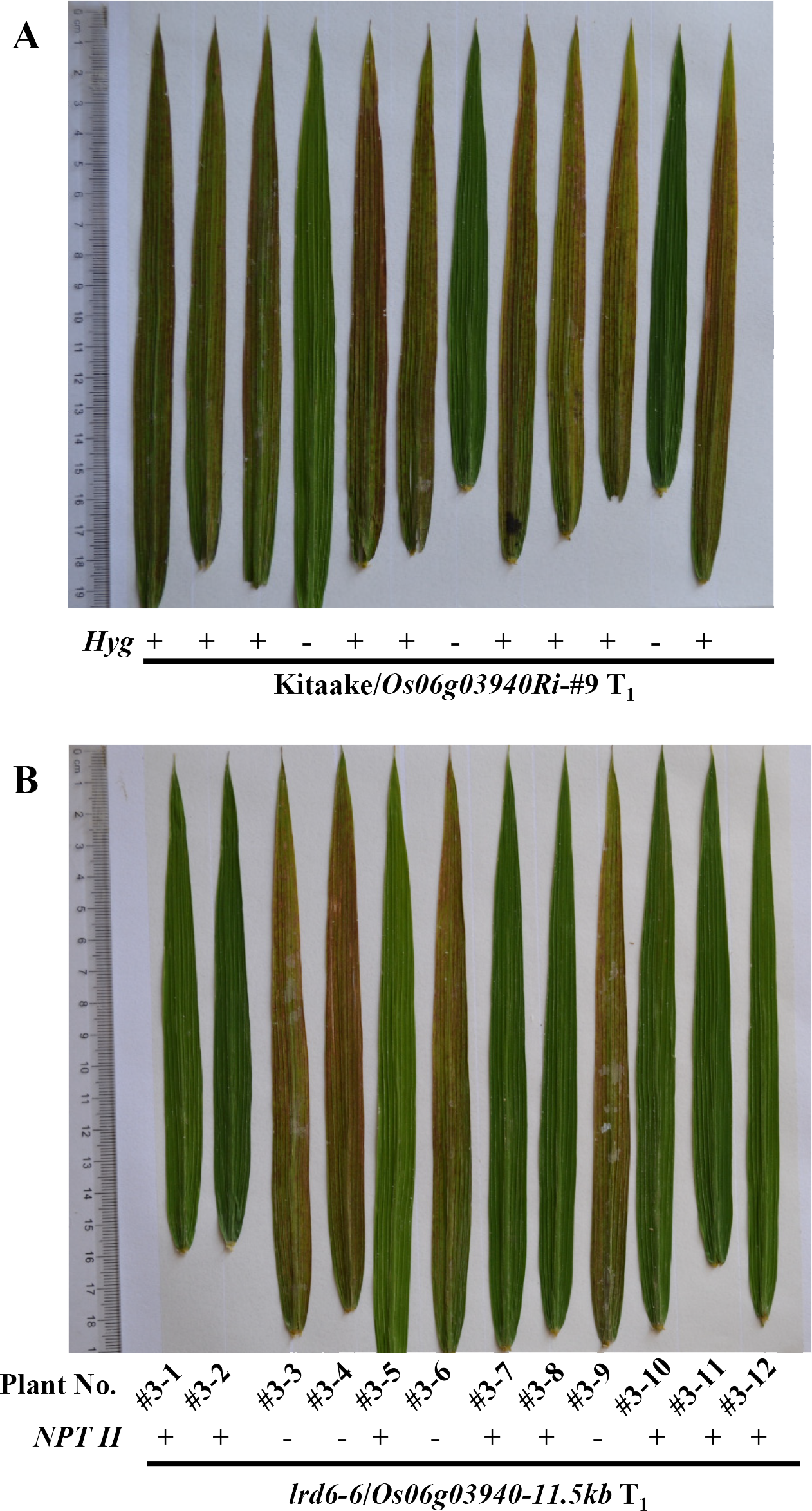

Supplement: S9 Fig — (A) Photograph of representative leaves from 12 individual T1 plants derived from transgenic line #9. PCR-based genotyping with the primer pair specific for the Hyg gene was performed to determine whether the plants contained (represented by ‘+’) or lacked (represented by ‘-’) the transgene Os06g03940Ri. (B) Photograph of representative leaves from 12 T1 plants derived from one transgenic plant (line #3) carrying the transgenic fragment Os06g03940-11.5kb in lrd6-6 genetic background. PCR-based genotyping with the primer pair specific for the Neomycin phosphotransferase II (NPT II) gene was performed to determine whether the plants contained (represented by ‘+’) or lacked (represented by ‘-’) the transgene 06g03940-11.5kb. (TIF) [file pgen.1006311.s009.tif]

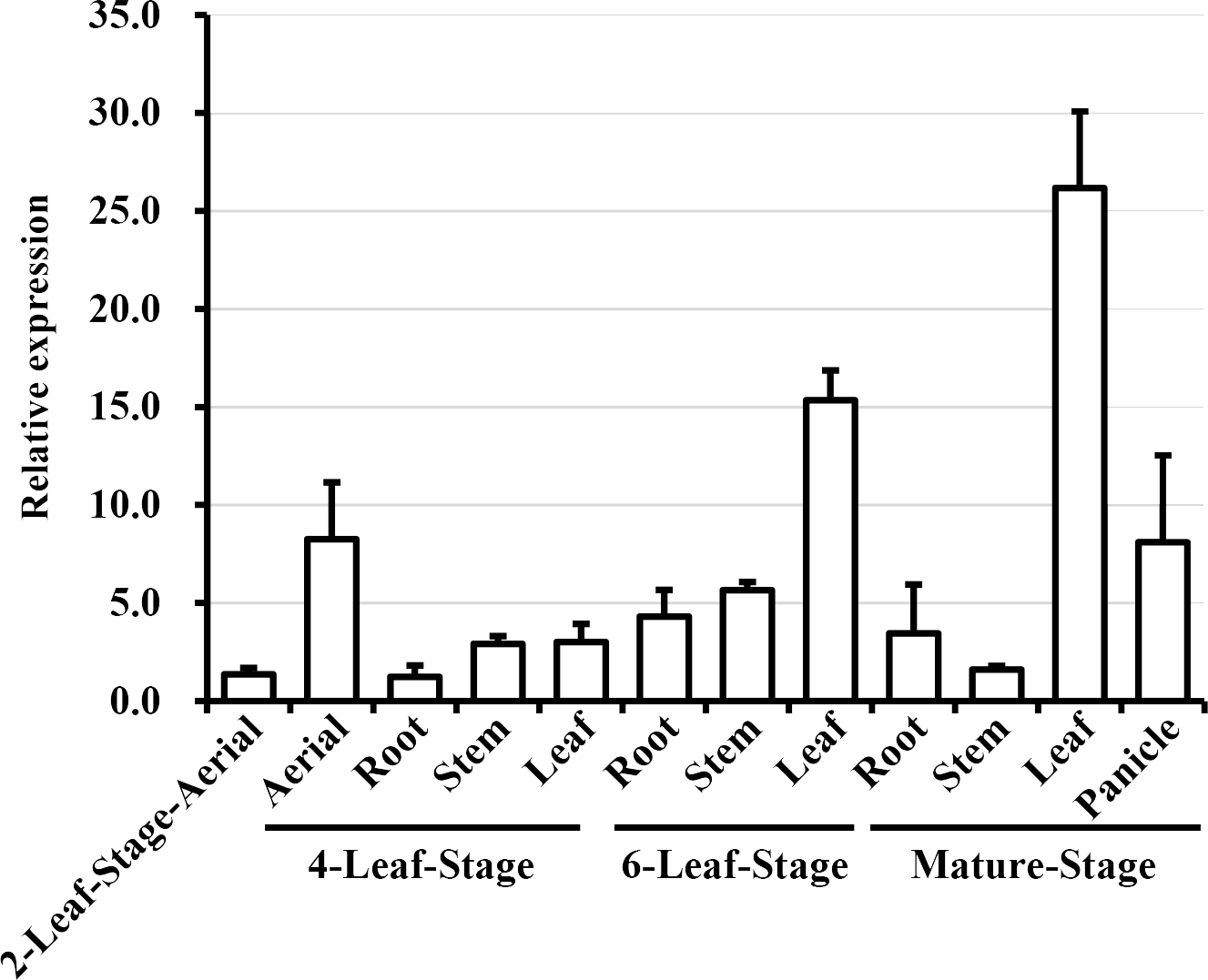

Supplement: S10 Fig — Total RNA was extracted from root, stem, leaf and panicle from Kitaake rice at the two-, four- and six-leaf and mature stages. The qRT-PCR was performed to determine Lrd6-6 expression. The expression level of Lrd6-6 was normalized to the Ubq5 reference gene. Error bars represent the SDs of three biology repeats. (TIF) [file pgen.1006311.s010.tif]

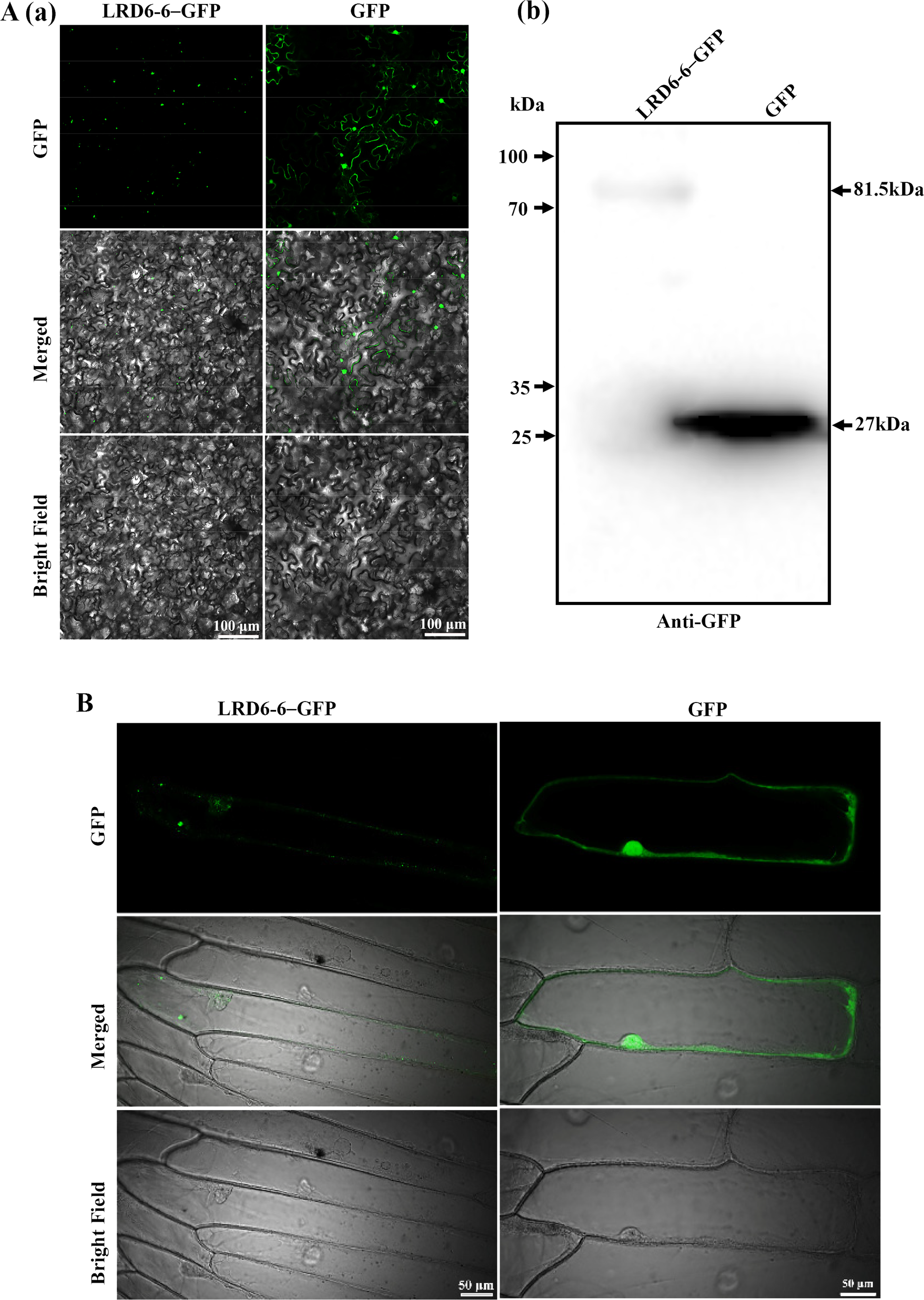

Supplement: S11 Fig — (A) Punctate pattern of LRD6-6–GFP fusion protein in N. benthamiana (a) and the detection of the expressed fusion proteins by anti-GFP (b). The constructs, p35S:LRD6-6-GFP expressing the fusion protein LRD6-6–GFP and p35S:GFP expressing GFP alone, were respectively transformed into N. benthamiana cells. Fluorescence was determined 36 h post transformation. (B) Punctate pattern of LRD6-6–GFP fusion protein in onion epidermal cells. The constructs, p35S:LRD6-6–GFP and p35S:GFP, were respectively transformed into onion epidermal cells. Fluorescence was determined 16 h post transformation. (TIF) [file pgen.1006311.s011.tif]

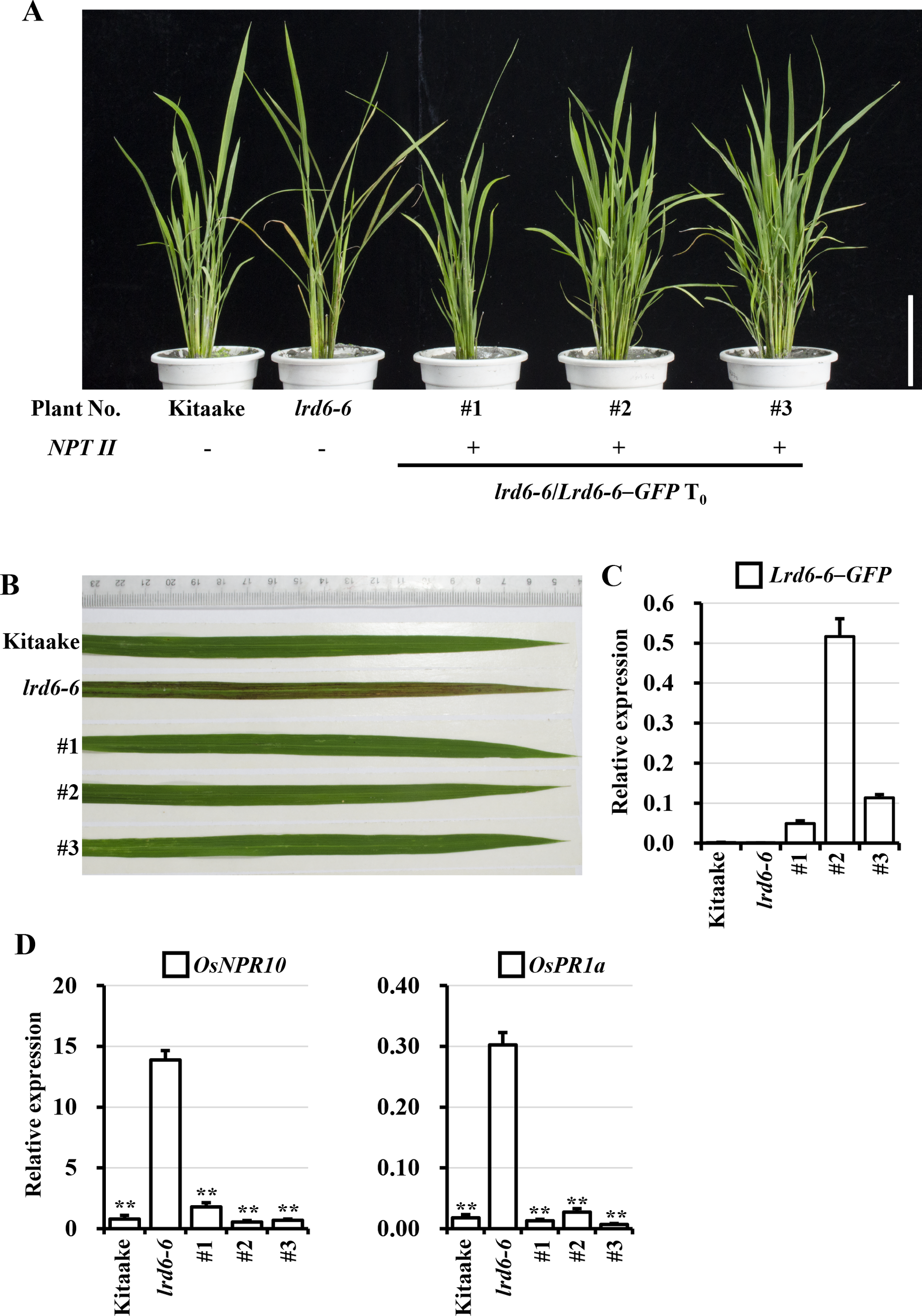

Supplement: S12 Fig — (A) The phenotype of the lrd6-6 mutant plants expressing the Lrd6-6–GFP transgene. Five independent transgenic lrd6-6 lines were found to restore to the wild type phenotype. Photograph of three T0 lines were shown. The wild type Kitaake and the lrd6-6 mutant were also included in the photograph. PCR-based genotyping of the NPT II gene was used to indicate whether the plant contained (‘+’) or lacked (‘-’) the transgenic Lrd6-6-GFP. Bars = 10 cm. (B) Representative leaves from the plants indicated in A. (C) Transcriptional expression level of Lrd6-6–GFP in the transgenic plants determined by qRT-PCR using the GFP specific primers. (D) The expression levels of the PR genes, OsNPR10 and OsPR1a, in the plants shown in (A). The relative expression of the genes was normalized to the Ubq5 reference gene. The error bars represent the SDs of three biological repeats and the expression differences between lrd6-6 was determined by Student’s t-test (**, P < = 0.01). (TIF) [file pgen.1006311.s012.tif]

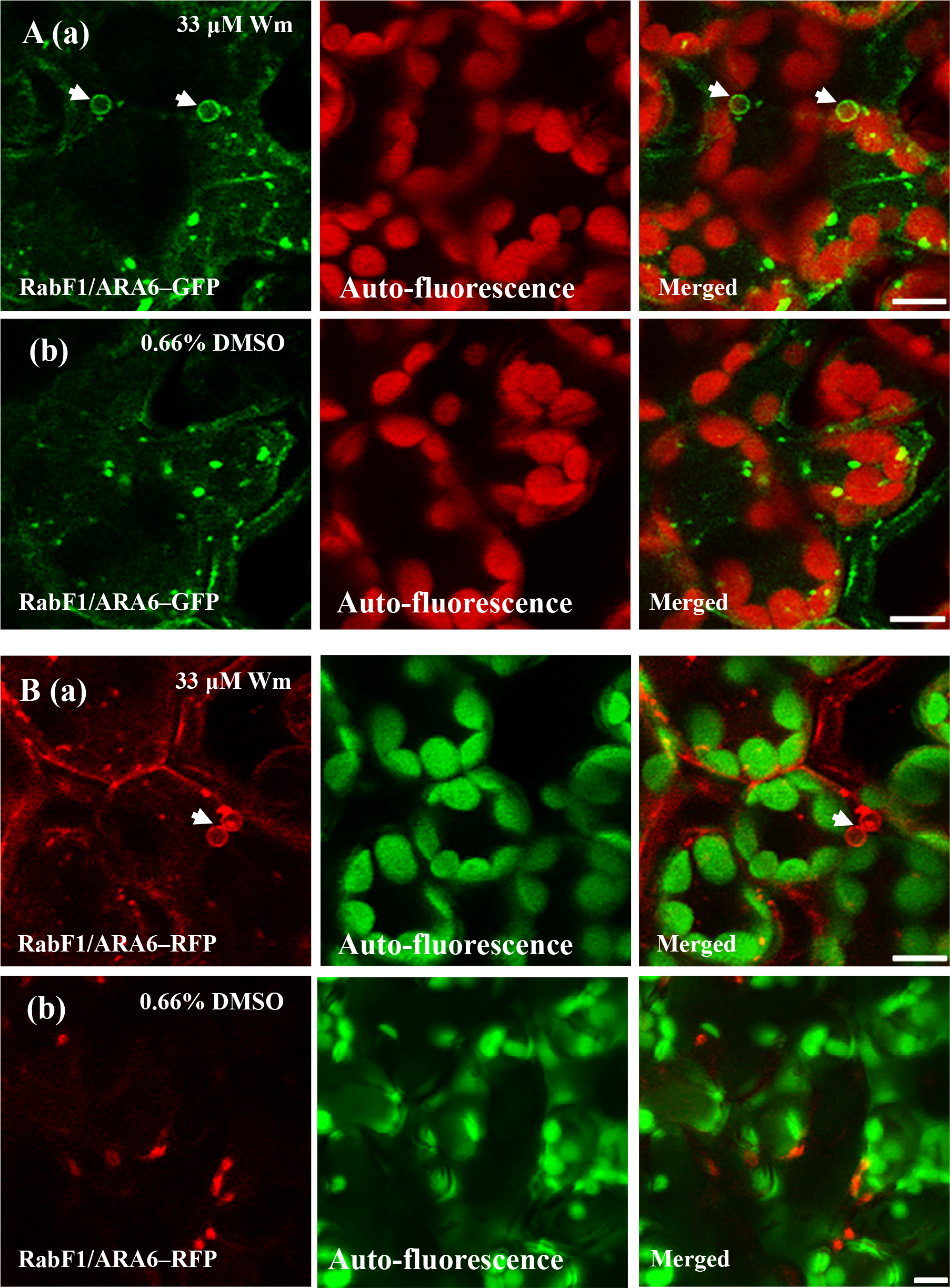

Supplement: S13 Fig — The punctate fluorescence distribution of MVBs-localized marker proteins, RabF1/ARA6-GFP (A) and RabF1/ARA6-RFP (B) turned into ring-like structures as indicated after treated by 33 μM wortmannin (Wm) for 40 min. 0.66% DMSO was used as control for wortmannin treatment. Bars = 10 μm. (TIF) [file pgen.1006311.s013.tif]

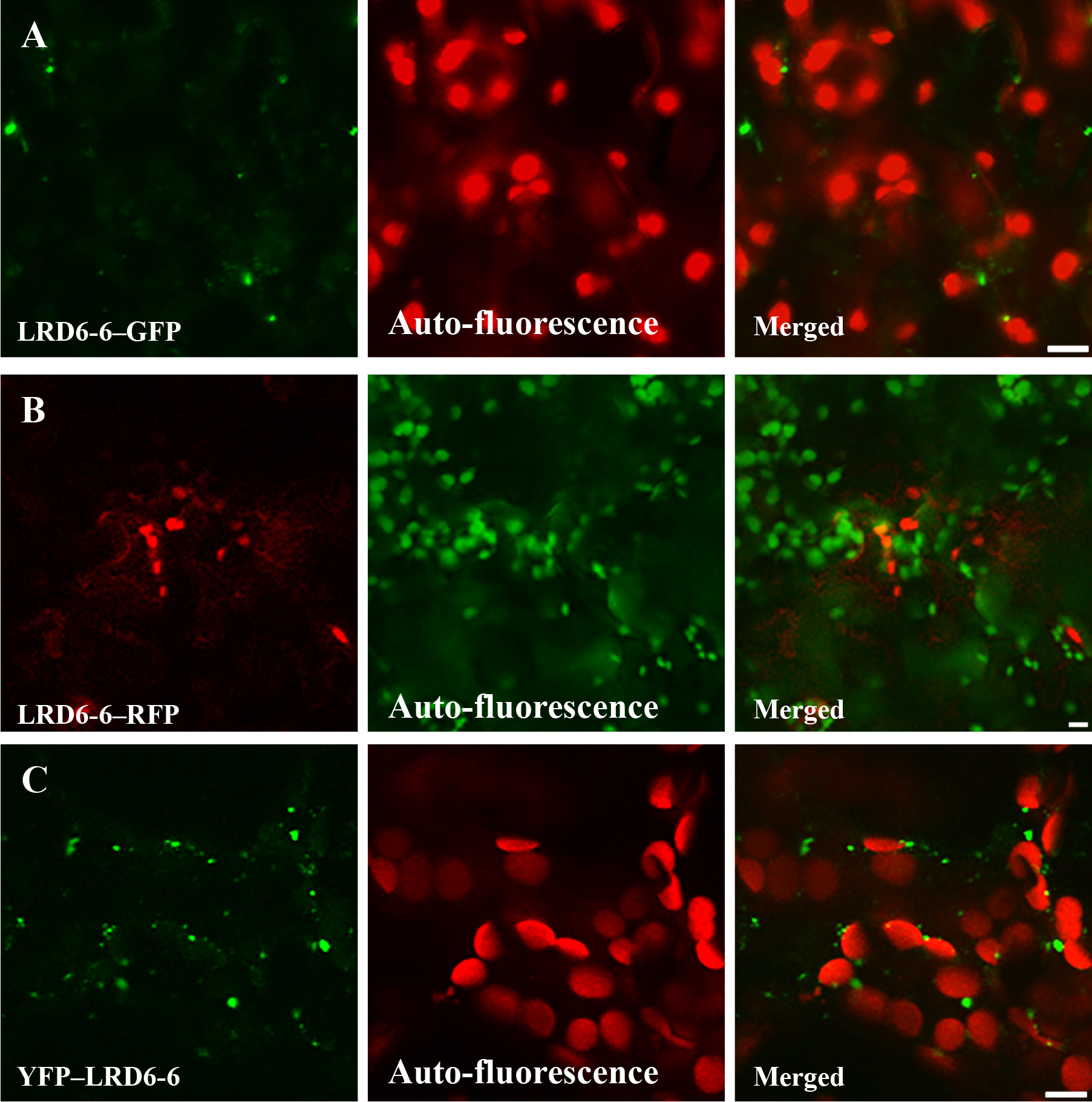

Supplement: S14 Fig — The punctate fluorescence distribution of LRD6-6–GFP, LRD6-6–RFP and YFP–LRD6-6 does not co-locate with the auto-fluorescence of chlorophyll in N. benthamiana. Bars = 10 μm. (TIF) [file pgen.1006311.s014.tif]

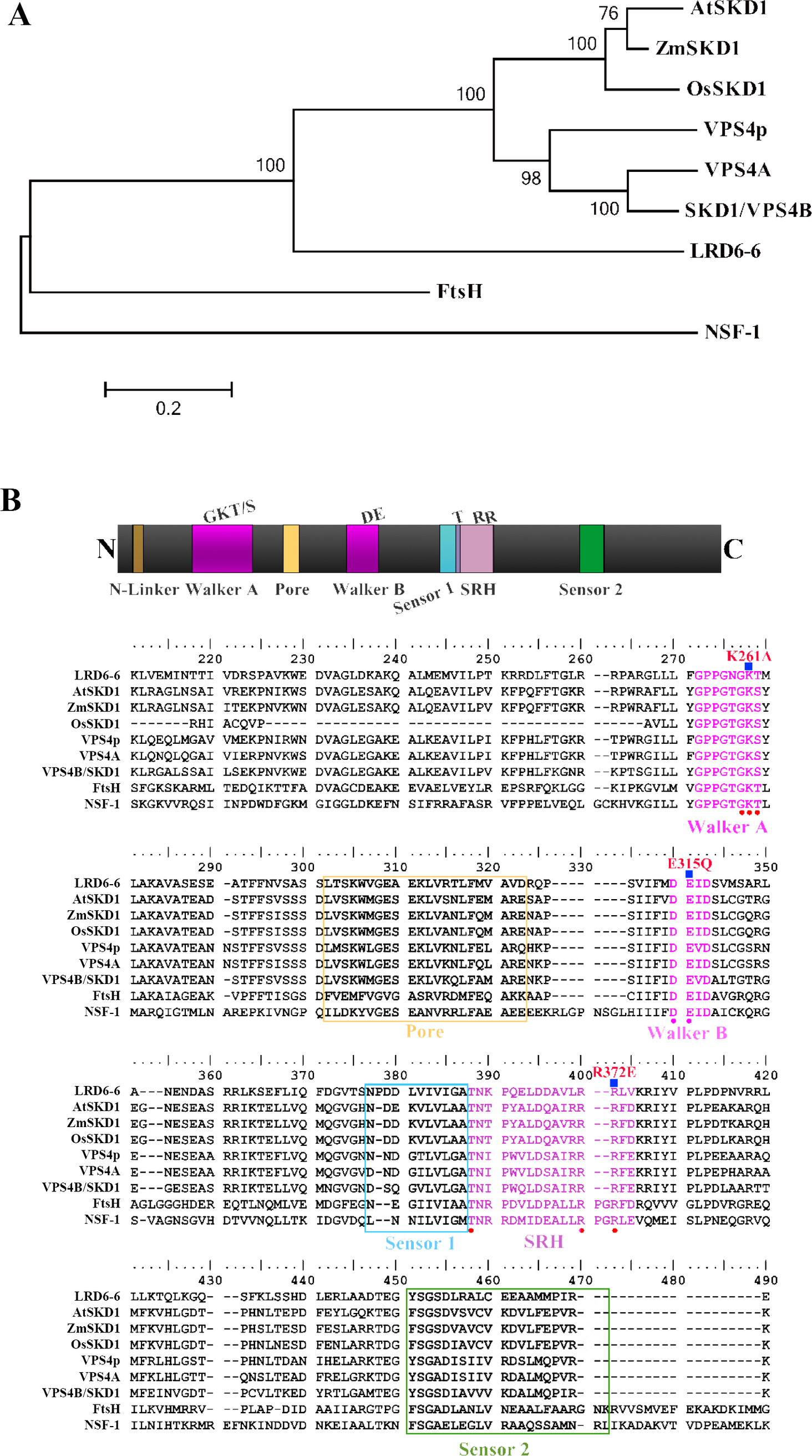

Supplement: S15 Fig — (A) Phylogenetic analysis of LRD6-6 with the AAA ATPases, AtSKD1, ZmSKD1, VPS4A, VPS4B/SKD1, VPS4p, FtsH, NSF-1 and the AtSKD1 homolog OsSKD1 using Mega5.1. Bootstrap values are indicated beside each branch. (B) Structure analysis of the LRD6-6 protein. The characteristics of typical AAA ATPase (upper panel) and sequence alignment analysis on LRD6-6 with some known AAA ATPases (lower panel) are respectively shown. The key elements of Walker A and B motifs, the Pore, Sensors 1 and 2, and the second region of homology (SRH) are respectively marked in the figure. The conserved residues, K261A, E315Q and R372E in LRD6-6 are respectively indicated. (TIF) [file pgen.1006311.s015.tif]

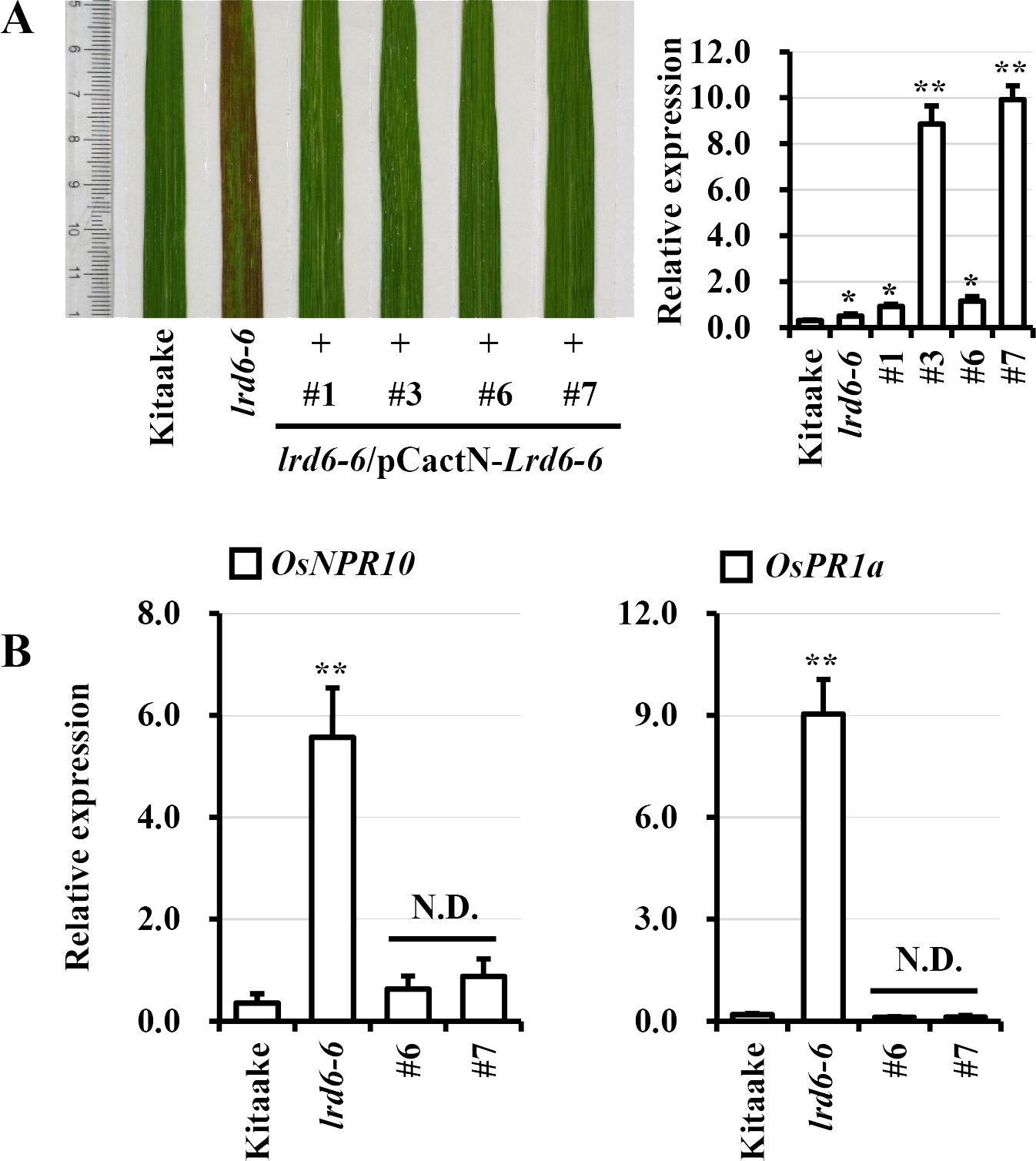

Supplement: S16 Fig — (A) Expression of Lrd6-6 inhibits the spontaneous cell death of the lrd6-6 mutant. PCR-based genotyping with the primer pair specific for the Neomycin phosphotransferase II (NPT II) gene was performed to determine whether the plants contained (represented by ‘+’) or lacked (represented by ‘-’) the transgene. (B) Expression of Lrd6-6 compromises the expression of PR genes in the lrd6-6 mutant. The expression level of PR genes, OsNPR10 and OsPR1a, in the plants was determined by qRT-PCR. The expression was normalized to the Ubp5 reference gene. The error bars represent the SDs of three biology repeats and the expression differences was determined by Student’s t-test (*, P < = 0.05; **, P < = 0.01; N.D., No significantly difference). (TIF) [file pgen.1006311.s016.tif]

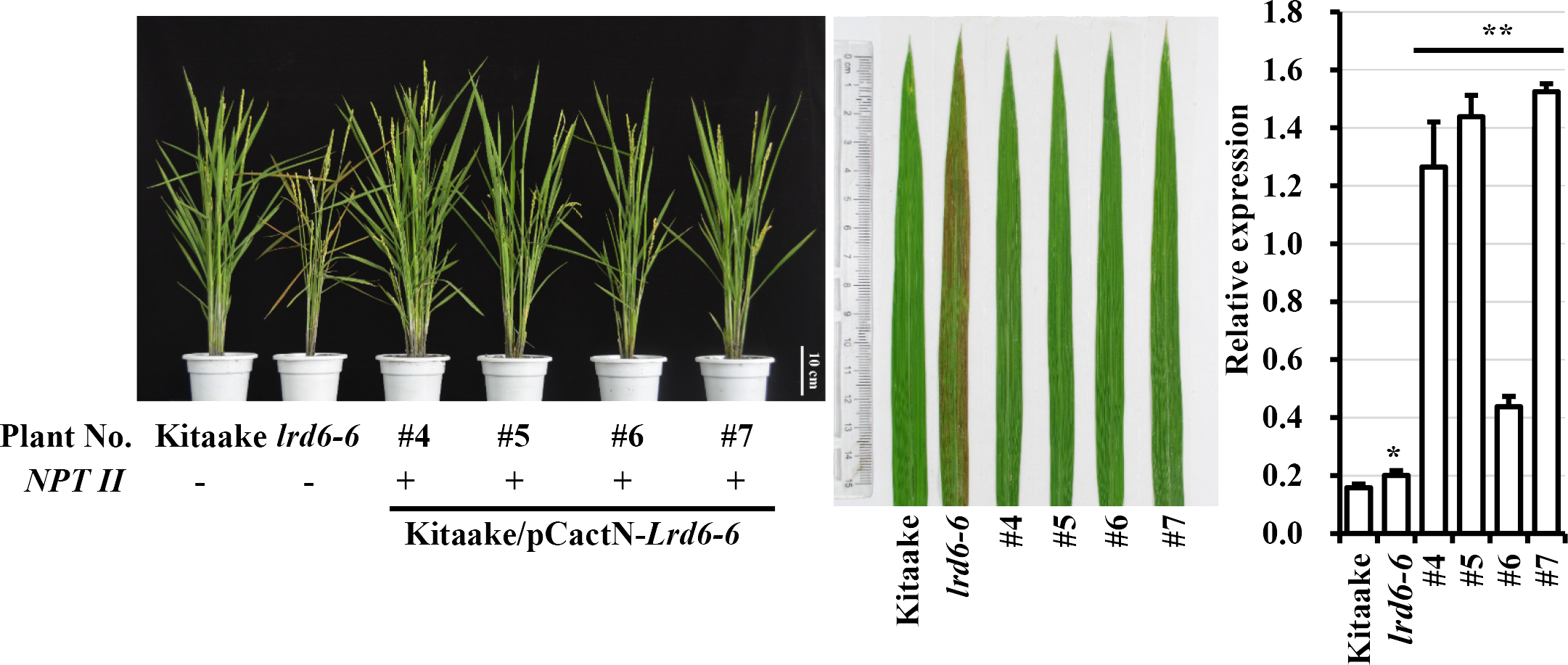

Supplement: S17 Fig — PCR-based genotyping with the primer pair specific for the Neomycin phosphotransferase II (NPT II) gene was performed to determine whether the plants contained (represented by ‘+’) or lacked (represented by ‘-’) the transgene. The expression level of Lrd6-6 in plants was determined by qRT-PCR. The expression was normalized to the Ubp5 reference gene. The error bars represent the SDs of three biology repeats and the expression differences was determined by Student’s t-test (*, P < = 0.05; **, P < = 0.01). (TIF) [file pgen.1006311.s017.tif]

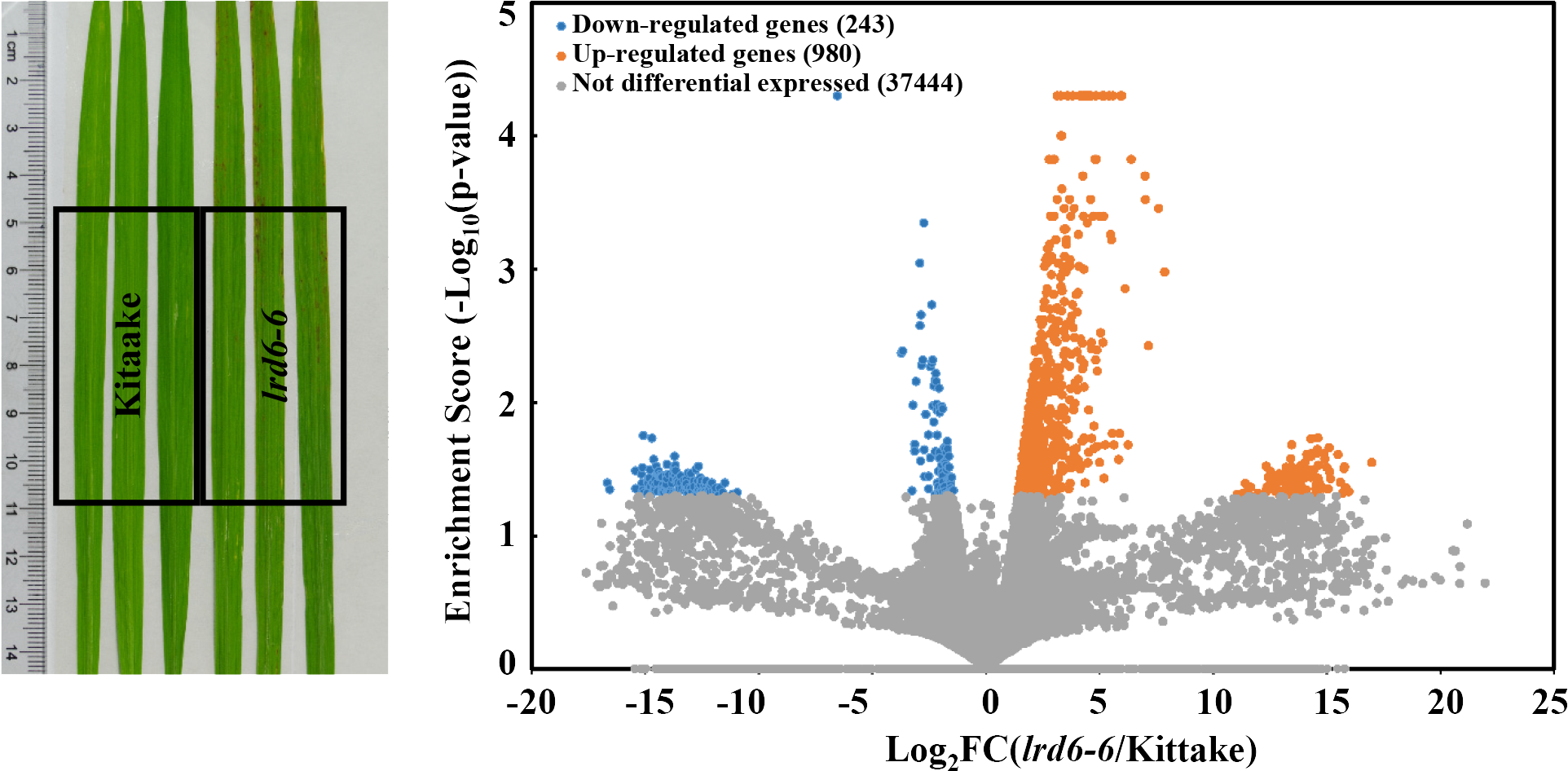

Supplement: S18 Fig — Genome-wide transcript analysis was performed on the lrd6-6 mutant and Kitaake. The leaf part (marked in the squares) from Kitaake and the lrd6-6 mutant were sampled when spontaneous cell death started to appear on the leaf of lrd6-6. A total of 1223 DEGs were obtained. Of them, 980 DEGs were up-regulated and 243 were down-regulated in lrd6-6 [P < = 0.05, Log2FC (lrd6-6/Kitaake) > 1]. (TIF) [file pgen.1006311.s018.tif]

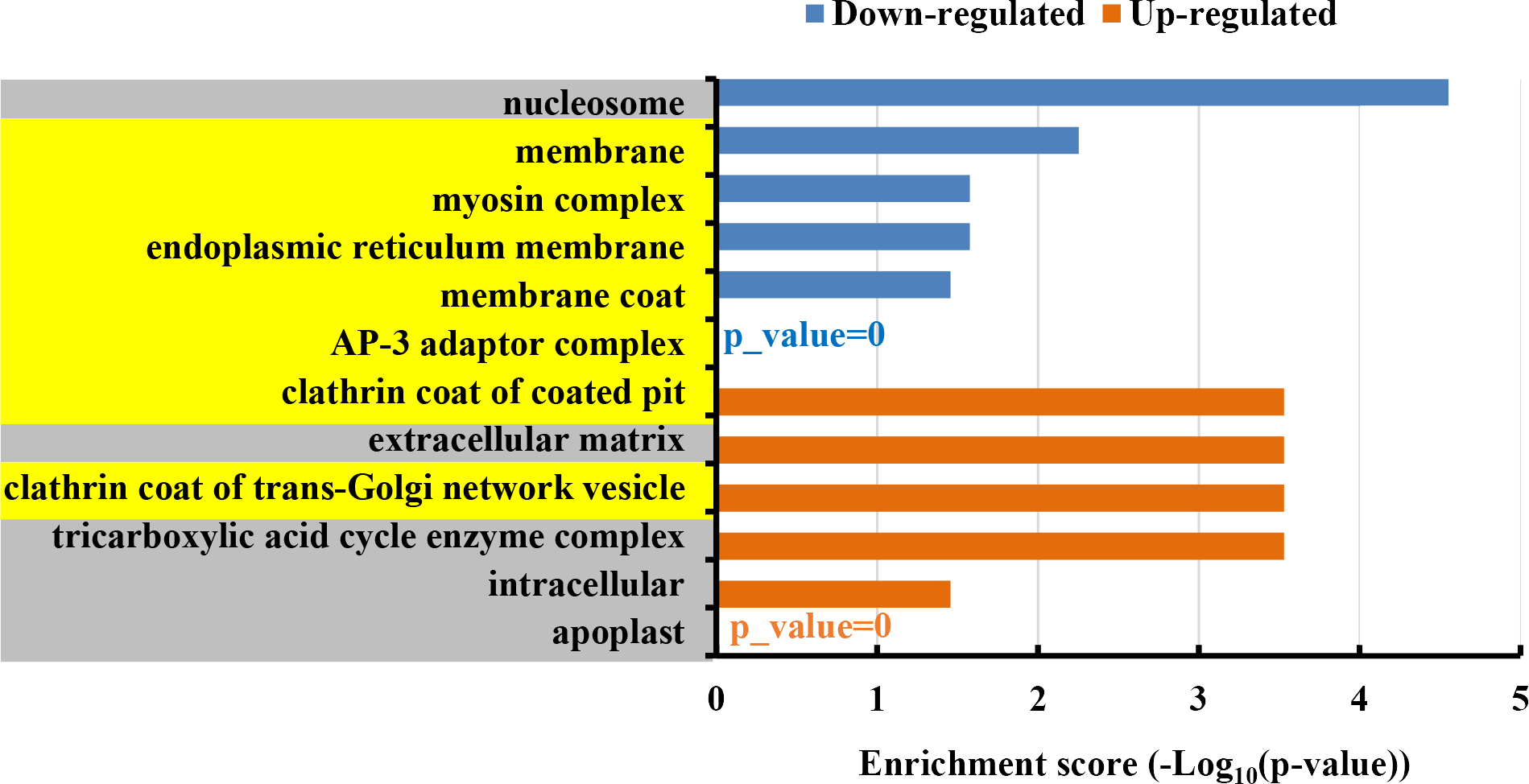

Supplement: S19 Fig — The GO terms associated with MVBs-mediated vesicular trafficking are highlighted in yellow background. (TIF) [file pgen.1006311.s019.tif]

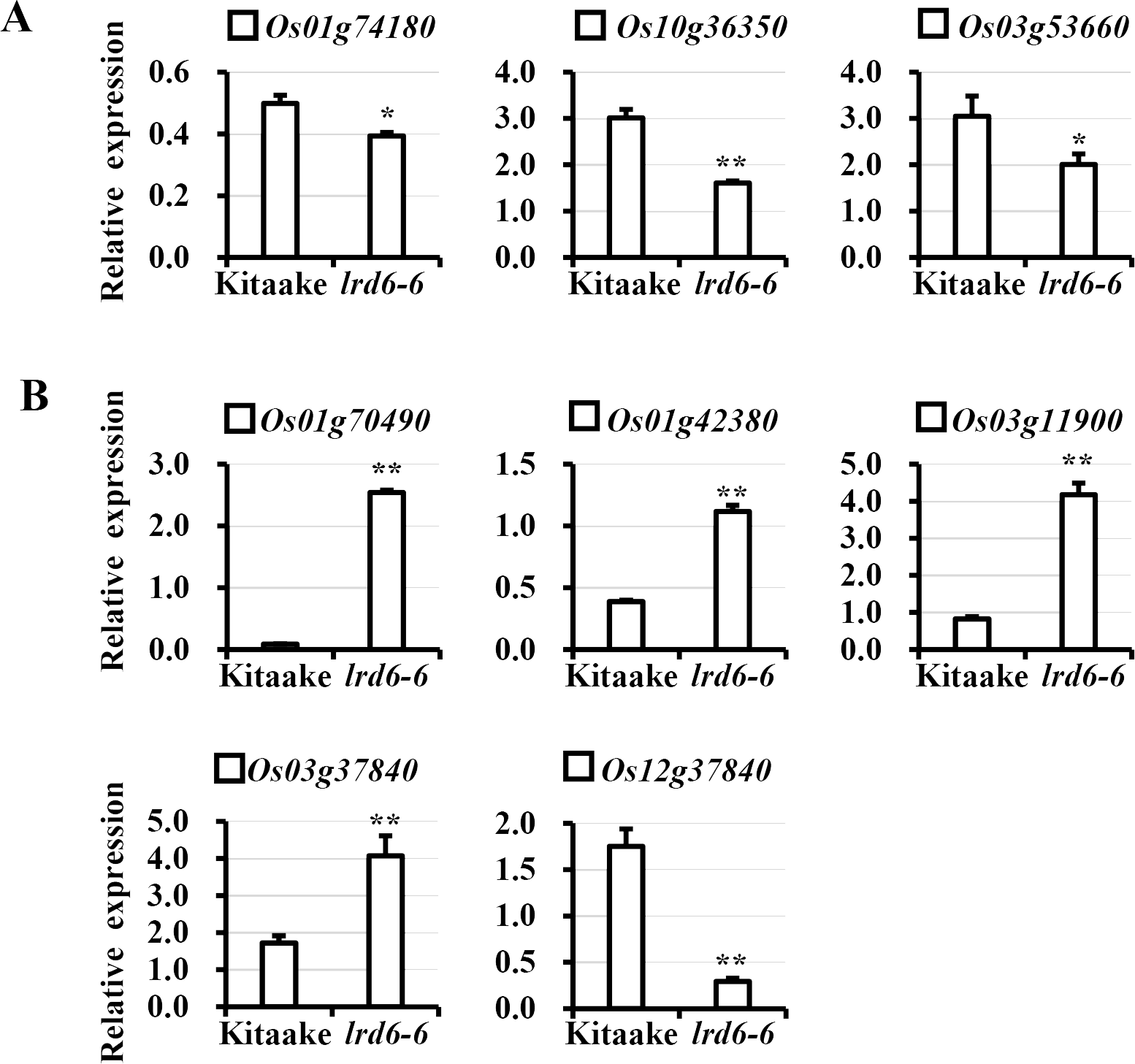

Supplement: S20 Fig — (A) Analyses on three DEGs coding for MVBs-mediated vesicular trafficking pathway components. (B) Analyses on five DEGs coding the transmembrane transporters which were predicted to be transported by MVBs-mediated vesicular trafficking. Expression analyses were performed by using qRT-PCR. RNA samples were prepared from leaf samples of the lrd6-6 mutant and Kitaake collected as used for RNA-seq analysis. The expression was normalized to the Ubp5 reference gene. The error bars represent the SDs of three biology repeats and the expression differences was determined by Student’s t-test (*, P < = 0.05; **, P < = 0.01). (TIF) [file pgen.1006311.s020.tif]

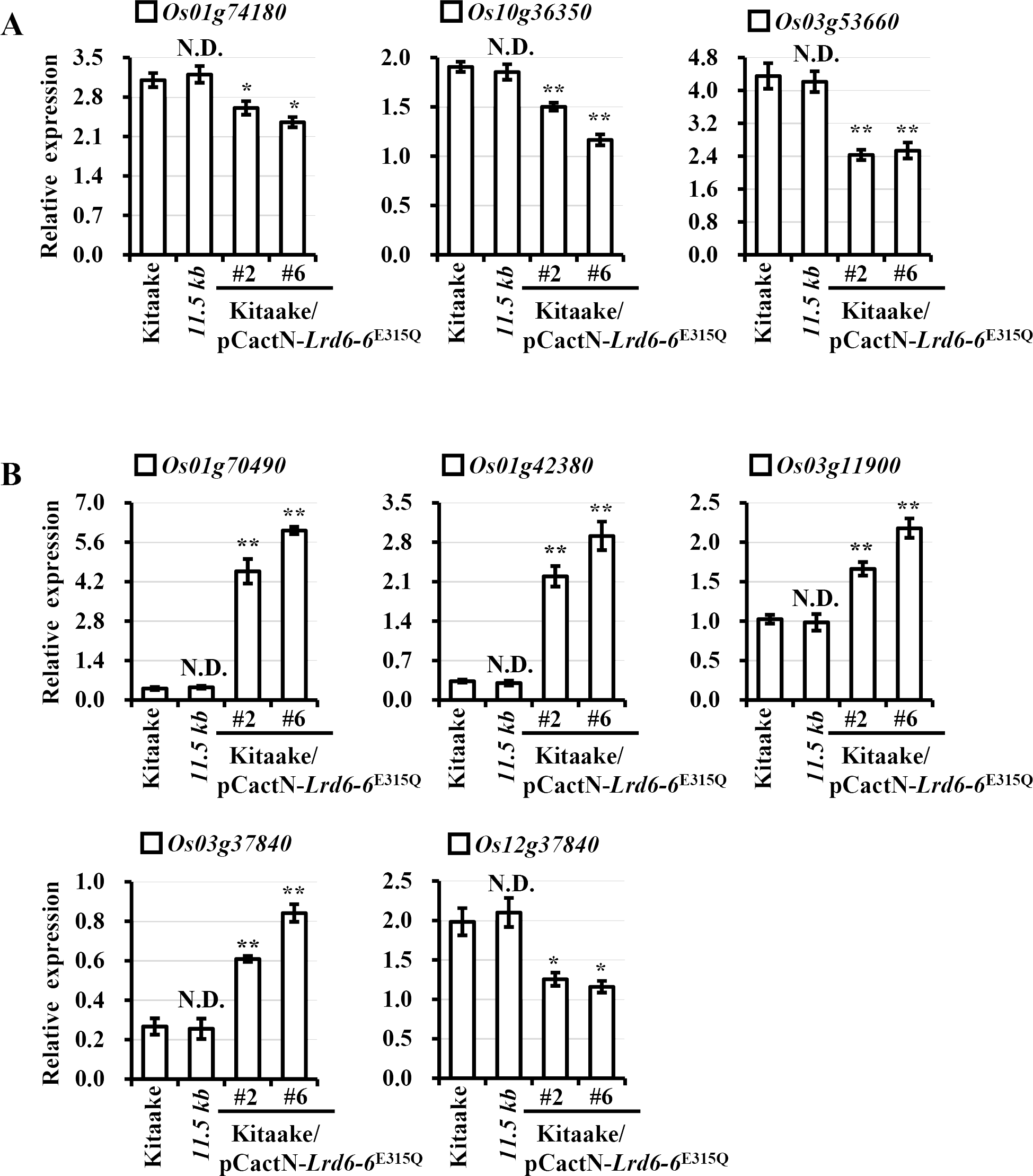

Supplement: S21 Fig — (A) Analyses on three DEGs coding for MVBs-mediated vesicular trafficking pathway components. (B) Analyses on five DEGs coding the transmembrane transporters which were predicted to be transported by MVBs-mediated vesicular trafficking. Expression analyses were performed by using qRT-PCR. The expression was normalized to the Ubp5 reference gene. The error bars represent the SDs of three biology repeats and the expression differences was determined by Student’s t-test (*, P < = 0.05; **, P < = 0.01). (TIF) [file pgen.1006311.s021.tif]

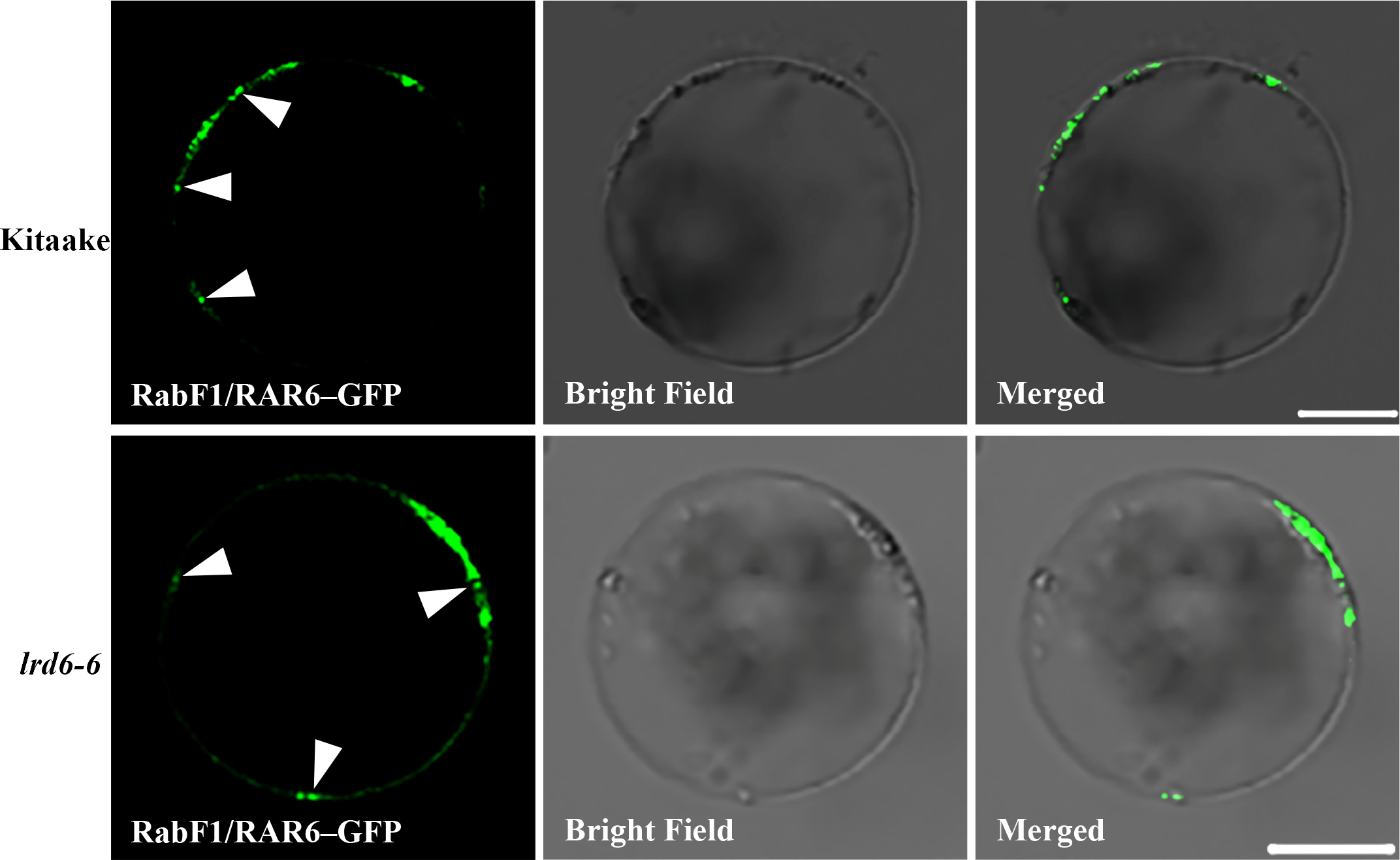

Supplement: S22 Fig — The MVBs marker protein RabF1/ARA6–GFP was transiently expressed in the protoplast cells prepared from the wild type Kitaake and the lrd6-6 mutant, respectively, through PEG-mediated transformation. Fluorescence was determined 16 h post transformation. Arrowheads in the left panels point to some of the punctate MVBs marked by the RabF1/ARA6–GFP protein. Bars = 10 μm. (TIF) [file pgen.1006311.s022.tif]

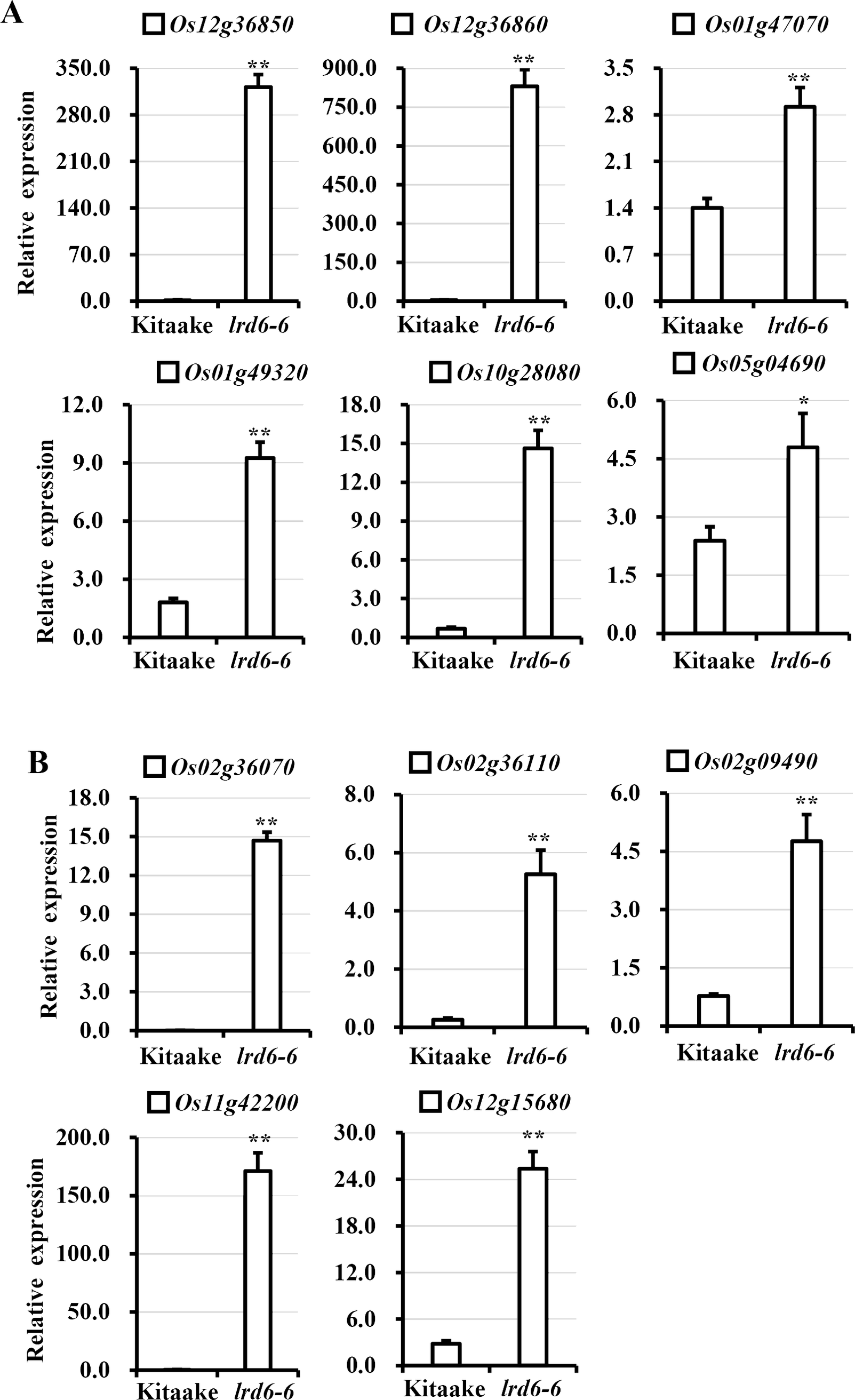

Supplement: S23 Fig — (A) Expression comparison of the DEGs encoding the PR genes, Os12g36850, Os12g36860 and Os01g47070, and the chitinase genes, Os01g49320, Os10g28080 and Os05g04690 between lrd6-6 and Kitaake. (B) Expression comparison of the DEGs associated with ROS metabolism between the lrd6-6 mutant and Kitaake. Expression analyses were performed by using qRT-PCR. RNA samples were prepared from leaf samples of lrd6-6 and Kitaake collected as used for RNA-seq analysis. The expression was normalized to the Ubp5 reference gene. The error bars represent the SDs of three biology repeats and the expression differences was determined by Student’s t-test (*, P < = 0.05; **, P < = 0.01). (TIF) [file pgen.1006311.s023.tif]

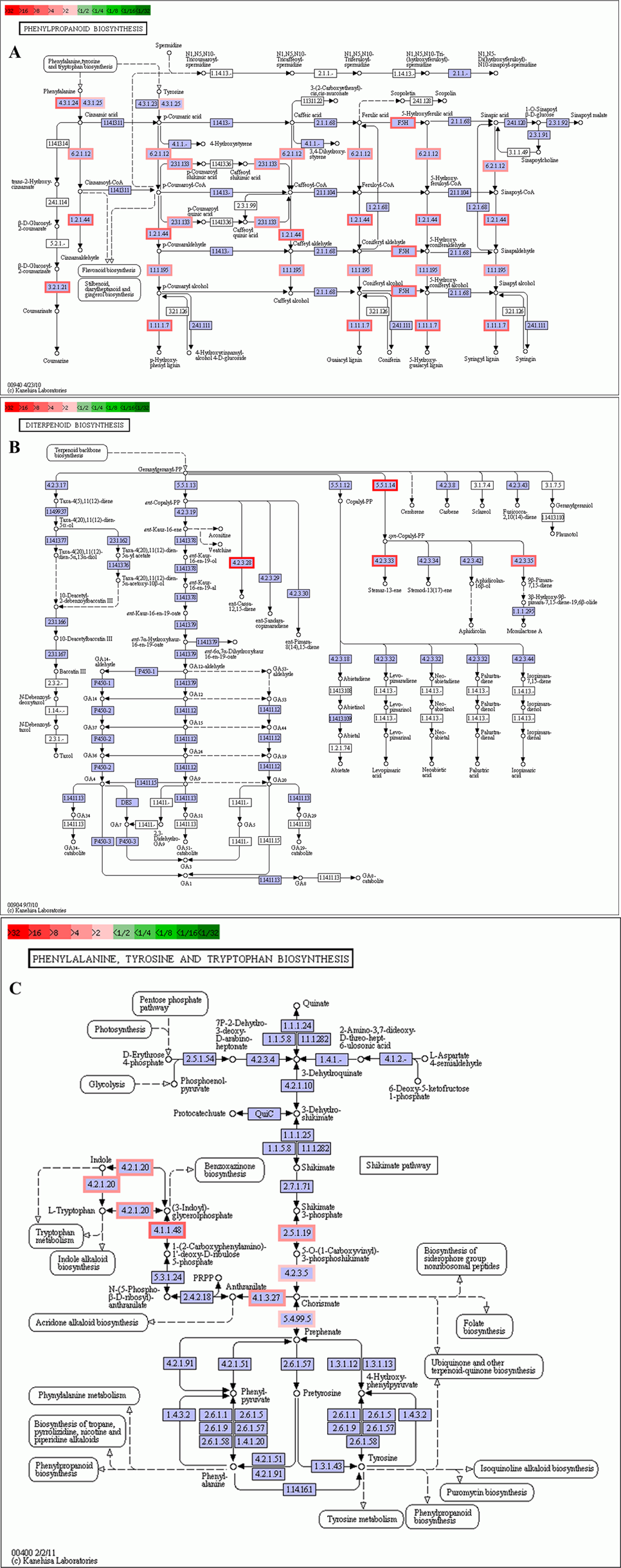

Supplement: S24 Fig — Pathway analysis was performed on the DEGs obtained from RNA-seq data by using the KEGG database. Three main pathways, phenylpropanoid biosynthesis (A), diterpenoid biosynthesis (B) and phenylalanine, tyrosine and tryptophan biosynthesis (C), were clearly up-regulated in the lrd6-6 mutant compared with wild type Kitaake. The enzyme genes boxed with different gradients of colors represent various levels of expression change as indicated. (TIF) [file pgen.1006311.s024.tif]

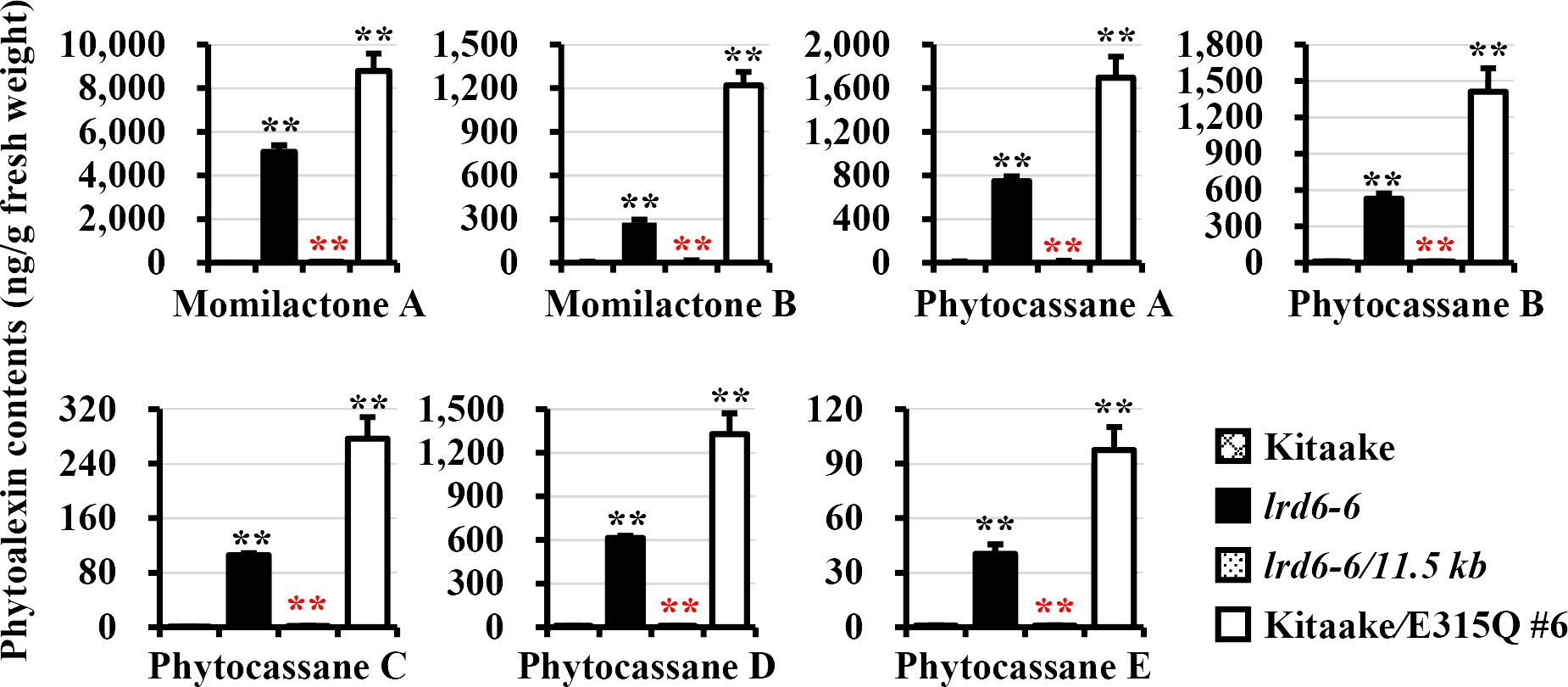

Supplement: S25 Fig — Leaf samples of the wild type Kitaake, the lrd6-6 mutant, the lrd6-6 plants expressing the transgene Os06g03940-11.5kb (lrd6-6/11.5kb) and Kitaake plants expressing Lrd6-6E315Q (Kitaake/E315Q #6) were respectively collected and subjected to phytoalexins determination. The data was obtained from four biological replicates. The error bars indicate the SDs. The black ** indicates significant increase between wild type Kitaake while the red ** represents the significant decrease when compared with the lrd6-6 mutant (Student’s t-test; **, P < 0.01). (TIF) [file pgen.1006311.s025.tif]

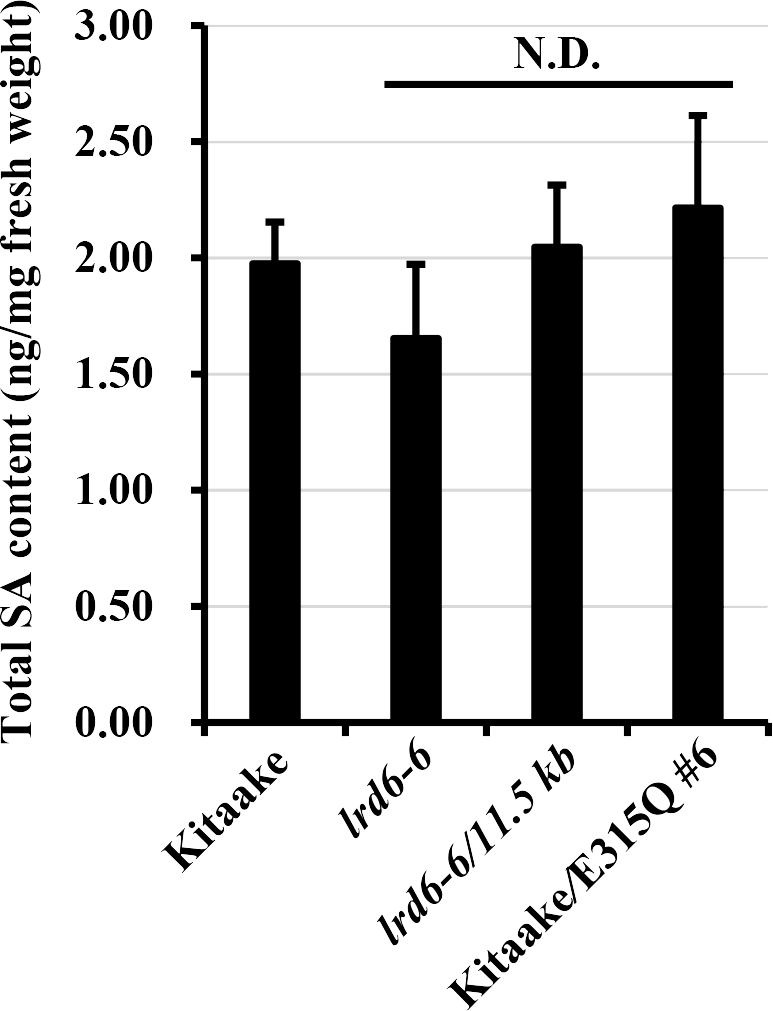

Supplement: S26 Fig — Leaf samples of those materials were collected respectively and subjected to total SA determination. The error bars represent the SDs of three biology repeats and the data was compared by Student’s t-test to detect whether statistical differences exited. N.D. means no significantly difference. (TIF) [file pgen.1006311.s026.tif]

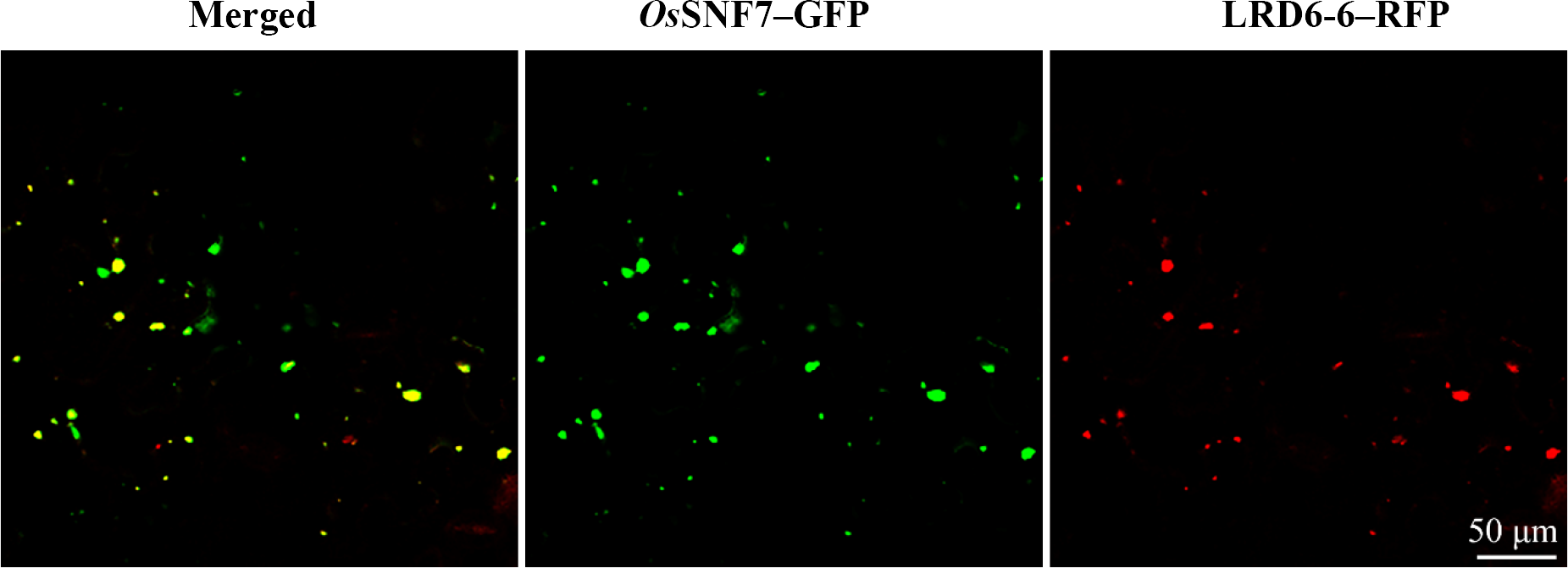

Supplement: S27 Fig — The proteins, OsSNF7–GFP and LRD6-6–RFP, were co-expressed in N. benthamiana through Agrobacterium-mediated transformation. Fluorescence was determined 36 h post transformation. (TIF) [file pgen.1006311.s027.tif]

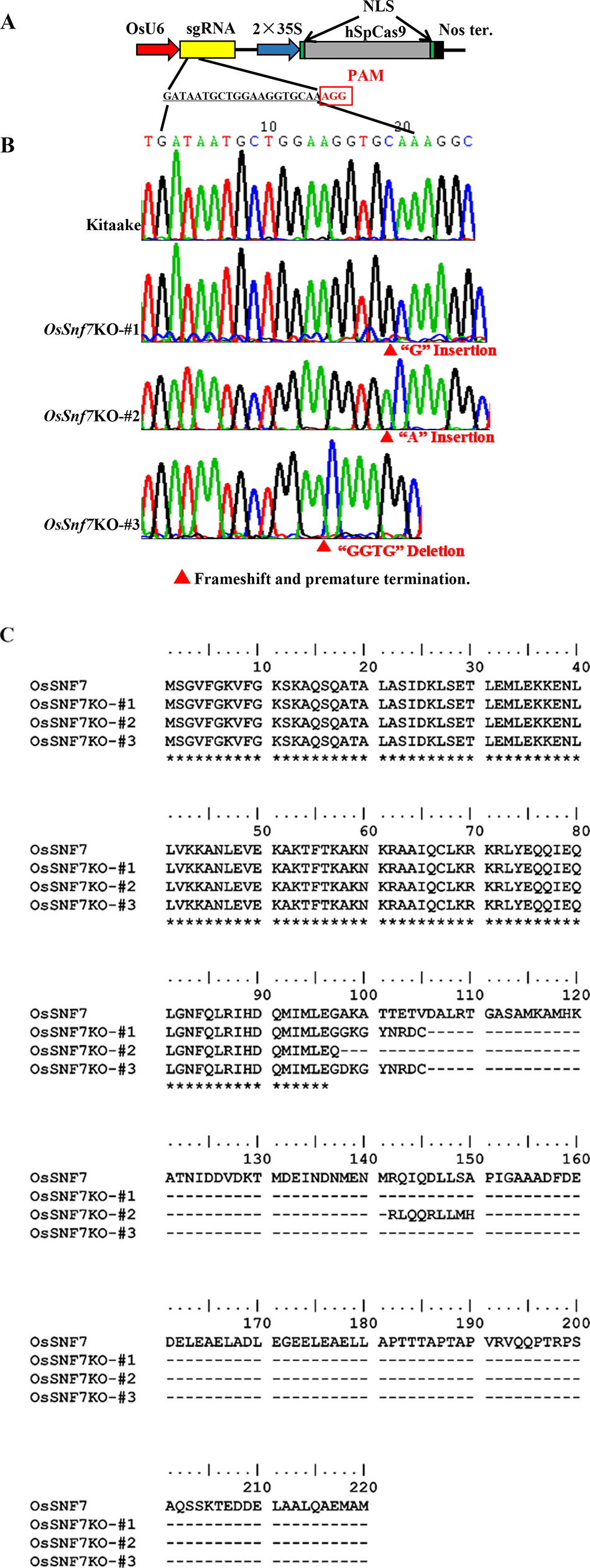

Supplement: S28 Fig — (A) Schematic of the OsSnf7Cas9 construct. Key elements and the sgRNA sequence for specifically targeting OsSnf7 are respectively indicated. (B) DNA sequencing chromatograms of three OsSnf7 knock-out (OsSnf7-KO) lines. The mutations, ‘G’ insertion in line OsSnf7-KO-#1, ‘A’ insertion in line OsSnf7-KO-#2 and ‘GGTG’ deletion in line OsSnf7-KO-#3, which lead to frameshift and premature termination in the CDS of OsSnf7 are respectively indicated. (C) The OsSNF7 protein sequence of the wild type and the three OsSnf7-KO lines predicted. (TIF) [file pgen.1006311.s028.tif]

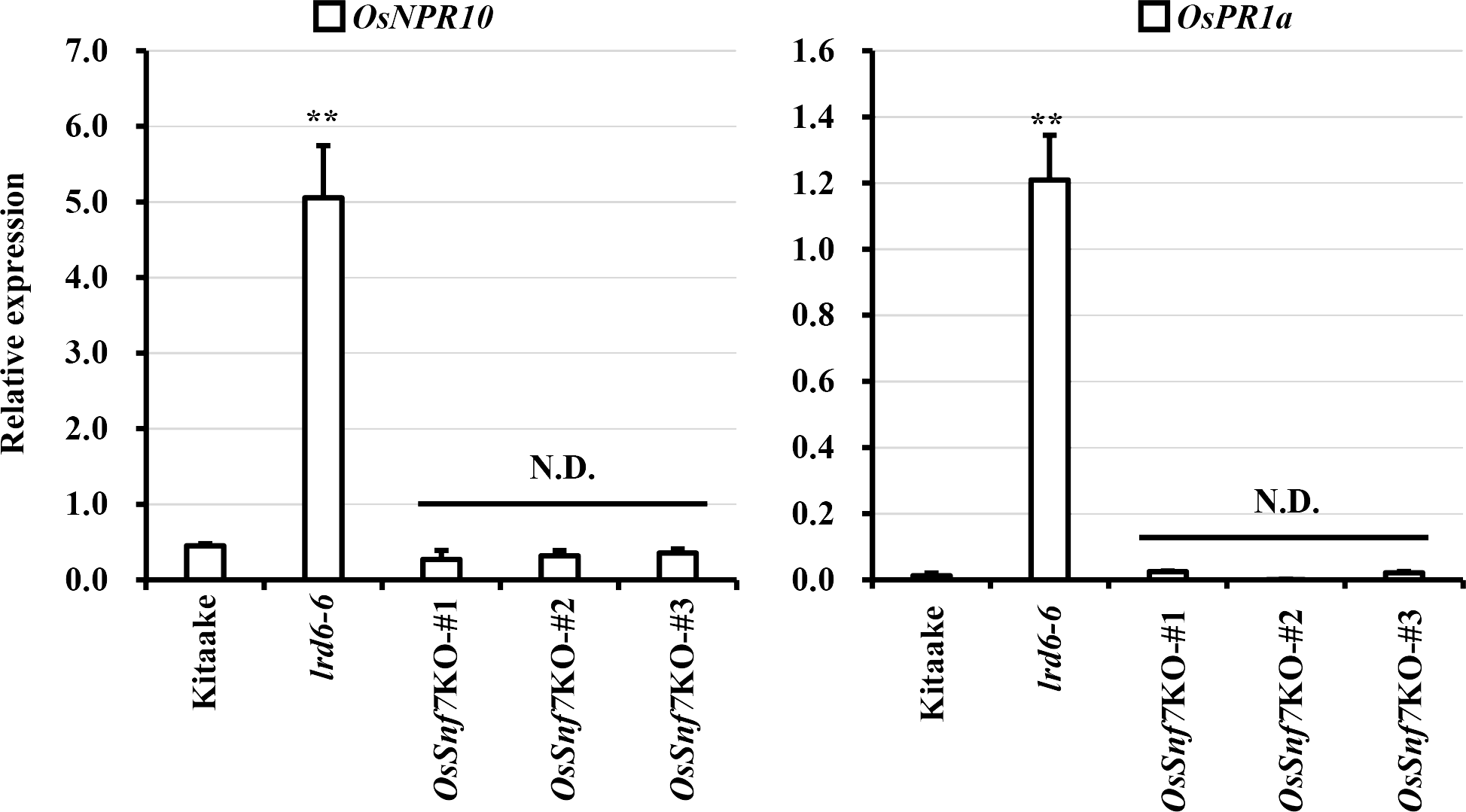

Supplement: S29 Fig — Leaf samples of those lines were collected respectively and subjected to PR genes expression determination by using qRT-PCR. The expression was normalized to the Ubp5 reference gene. The error bars represent the SDs of three biology repeats and the expression differences was determined by Student’s t-test (**, P < = 0.01; N.D., No significantly difference). (TIF) [file pgen.1006311.s029.tif]

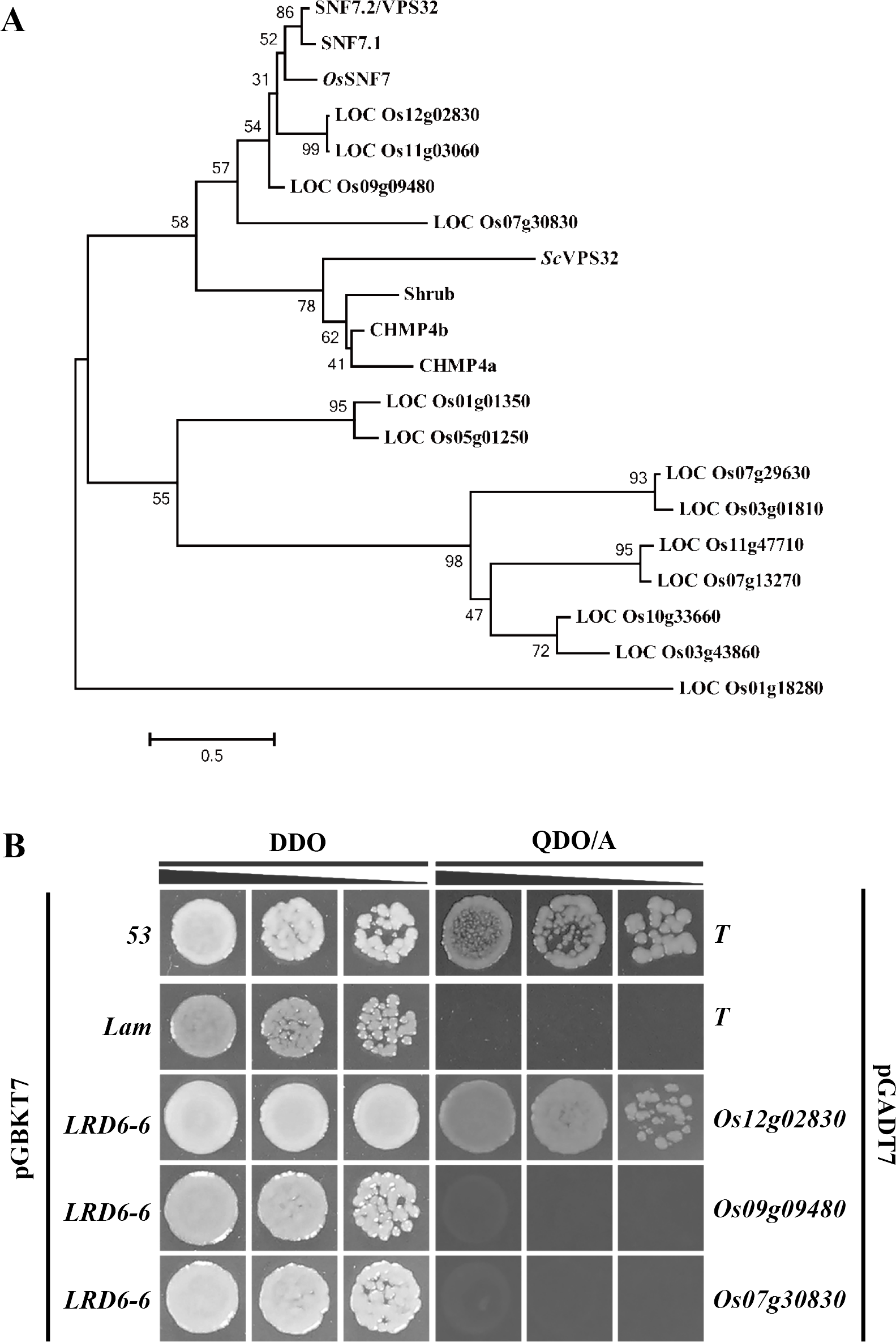

Supplement: S30 Fig — (A) Full amino acid sequences of OsSNF7, CHMP4b, SHRUB, CHMP4a, scVPS32, SNF7.1, SNF7.2 and the rice SNF7 homologs were subjected to alignment and phylogenic analyses using Mega5.1. Bootstrap values are indicated beside each branch. (B) Interaction verification of the LRD6-6 protein with rice OsSNF7 homologs. Among the four homologs with high identity to OsSNF7, three genes, Os12g02830, Os09g09480 and Os07g30830, were successfully cloned and subjected to interaction test in yeast. (TIF) [file pgen.1006311.s030.tif]

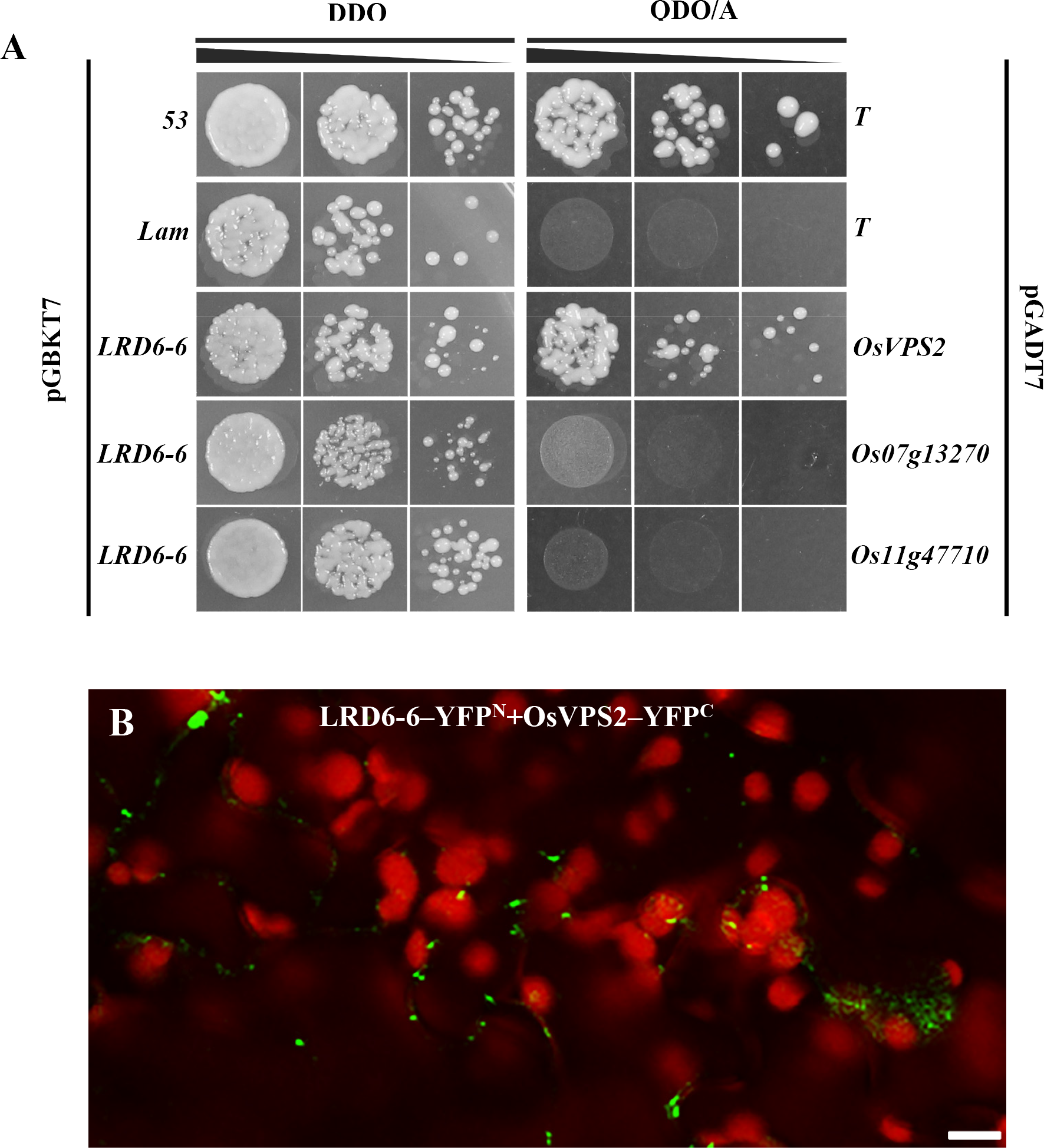

Supplement: S31 Fig — (A) Interaction test in yeast. Three rice genes predicted to code for Arabidopsis VPS2 homologs, OsVps2 (Os03g43860), Os07g13270 and Os11g47710 were cloned and were subjected yeast two hybrid assay. (B) Determination the interaction between LRD6-6 and OsVPS2 by using BiFC approach. Bar = 20 μm. (TIF) [file pgen.1006311.s031.tif]
